# Bromo Analogues of Active 3,4,5,4′-Tetramethoxy-*trans*-stilbene (DMU-212)—A New Path of Research to Anticancer Agents

**DOI:** 10.3390/molecules30244788

**Published:** 2025-12-15

**Authors:** Dawid Łażewski, Gabriela Korzańska, Łukasz Popenda, Karolina Chmaj-Wierzchowska, Artur Korzański, Eduard Potapskyi, Julian Myszkiewicz, Agnieszka Gielara-Korzańska, Agnieszka Zgoła-Grześkowiak, Nataliya Finiuk, Yuliia Kozak, Iryna Ivasechko, Roman Lesyk, Joanna Kuźmińska, Tomasz Goslinski, Marcin Wierzchowski

**Affiliations:** 1 Poznan University of Medical Sciences, Department of Chemical Technology of Drugs, Grunwaldzka 6 Street, 60-780 Poznan, Poland; lazewskidawid@gmail.com (D.Ł.); gabrysia.korzanska@gmail.com (G.K.); potapskiyed@gmail.com (E.P.); julikmyszk@gmail.com (J.M.); agnieszka.gielara@op.pl (A.G.-K.); tomasz.goslinski@ump.edu.pl (T.G.); 2 Adam Mickiewicz University, NanoBioMedical Centre, Wszechnicy Piastowskiej 3 Street, 61-614 Poznan, Poland; 3 Poznan University of Medical Sciences, Department of Maternal and Child Health, 60-701 Poznan, Poland; karolinachmaj@poczta.onet.pl; 4 Adam Mickiewicz University, Department of Chemistry, Uniwersytetu Poznanskiego 8 Street, 61-614 Poznan, Poland; artur.korzanski@amu.edu.pl; 5 Poznan University of Technology, Institute of Chemistry and Technical Electrochemistry, Berdychowo 4, 60-965 Poznan, Poland; agnieszka.zgola-grzeskowiak@put.poznan.pl; 6 Institute of Cell Biology of the National Academy of Sciences of Ukraine, Department of Regulation of Cell Proliferation and Apoptosis, Drahomanov Street 14/16, 79005 Lviv, Ukraine; nataliyafiniuk@gmail.com (N.F.); juliana.kozzak@gmail.com (Y.K.); irynagrytsyna@gmail.com (I.I.); 7 Danylo Halytsky Lviv National Medical University, Molecular Design Center, Pekarska 69, 79010 Lviv, Ukraine; roman.lesyk@gmail.com; 8 Danylo Halytsky Lviv National Medical University, Department of Pharmaceutical, Organic and Bioorganic Chemistry, 79010 Lviv, Ukraine; 9 University of Information Technology and Management in Rzeszow, Medical College, Department of Biotechnology and Cell Biology, Sucharskiego 2, 35-225 Rzeszow, Poland; 10 Poznan University of Medical Sciences, Department of Pharmaceutical Chemistry, Rokietnicka 3, 60-806 Poznan, Poland; jkuzminska@ump.edu.pl

**Keywords:** *trans*-stilbenes, brominated derivatives, anticancer activity, Horner-Wadsworth-Emmons reaction

## Abstract

Stilbenes are a group of polyphenols that are gaining steady attention and have promising biological activity. While much attention is given to polyhydroxy compounds derived from resveratrol, other substituents remain largely unexplored. In this work, we present the results of studies on the synthesis, physicochemical characterisation, and ADME parameters simulation of polymethoxy and brominated stilbenes. We also examined their anticancer activity and found that some of the brominated compounds reveal desirable properties. While the brominated derivatives are not significantly more active than the polymethoxy derivatives, they were found to be safer for the tested pseudo-normal cell lines.

## 1. Introduction

**Figure 1:**
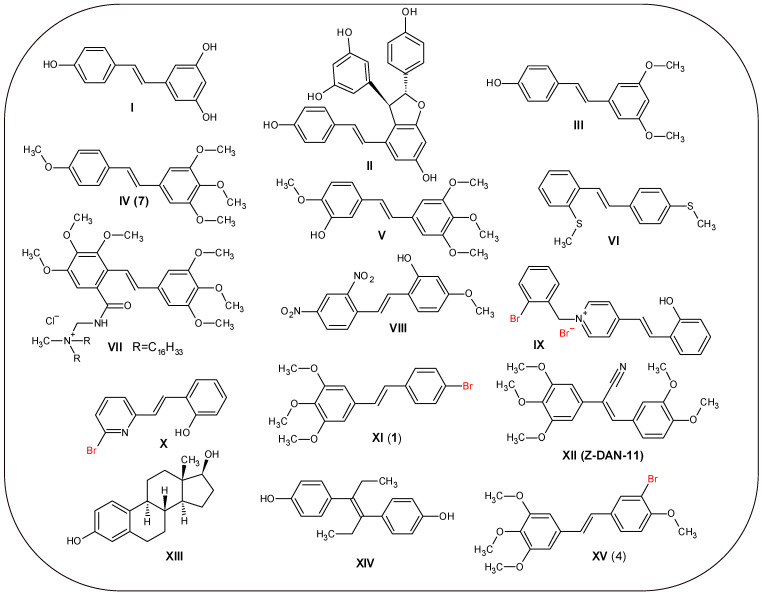
The exemplary chemical structures of *trans*-stilbenes: (**I**) resveratrol (3,5,4′-trihydroxy-*trans*-stilbene), (**II**) ε-viniferin, (**III**) pterostilbene, (**IV**) (**7**) DMU-212 (3,4,5,4′-tetramethoxy-*trans*-stilbene), (**V**) DMU214 (3-hydroxy-3,4,5,4′-tetramethoxy-*trans*-stilbene), (**VI**) 2,4′-dimethylthio-*trans*-stilbene, (**VII**) quaternary amine derivative chloride of 4,5,6,3′,4′,5′-hexamethoxy-*trans*-stilbene, **VIII**) 2-hydroxy-4-methoxy-2′,4′-ditro-*trans*-stilbene, (**IX**) 1-(2-bromobenzyl)-4-[(*E*)-2-(2-hydroxyphenyl)ethenyl]pyridinium bromide, (**X**) 2-bromo-6-[(*E*)-2-(2-hydroxyphenyl)-ethenyl]pyridine, (**XI**) (**1**) 4-bromo-3′,4′,5′-trimetoksy-*trans*-stilbene, (**XII**) Z-DAN-11 ((2*Z*)-3-(3,4-dimethoxyphenyl)-2-(3,4,5-trimethoxyphenyl)prop-2-enenitrile), (**XIII**) estradiol, (**XIV**), diethylstilbestrol, (**XV**) (**4**) 3-bromo-3′,4′,5′,4-tetramethoxy-*trans*-stilbene. Arabic numbers in brackets represent numbering in the experimental part of this work.

A phenomenon dubbed the “French paradox” started an avalanche of interest in *trans*-stilbenoid derivatives. The World Health Organisation in the 1980s launched a ten-year epidemiological research project called MONICA (MONItoring system for CArdiovascular disease) [1]. The project showed that French people presented a relatively low incidence of coronary heart disease, despite their diet rich in saturated fats and similar risk factors when compared with other populations [2]. Further analysis of the data linked this phenomenon to dietary habits—the consumption of small amounts of wine rich in polyphenolic compounds. At the same time, a similar relationship was noted between cancer mortality and wine consumption in eastern France [3]. Among the polyphenols of grape wine, a group of phytoalexins with a *trans*-stilbene structure—resveratrol (Figure 1 **I**) and its derivatives—received special attention. Plants (including vines) primarily produce these compounds in response to various types of infections. Plants can also produce stilbenoids when exposed to other stress factors, such as insect activity or UV radiation [4,5,6]. Resveratrol, as well as its derivatives (Figure 1 e-viniferin **II** and pterostilbene **III**), have been found to have potential to be a drug candidate in clinical trials against such diseases as a wide range of cancer types, obesity, Parkinson’s disease, non-alcoholic fatty liver disease, anxiety, and cognitive disability. The anticancer effects of resveratrol derivatives have various mechanisms of action, ranging from the inhibition of cell proliferation and invasion (colon cancer), inhibition of cell migration (ovarian cancer and melanoma), reverse retinoic acid tolerance (thyroid cancer), and induction of apoptosis (breast cancer, ovarian cancer, and prostatic cancer) [7]. Synthetic polymethoxy analogues of resveratrol have shown even higher anticancer activity. Examples of such compounds are DMU-212—3,4,5,4′-*trans*-tetramethoxystilbene **IV** and its metabolite DMU-214 (Figure 1 **V**). The anticancer effect of DMU-212 is due to its ability to upregulate such factors as p21, p53, cyclin B1, caspase-3 and caspase-9, Bax, ERK1/2, and downregulate Bcl-2 and MEK1/2 [8]. Tetramethoxy-*trans*-stilbene **IV** can be treated as a prodrug, because it is transformed, by oxidation caused by the overexpressed cytochrome P450 CYP1A1 isoform in tumour cells, to the more active DMU-214 [9,10]. DMU-214 metabolite activity was confirmed not only in 2D in vitro studies, but also in 3D, like the Spheroid patient-derived ovarian cancer model (IC_50_ in 2D culture and liposomal drug delivery system was 22 nM, whereas in 3D model SK-OV-3 and patient P3 cell lines, it was 2.9–14.1 mM and 0.5–22.9 mM, respectively) [11]. At the molecular level, its biological and antioxidant activity is mediated by the presence of hydroxyl groups. The distribution of these groups in aromatic rings, within the stilbene scaffold, makes it similar in chemical structure to endogenous and synthetic estrogens such as estradiol (E2) **XIII** or diethylstilbestrol **XIV** [12].

The introduction of substituents other than hydroxyl and methoxyl groups can also result in active *trans*-stilbene derivatives. The sulphur derivative, 2,4-dimethylthio-*trans*-stilbene **VI**, showed inhibition activity (IC_50_ 26 nM) against the P450 CYP1B1 isoform. This cytochrome is responsible for the procarcinogenic metabolism of estradiol (E2) to 4-hydroxy-E2 metabolite, resulting in the development of hormone-induced cancers [13]. Quaternary amine derivative chloride **VII** showed higher antitumor activity against cancer lines (HeLa, MDA-MB-231, and MCF-7) than resveratrol and tamoxifen [14]. Another derivative, the dinitro-derivative **VIII,** induced apoptosis with sub-G1 or S cell cycle arrest in cervical cancer HeLa and glioblastoma U87 (characterised by the value of IC_50_ 6.97 mM and 55.76 mM, respectively) [15].

An intriguing and little-explored possibility for the structural modification of *trans*-stilbene derivatives is the introduction of halogen substituents, particularly bromine atoms. The introduction of such substituents can result in the formation of halogen-type bonds with molecular targets or allow the use of isotopes such as ^77^Br as potential Auger electron emitters in radiopharmaceuticals [16,17]. This introduction reflects a growing pattern in modern medicinal chemistry. Among the 50 medicinal substances approved by the FDA in 2024, as many as 16 contained halogen atoms in their structure, including two bromine derivatives—danicopan (used in paroxysmal nocturnal hemoglobinuria) and aprocitentan (used in resistant hypertension) [18]. A similar strategy in the search for new flavonoids with antidiabetic and anti-glycation properties was successfully applied by Hairani et al. [19]. They discovered that the introduction of bromine into flavonoids could benefit antidiabetic and anti-glycation effects due to the influence of the electronic effect and hydrophobic properties of bromine atoms. An example of the antimicrobial use of a brominated derivative of *trans*-stilbene is *N*-alkylpyridinium bromide derivative **IX**, which was designed for environmental applications and shows promising activity against *Staphylococcus aureus* 209P [20]. The antifungal activity of derivatives, such as the pyridine analogue **X**, was described by Chandrasekara Reddy et al. Derivative **XI** showed a selective MIC value of 25 μg/mL toward *Mucor indicus*, similar to Nystatin [21,22].

Promising anticancer activity was revealed by 4-bromo-3′,4′,5′-trimethoxy-*trans*-stilbene **XI** (**1**), which induced arrest at the G2/M phase of the cell cycle with an IC_50_ value of 6.36 μM in cultured human lung cancer cells (A549). In addition, the *Z* isomer of compound **XII** showed excellent activity expressed by the IC_50_ parameter equal to 0.03 μM, which indicates the group of *cis*-stilbene isomers as a source of potential leading compounds [23,24]. Finally, compound **XV** (**4**)—3-bromo-3′,4′,5′,4-tetramethoxy-*trans*-stilbene appears to be a very promising lead compound among bromoderivatives. It was characterised by good GI_50_ values for such cancer cell lines as pancreas BXPC-3 (0.34 μg/mL), neuroblast SK-N-SH (0.40 μg/mL), lung-NSC NCI-H460 (0.36 μg/mL), and prostate DU-145 (0.45 μg/mL) [25].

In light of the above, the introduction of halogen substituents, including bromine atoms, may be a promising strategy for structural modification in the search for new lead compounds from the stilbenoid group.

## 2. Results and Discussion

### 2.1. Synthesis

**Figure 2:**
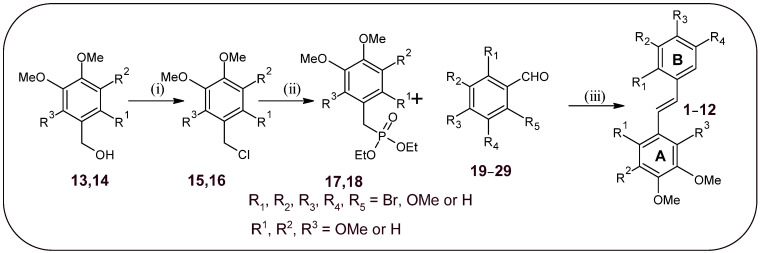
Scheme of synthesis of compounds **1**–**12**. Reaction conditions: (i) SOCl_2_, CHCl_3_, 0–25 °C, 1 h; (ii) P(OEt)_3_, 130 °C, 24 h; (iii) NaH, DMF, r.t., 1 h, next 100 °C, 1.5 h. The letters “A” and “B” indicate the designation of the aromatic ring.

A three-step synthesis of new bromo-derivatives of *trans*-stilbene was designed by modifications of previously described procedures by Murias et al. [26] and Piekus-Słomka et al. [27] (Figure 2). The synthesis of derivatives **1**, **2**, and **4** has been previously described in the literature, but using other methods or with limited characteristics, confirming the structure of the compound [23,25,28]. The compounds **7**–**12** were synthesised as part of the research work of Mikstacka et al. [29] and sourced from the compound library of the Department of Chemical Technology of Drugs, Poznan University of Medical Sciences.

The synthetic procedure begins with the preparation of trimethoxybenzyl chloride **15** or **16**, which is achieved by converting the appropriate benzyl alcohol **13** or **14** with SOCl_2_. In the intermediate step, chloride derivatives **15** or **16** were converted to substrates **17** or **18** (diethyl [trimethoxybenzyl]phosphonate) via the Michaelis–Arbuzov reaction, respectively. This process was practically quantitative. In the last step, bromoderivatives of aldehydes (**19**–**29**) were condensed with **17** or **18** using the Horner–Wadsworth–Emmons reaction to yield final products **1**–**12**. The required *trans*-stilbenes were purified and separated from the *cis*-stilbenes present in trace amounts by recrystallisation from 96% ethanol. The structures of all compounds were confirmed by NMR (^1^H NMR, ^13^C NMR, ^1^H-^1^H COSY, ^1^H-^13^C HSQC, and ^1^H-^13^C HMBC), MS (ESI), and purity by HPLC techniques. Table 1 shows the position of substituents in the *trans*-stilbene scaffold of the synthesised compounds. The yield of the reaction to obtain bromo-*trans*-stilbenes was similar to that described for previously obtained methoxy derivatives, and it was approximately 50%. The exception is derivative **6**, which surprisingly degraded under condensation reaction conditions.

### 2.2. NMR Study

**Figure 3:**
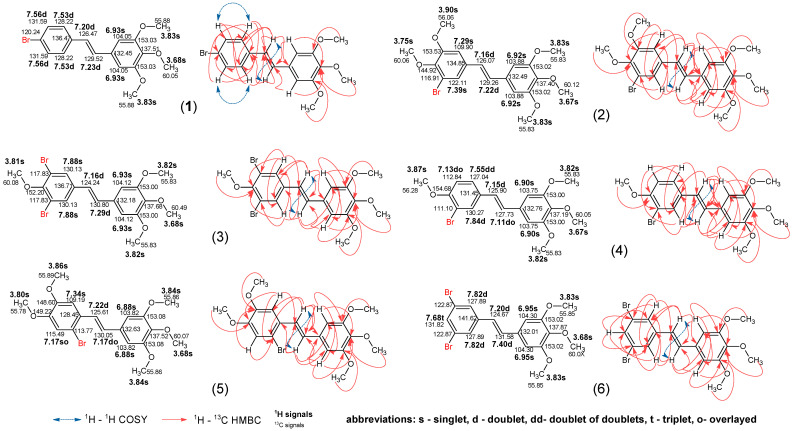
The annotated ^1^H and ^13^C signals of bromine derivatives **1**–**6** were determined in DMSO-*d*_6_. The blue (^1^H-^1^H COSY) and red (^1^H-^13^C HMBC) arrows represent correlations observed in 2D NMR experiments.

Stilbene’s structure consists of two phenyl rings linked by an alkenyl group. In the case of this work, all isomers are in the *E* configuration. For this analysis, we will refer to the ring with three methoxyl groups, “A”, and the other ring, containing bromine, “B” Detailed signal annotation is presented in Figure 3. In all of the synthesised stilbenes, the A ring is very similar. It is also symmetrical, with both aromatic protons having one singlet signal at 6.90–6.95 ppm, while carbons are at 103.75–104.30 ppm. The two methoxyl groups hydrogens at positions 3 and 5 relative to the alkenyl group also overlap due to symmetry as a singlet signal at 3.82–3.83 ppm with carbon signals at 55.83–55.85 ppm. Ring carbons connected to these groups are highly shifted to 153.00–153.02 ppm. Lastly, the methoxyl group protons of the A ring are shifted slightly to 3.67–3.68 ppm, while the carbon signal is observed at 60.05–60.12 ppm. Interestingly, the ring carbon connected to this group, with a signal at 137.19–137.87 ppm, is not as heavily shifted as the other methoxy-connected atoms and is more similar to the carbon connected to the alkenyl group, at 132.01–132.76 ppm. The alkenyl linker signals behave differently, which is due to the presence of various substituents in ring B. While the atoms closer to the B ring behave somewhat similarly between different compounds, with a proton doublet at 7.15–7.22 ppm and carbon at 124.24–126.47, the other atoms in this group have much more variability. The proton ranges from 7.11 ppm in compound **4** to 7.40 ppm in compound **6**. Similarly, the carbon signals range from 127.73 ppm to 131.58 ppm. We also noted the J-coupling constant of 16 Hz in alkenyl protons, thereby confirming the *E* configuration of the double bond. In the B ring of the brominated stilbenes, there is, as expected, considerable variability due to differences in structure; however, there are some general observations. The methoxy groups behave similarly to the symmetrical groups from the A ring, no matter their position and number. The protons appear as singlets at 3.75–3.90 ppm and their carbons at 55.78–60.08 ppm. Meanwhile, the aromatic carbons connected to these groups exhibit the same high shifts as in ring A at 144.92–154.66 ppm. The opposite is observed for carbons with bromine substituents. They exhibit a shift to smaller values ranging from 111.10 ppm to 122.87 ppm. The aromatic protons of ring B, in general, have higher values in ppm when they are closer to bromine atoms and lower values when they are closer to methoxyl groups. The same principle applies to their respective carbon atoms.

### 2.3. Mass Spectrometry

Aside from the standard MS ESI experiment, we also analysed the fragmentation of the molecular ions, the results of which are shown below.

**Figure 4:**
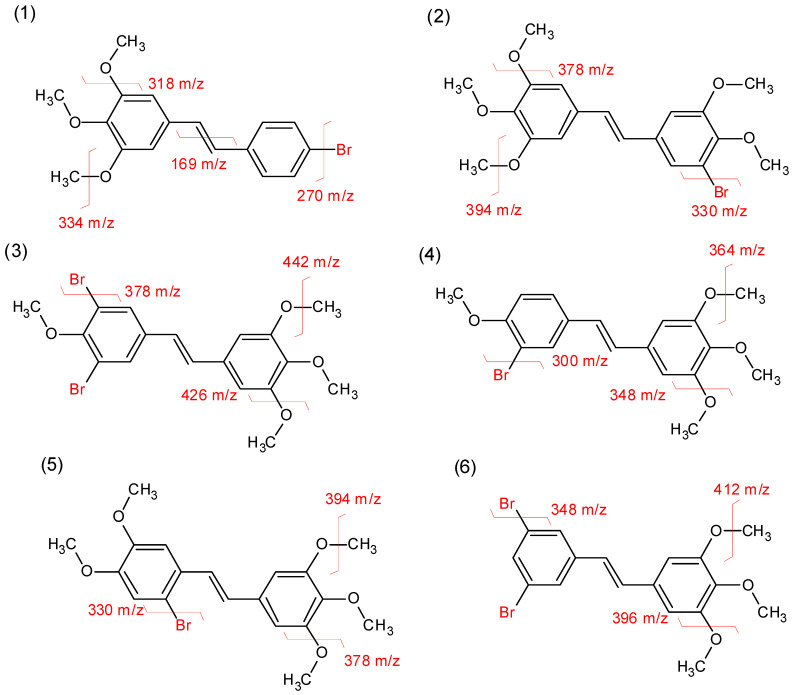
The observed fragmentation of bromostilbene compounds **1**–**6**.

As shown in Figure 4 below, we can observe the expected fragmentation from the structures. Each of the brominated compounds exhibits a specific peak distribution profile, which is dependent on the number of bromine atoms in the structure and the presence of its two natural isotopes (^79^Br, ^81^Br). The most common fragmentation patterns involve the breaking of one bond, either between the bromine and the phenyl ring or within the methoxyl group. Only in one case, in the brominated stilbenes, did we note ions corresponding to the breaking of the double bond. The mass spectrum of compound **1** shows two main peaks at [M+H]^+^ (^79^Br) 348.9 *m*/*z*, (^81^Br) 350.8 *m*/*z*. While the fragmentation spectrum shows primarily peaks at 270 *m*/*z*, 318 *m*/*z*, and 334 *m*/*z*, which correspond to the elimination of bromine, methoxyl, and methyl groups, respectively, this compound is the only one with a strong peak at 169 *m*/*z* corresponding to the CH-C_6_H_4_-Br ion, but interestingly, there is no signal from the other half of the fragmented molecule. The mass spectrum of compound **2** shows two main peaks at [M+H]^+^ (^79^Br) 409.1 *m*/*z*, (^81^Br) 411.0 *m*/*z*. The fragmentation pattern primarily shows peaks at 330 *m*/*z*, 394 *m*/*z*, and 378 *m*/*z*, corresponding to the elimination of bromine, methyl, and methoxyl groups, respectively, as well as a peak at 315 *m*/*z*, indicating the elimination of both bromine and methyl groups. Mass spectrum of stilbene **3** shows characteristic mass distribution stemming from two bromine atoms [M+H]^+^ (^79^Br, ^81^Br) 458.9 *m*/*z*, (2x ^81^Br) 461.0 *m*/*z*, (2x ^79^Br) 457.2 *m*/*z*. While the masses of fragmented ions are different depending on the remaining bromine atom in the ion, the pattern is the same. The main fragmentation ions visible on the spectrum correspond to the elimination of the methoxyl, methyl, and bromine moieties. The mass spectrum of compound **4** shows two main peaks at [M+H]^+^ (^79^Br) 379.0 *m*/*z*, (^81^Br) 380.9 *m*/*z*. The fragmentation spectrum primarily shows peaks at 300 *m*/*z*, 348 *m*/*z*, and 364 *m*/*z*, which correspond to the elimination of bromine, methoxyl, and methyl groups, respectively. The mass spectrum of stilbene **5** is nearly identical to that of compound **2**, as they are isomers with the same mass and fragmentation pattern. Mass spectrum of compound **6**, just as compound **3**, shows characteristic mass distribution stemming from two bromine atoms [M+H]^+^ (^79^Br, ^81^Br) 428.9 *m*/*z*, (2x ^81^Br) 430.9 *m*/*z*, (2x ^79^Br) 427.0 *m*/*z*. The fragmentation spectrum also follows the same principle as compound **3**.

### 2.4. X-Ray Diffraction Studies

**Figure 5:**
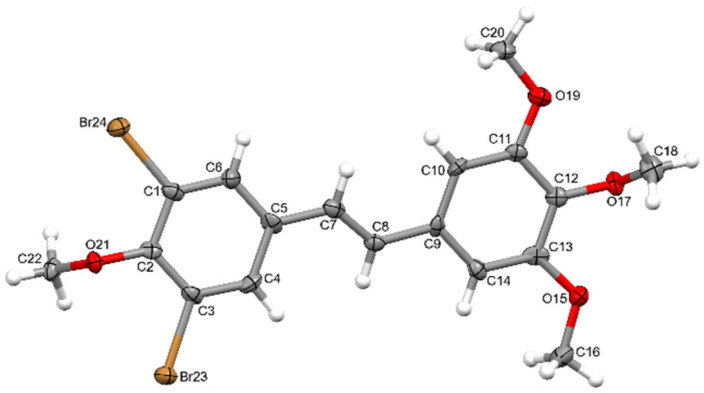
Anisotropic ellipsoid representation of 3,5-dibromo-3′,4′,5′,4-tetramethoxy-*trans*-stilbene **3**, showing the atom-labelling scheme. Displacement ellipsoids are drawn at the 50% probability level, and H atoms are shown as small spheres of arbitrary radii.

The crystal structure of 3,5-dibromo-3′,4′,5′,4-tetramethoxy-*trans*-stilbene was determined by single-crystal X-ray diffraction. Crystals were grown from DMF by slow evaporation of the solution. The crystal structure of **3** is monoclinic with the P2_1_ space group. The asymmetric unit of **3** contains one molecule. Atom labelling of **3** is shown in Figure 5.

**Figure 6:**
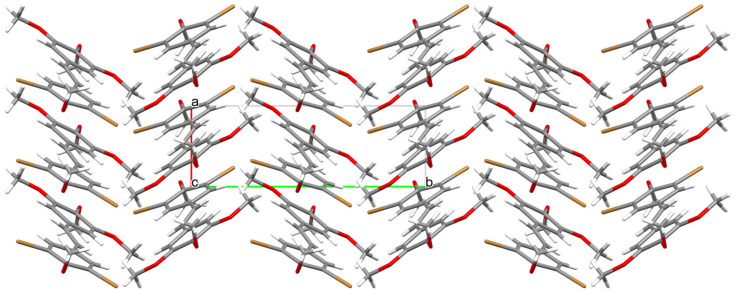
Packing of molecules 3,5-dibromo-3′,4′,5′,4-tetramethoxy-*trans*-stilbene **3** along the direction [001].

**Figure 7:**
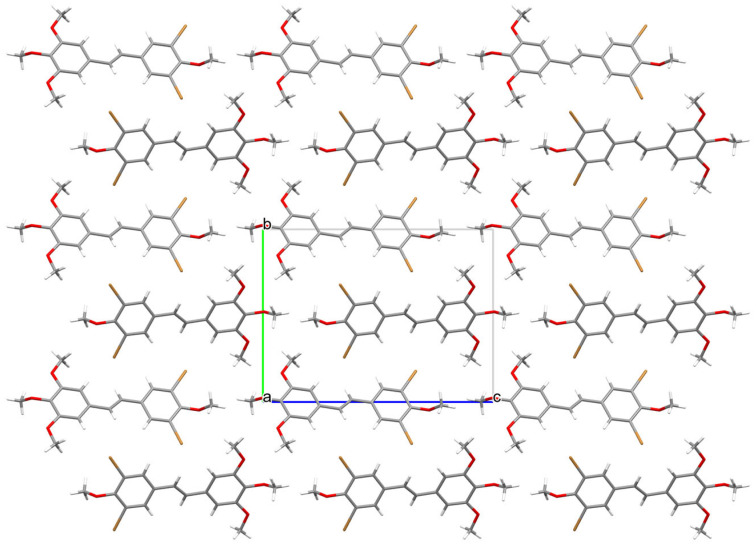
Packing of molecules 3,5-dibromo-3′,4′,5′,4-tetramethoxy-*trans*-stilbene **3** along the direction [100].

**Figure 8:**
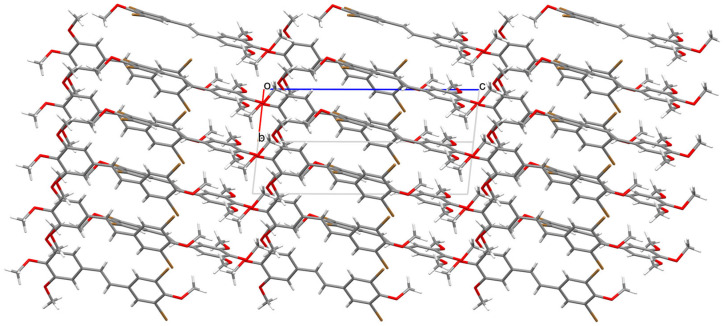
Packing of molecules 3,5-dibromo-3′,4′,5′,4-tetramethoxy-*trans*-stilbene **3** along the direction [110].

Analysis of the X-ray data showed that the molecule is not planar. The C(1)-C(6) and C(9)-C(14) rings are deviated from the plane formed by the C(5)-C(7)-C(8)-C(9) atoms of the molecule by 17.21 (1.46) and 9.24 (1.69) degrees, respectively. The methoxy groups O(19)-C(20) and O(15)-C(16) lie in the ring plane, as indicated by the fact that torsional angles C(14)-C(13)-O(15)-C(16) and C(10)-C(11)-O(19)-C(20) are 0.62(1.42) and 3.49(1.40) degrees, respectively. The remaining O(21)-C(22) and O(17)-C(18) methoxy groups are significantly deviated from the ring plane, as indicated by C(22)-O(21)-C(2)-C(1) and C(18)-O(17)-C(12)-C(11) torsion angles of 95.46(1.11) and 72.61(1.25) degrees, respectively. In the crystal structure of **3**, weak interactions of C-H···O, C-H···C, and Br···C type connect the molecules in a three-dimensional network (Figure 6, Figure 7 and Figure 8).

### 2.5. Biological Activity

As shown in Table 2, none of the tested stilbenes exhibit activity against the U251 (human malignant glioblastoma) and HT-29 (human colorectal carcinoma) cell lines. Regarding the neoplastic cells, only the Jurkat (human acute T-cell leukaemia) is affected. Out of all the tested compounds, **5** shows an IC_50_ value below 10 µM. However, only three of them show desirable activity parameters. Namely, pentamethoxy compounds **8**, **10** (3,3′,4,4’,5-pentamethoxy-*trans*-stilbene and 2,3’,4,4’,5-pentamethoxy-*trans*-stilbene) and bromotetramethoxy compound **4** (3-bromo-3’,4’,5’,4-tetramethoxy-*trans*-stilbene) exhibit low IC_50_ values against neoplastic cells while simultaneously being safer for the control cell lines. The remaining compounds either exhibit very low activity or high activity but a low therapeutic index (TI), as is the case with the compounds described earlier, **1** and **7** (**DMU-212**). From the analysis, we can deduce that for optimal activity, the presence of at least two methoxyl groups in one ring in positions *para* and *meta* is crucial. The second ring seems to be much more flexible. The presence of bromine atoms is largely detrimental to either activity or TI. From this, we can deduce that non-brominated methoxy stilbenes are generally more active against cancer cells tested here. This is also supported by literature, as there are more examples of methoxy stilbenes reported than halogenated ones [12,29,30,31].

A direct comparison of the substitution of methoxy and hydrogen substituents with bromine shows a varied effect on anticancer activity. A comparison of compounds **1**, **4**, and **7** shows that the introduction of bromine to a *meta* position in ring B maintains high activity against the Jurkat cell line and reduces cytotoxicity against the pseudo-normal J744.2 cell line. However, changing the methoxy group in the *para* position to bromine lowers activity in all lines. Unfortunately, in the case of other analogues, such as pairs of compounds **2** and **9**, just as **6** and **12**, we observe a somewhat reverse effect. Here, changing the methoxy group to bromine in the *meta* position (either one or two) results in a significant increase in cytotoxic effect on pseudo-normal cells. Interestingly, the active compound **7** differs from the inactive compound **2** only by the presence of an additional -OCH_3_ substituent at position 5 of ring B, suggesting a spatial restriction at the binding site in the molecular target.

**Figure 9:**
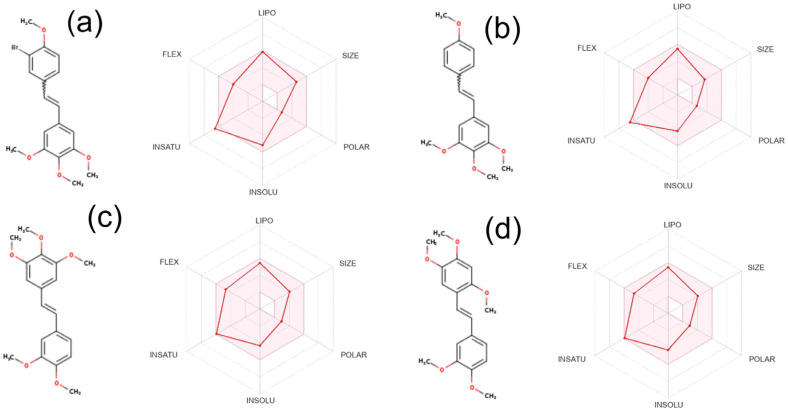
Bioavailability Radar plots for compounds: (**a**) **4**, (**b**) **7**, (**c**) **8**, and (**d**) **10**. Parameters named LIPO, SIZE, POLAR, INSOLU, and INSATU represent lipophilicity, size, polarity, solubility, flexibility, and saturation, respectively.

Drug-likeness analysis was performed in the SwissADME web system, which allows for the computation of physicochemical descriptors as well as the prediction of ADME parameters, pharmacokinetic properties, druglike nature, and medicinal chemistry friendliness of one or multiple small molecules to support drug discovery [32,33]. Full data for all analysed compounds are present in the Supplementary Materials (Figures S56–S67 and Tables S5–S13). The SwissDrugDesign calculation module determined parameters such as lipophilicity, size, polarity, solubility, flexibility, and saturation, which are presented in the form of a bioavailability radar for the most active compounds in Figure 9. All parameters predicted by the predictive model are close to the reference values for the **DMU-212** (**7**) group and are within the optimal range, with the exception of the slightly exceeding INSATU parameter. Among the molecular targets identified by the predictive model, those with a high probability (above 0.3) of interaction are structural proteins—tubulin beta-1 chain (**3**, **4**, **7**) and tubulin beta-3 chain (**7**); primary active transporter P-glycoprotein 1 (**7**, **8**); and cytochromes P450—CYP 1B1 (**7**, **10**) and CYP 1A1 (**10**). Among the less probable molecular targets for the studied group of bromo-derivatives are aryl hydrocarbon receptor (AhR), cytochrome CYP 1B1, β-amyloid A4, and cyclooxygenases 1 and 2, while for methoxy-derivatives, these are quinone reductase 2, HMG-CoA reductase, and cytochrome CYP 1A2.

The molecular targets proposed by the predictive model, namely beta-1 chain tubulin and beta-3 chain tubulin, appear to be very interesting. Literature reports published by Parida et al. indicate a similar compound, Z-DAN-11 (Figure 1 **XII**), which has been shown to inhibit cancer progression by disrupting microtubule dynamics, driving G2/M arrest, and inducing p53-dependent apoptosis [34].

**Figure 10:**
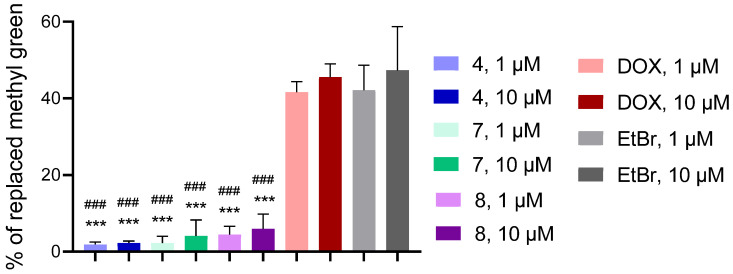
The percentage of methyl green dye replaced from its complex with DNA by the studied derivatives **4**, **7**, **8**, and reference compounds—DOX and EtBr, all at 1 and 10 μM. ***—*p* < 0.001 significant changes compared with the effect of DOX (1 μM); ###—*p* < 0.001 significant changes compared with the impact of EtBr (1 µM). Data are presented as mean ± standard deviation.

To investigate the potential mechanisms of cell death induced by the studied derivative, we evaluated apoptosis using the fluorescence microscopy of cells stained with Hoechst-33342 and Ethidium Bromide (EtBr), as well as spectroscopic and DNA/methyl green displacement assays. Methyl green is a cationic dye that binds preferentially to the phosphate backbone of DNA (primarily in the major groove). If a compound (an intercalator or groove-binding agent) is added, it can compete with methyl green for binding to DNA. The higher the percentage of substituted methyl green, the stronger the compound interacts with DNA. Intercalation is the key feature of clinically used antitumor drugs, e.g., doxorubicin (DOX). DOX displaced 41.62–45.52% of methyl green from its complex with DNA (Figure 10). Another intercalator, EtBr, replaced 42.12–47.35% of methyl green from its complex with DNA.

The studied compounds were less active in such replacement, compared with DOX and EtBr. Derivatives **4**, **7**, and **8** in 1 and 10 μM doses replaced the methyl green dye from the DNA-methyl green complex by 1.87–5.98% (Figure 10). Thus, compounds **4**, **7**, and **8** have low affinity for DNA.

The relationship between the structure and activity of curcuminoids and bromo-analogues of the active 3,4,5,4′-tetramethoxy-*trans*-stilbene, backed by cytotoxicity data showing their varied impact on cell survival, is highlighted in fluorescence images of Jurkat cells.

**Figure 11:**
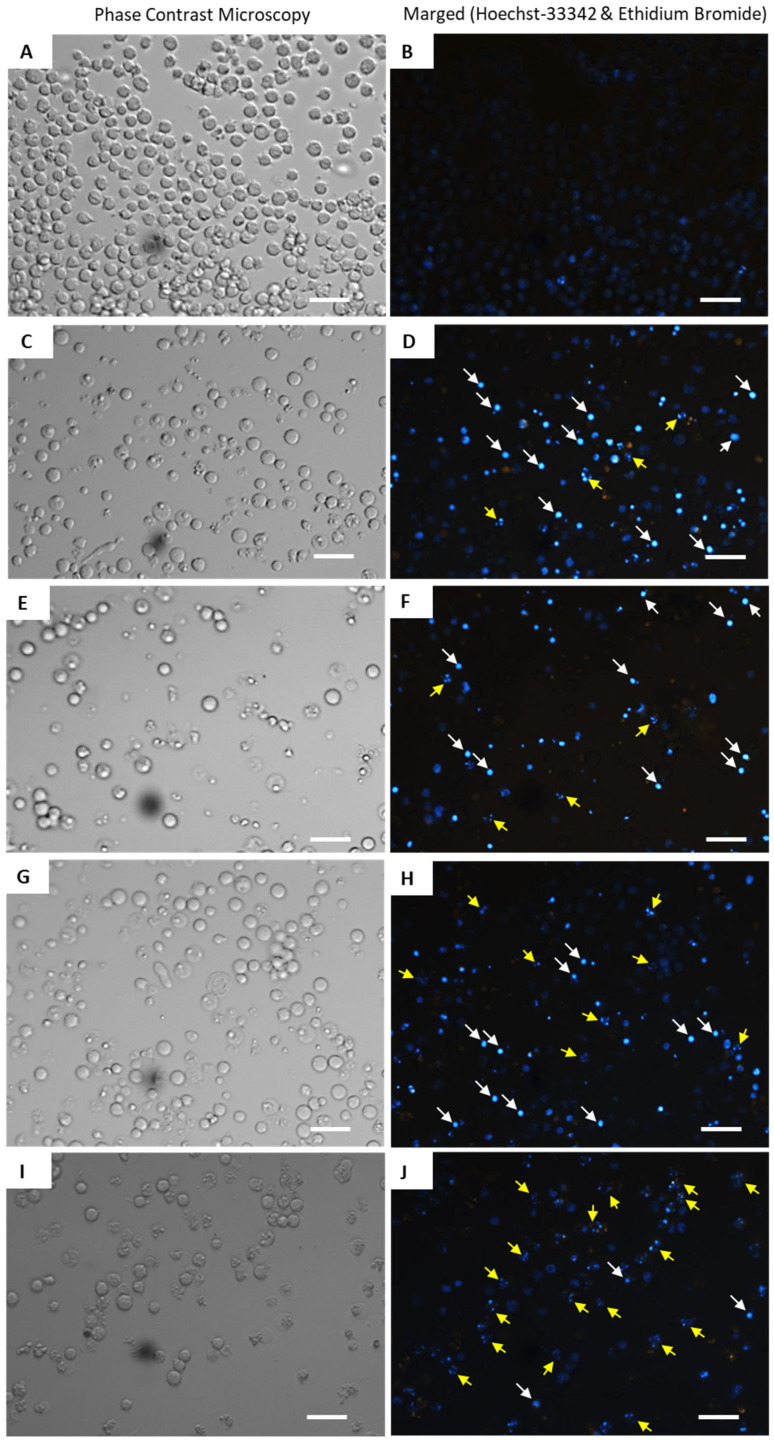
Fluorescence photomicrographs of Jurkat cells stained with Hoechst 33342 and EtBr after 24 h exposure to the test compounds: (**A**,**B**) untreated control cells; (**C**,**D**) cells treated with compound **4** (0.5 µM); (**E**,**F**) cells treated with compound **7** (0.5 µM); (**G**,**H**) cells treated with compound **8** (0.5 µM); (**I**,**J**) cells treated with doxorubicin (0.5 µM). Scale bar—20 µm.

Jurkat cells treated with the most potent compounds for 24 h and stained with Hoechst 33342/EtBr demonstrated structure-dependent effects on cell morphology and viability. Control cells (Figure 11A,B) exhibited blue Hoechst 33342 staining with minimal orange-red EtBr signal, reflecting intact nuclei and a healthy state. At 0.5 µM, compounds **4** and **8** induced moderate apoptosis, with fewer EtBr-positive (necrotic) cells than doxorubicin, alongside chromatin condensation (white arrows) and DNA fragmentation (yellow arrows) (Figure 11C,D,G,H). Compound **7** (0.5 µM) markedly reduced viable cell numbers and enhanced nuclear fragmentation (Figure 11E,F). Doxorubicin (0.5 µM), as a positive control, elicited a similar morphological disruption and chromatin fragmentation as compound **8** did (Figure 11I,J).

Overall, the tested compounds triggered chromatin condensation and DNA fragmentation, indicating pro-apoptotic activity, with limited progression to necrosis, as shown by weak EtBr uptake.

## 3. Materials and Methods

All substrates used in synthesis were purchased from either TCI or Merck. Solvents were purchased from either Chempur (Piekary Śląskie, Poland), Avantor (Gliwice, Poland), or Stanlab (Lublin, Poland). ESI-MS spectra were registered using an Agilent Technologies 6410B Triple Quadrupole LC/MS System with an Agilent 1200/1290 HPLC (Agilent Technologies, Santa Clara, CA, USA). NMR spectra were recorded at 298 K on an Agilent DD2 800 spectrometer (Agilent Technologies, Santa Clara, CA, USA) equipped with a 5 mm ^1^H(^13^C/^15^N) probe head. UV–vis spectra were recorded on a UV–Vis Jasco V-770 spectrophotometer (JASCO, Tokyo, Japan). HPLC determined compound purity on an Agilent 1260 Infinity II chromatograph (Agilent Technologies, Santa Clara, CA, USA). Melting points (M.p.) were determined using a “Stuart” apparatus (Bibby Sterlin Ltd., Staffordshire, UK), with single-ended capillaries.

### 3.1. Crystallographic Measurements

**Figure 12:**
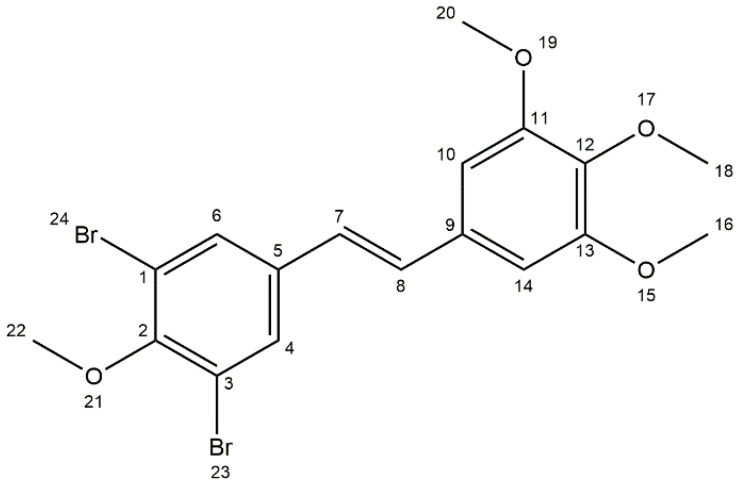
Atom numbering scheme for compound (**3**).

A colourless, post-shaped crystal was mounted on the goniometer. Data for 3,5-dibromo-3′,4′,5′,4-tetramethoxy-*trans*-stilbene were collected from a single crystal at 100K on a Bruker D8 VENTURE KAPPA (Bruker, Billerica, MA, USA) diffractometer with a microfocus sealed tube using a multilayer mirror as monochromator and a Bruker PHOTON III CPAD detector(Bruker, Billerica, MA, USA). The diffractometer was equipped with an Oxford Cryostream 600 low-temperature device (Oxford Cryosystems Ltd., Long Hanborough, UK) and used Mo *K_α_* radiation (λ = 0.71073 Å). All data were integrated with SAINT V8.41, yielding 12,582 reflections of which 3123 were independent (average redundancy 4.03), and 96.1% were greater than 2σ(*F*^2^) [35]. A Multi-Scan absorption correction using SADABS 2016/2 was applied [36]. The structure was solved by dual methods with SHELXT 2018/2 and refined by full-matrix least-squares methods against *F*^2^ using XL [37,38]. All non-hydrogen atoms were refined with anisotropic displacement parameters. All hydrogen atoms were refined isotropically on calculated positions using a riding model with their *U*_iso_ values constrained to 1.5 times the *U*_eq_ of their pivot atoms for terminal sp^3^ carbon atoms and 1.2 times for all other carbon atoms. Interpretation of the results was performed using the SHELXTL (version 2018/2) [37] and Mercury 2024.2.0 (Build 415171) [39] programmes. The crystal and refinement data are given in Table 3 and atom numbering in Figure 12. Crystallographic data for the structures reported in this paper have been deposited with the Cambridge Crystallographic Data Centre [40]. CCDC 2499084 contains the supplementary crystallographic data for this paper. These data can be obtained free of charge from the Cambridge Crystallographic Data Centre via www.ccdc.cam.ac.uk/structures (deposition date 30 October 2025).

### 3.2. Synthesis of Trans-Stilbenes Derivatives

The general procedure for the synthesis of *E*-stilbenes is based on the Horner-Wadsworth-Emmons reaction. It involves the reaction of equimolar amounts of phosphonate, aldehyde, and sodium hydride as a base. The reaction is conducted in dimethylformamide (DMF) as a solvent. Initially, the phosphonate and the base are dissolved/suspended at 0 °C for 30 min. Then, the aldehyde dissolved in DMF is dripped into the suspension. After that, the temperature is allowed to rise to room temperature, and the reaction mixture is left for 24 h. After the reaction is complete, the mixture is poured into water and filtered. The resulting precipitate is recrystallised from ethanol and dried.

 **(1)** 4-bromo-3′,4′,5′-trimethoxy-trans-stilbene

^1^H NMR (800 MHz, DMSO-*d*_6_) δ 7.56 (d, *J* = 8.6 Hz, 2H arom. **H**), 7.53 (d, *J* = 8.6 Hz, 2H arom. **H**), 7.23 (d, *J* = 16.4 Hz, 1H alkenyl **H**), 7.20 (d, *J* = 16.4 Hz, 1H alkenyl **H**), 6.93 (s, 2H arom. **H**), 3.83 (s, 6H -OC**H**_3_), 3.68 (s, 3H -OC**H**_3_).

^13^C NMR (201 MHz, DMSO-*d*_6_) δ 153.03 arom. **C**-OCH_3_, 137.51 arom. **C**-OCH_3_, 136.47 arom. **C**-CH=, 132.45 arom. **C**-CH=, 131.59 arom. **C**-H, 129.52 alkenyl **C**, 128.22 arom. **C**-H, 126.47 alkenyl **C**, 120.24 arom. **C**-Br, 104.05 arom. **C**-H, 60.05 -O**C**H_3_, 55.88 -O**C**H_3_.

ESI [M+H]^+^ (^79^Br) 348.9 *m*/*z*, (^81^Br) 351.8 *m*/*z*.

UV–Vis (CH_2_Cl_2_): λ_max_ [nm] (log ε, [dm^3^·mol^−1^·cm^−1^]): 327, (4.64).

Yield = 55%, R_f_ = 0.64 EtOAc:nHex 1:2, M.p. = 152–154 °C.

 **(2)** 3-bromo-3′,4′,5′,4,5-pentamethoxy-trans-stilbene

^1^H NMR (800 MHz, DMSO-*d*_6_) δ 7.39 (d, *J* = 1.9 Hz, 1H arom. **H**), 7.29 (d, *J* = 1.9 Hz, 1H arom. **H**), 7.22 (d, *J* = 16.4 Hz, 1H alkenyl **H**), 7.16 (d, *J* = 16.4 Hz, 1H alkenyl **H**), 6.92 (s, 2H arom. **H**), 3.90 (s, 3H -OC**H**_3_), 3.83 (s, 6H -OC**H**_3_), 3.75 (s, 3H -OC**H**_3_), 3.67 (s, 3H -OC**H**_3_).

^13^C NMR (201 MHz, DMSO-*d*_6_) δ 153.53 arom. **C**-OCH_3_, 153.02 arom. **C**-OCH_3_, 144.92 arom. **C**-OCH_3_, 137.40 arom. **C**-OCH_3_, 134.85 arom. **C**-CH=, 132.49 arom. **C**-CH=, 129.26 alkenyl **C**, 126.07 alkenyl **C**, 122.11 arom. **C**-H, 116.91 arom. **C**-Br, 109.90 arom. **C**-H, 103.88 arom. **C**-H, 60.12 -O**C**H_3_, 60.06 -O**C**H_3_, 56.06 -O**C**H_3_, 55.83 -O**C**H_3_.

ESI [M+H]^+^ (^79^Br) 409.1 *m*/*z*, (^81^Br) 411.0 *m*/*z*.

UV–Vis (CH_2_Cl_2_): λ_max_ [nm] (log ε, [dm^3^·mol^−1^·cm^−1^]): 329, (4.42).

Yield = 56%, R_f_ = 0.44 EtOAc:nHex 1:2, M.p. = 191–193 °C.

 **(3)** 3,5-dibromo-3′,4′,5′,4-tetramethoxy-trans-stilbene

^1^H NMR (800 MHz, DMSO-*d*_6_) δ 7.88 (s, 2H arom. **H**), 7.29 (d, *J* = 16.4 Hz, 1H alkenyl **H**), 7.17 (d, *J* = 16.4 Hz, 1H alkenyl **H**), 6.92 (s, 2H arom. **H**), 3.82 (s, 6H -OC**H**_3_), 3.81 (s, 3H -OC**H**_3_), 3.68 (s, 3H -OC**H**_3_).

^13^C NMR (201 MHz, DMSO-*d*_6_) δ 153.00 arom. **C**-OCH_3_, 152.20 arom. **C**-OCH_3_, 137.68 arom. **C**-OCH_3_, 136.71 arom. **C**-CH=, 132.18 arom. **C**-CH=, 130.80 alkenyl **C**, 130.13 arom. **C**-H, 124.24 alkenyl **C**, 117.83 arom. **C**-Br, 104.12 arom. **C**-H, 60.49 -O**C**H_3_, 60.08 -O**C**H_3_, 55.83 -O**C**H_3_.

ESI [M+H]^+^ (^79^Br, ^81^Br) 458.9 *m*/*z*, (2x ^81^Br) 461.0 *m*/*z*, (2x ^79^Br) 457.2 *m*/*z*.

UV–Vis (CH_2_Cl_2_): λ_max_ [nm] (log ε, [dm^3^·mol^−1^·cm^−1^]): 329, (4.63).

Yield = 54%, R_f_ = 0.68 EtOAc:nHex 1:2, M.p. = 185–186 °C.

 **(4)** 3-bromo-3′,4′,5′,4-tetramethoxy-trans-stilbene

^1^H NMR (800 MHz, DMSO-*d*_6_) δ 7.84 (d, *J* = 2.2 Hz, 1H arom. **H**), 7.55 (dd, *J* = 8.5, 2.2 Hz, 1H arom. **H**), 7.15 (do, *J* = 16.4 Hz, 1H alkenyl **H**), 7.13 (do, *J* = 8.5 Hz, 1H arom. **H**), 7.11 (do, *J* = 16.4 Hz, 1H alkenyl **H**), 6.90 (s, 2H arom. **H**), 3.87 (s, 3H -OC**H**_3_), 3.82 (s, 6H -OC**H**_3_), 3.67 (s, 3H -OC**H**_3_).

^13^C NMR (201 MHz, DMSO-*d*_6_) δ 154.68 arom. **C**-OCH_3_, 153.00 arom. **C**-OCH_3_, 137.19 arom. **C**-OCH_3_, 132.76 arom. **C**-CH=, 131.45 arom. **C**-CH=, 130.27 arom. **C**-H, 127.73 alkenyl **C**, 127.04 arom. **C**-H, 125.90 alkenyl **C**, 112.84 arom. **C**-H, 111.10 arom. **C**-Br, 103.75 arom. **C**-H, 60.05 -O**C**H_3_, 56.28 -O**C**H_3_, 55.83 -O**C**H_3_.

ESI [M+H]^+^ (^79^Br) 379.0 *m*/*z*, (^81^Br) 380.9 *m*/*z*.

UV–Vis (CH_2_Cl_2_): λ_max_ [nm] (log ε, [dm^3^·mol^−1^·cm^−1^]): 330, (4.49).

Yield = 59%, R_f_ = 0.48 EtOAc:nHex 1:2, M.p. = 142–143 °C.

 **(5)** 2-bromo-3′,4′,5′,4,5-pentamethoxy-trans-stilbene

^1^H NMR (800 MHz, DMSO-*d*_6_) δ 7.34 (s, 1H arom. **H**), 7.22 (d, *J* = 16.2 Hz, 1H alkenyl **H**), 7.17 (d, *J* = 16.2 Hz, 1H alkenyl **H**), 7.17 (s, 1H arom. **H**), 6.88 (s, 2H arom. **H**), 3.86 (s, 3H -OC**H**_3_), 3.84 (s, 6H -OC**H**_3_), 3.80 (s, 3H -OC**H**_3_), 3.68 (s, 3H -OC**H**_3_).

^13^C NMR (201 MHz, DMSO-*d*_6_) δ 153.08 arom. **C**-OCH_3_, 149.22 arom. **C**-OCH_3_, 148.60 arom. **C**-OCH_3_, 137.52 arom. **C**-OCH_3_, 132.63 arom. **C**-CH=, 130.05 alkenyl **C**, 128.45 arom. **C**-CH=, 125.61 alkenyl **C**, 115.49 arom. **C**-H, 113.77 arom. **C**-Br, 109.19 arom. **C**-H, 103.82 arom. **C**-H, 60.07 -O**C**H_3_, 55.89 -O**C**H_3_, 55.86 -O**C**H_3_, 55.78 -O**C**H_3_.

ESI [M+H]^+^ (^79^Br) 409.1 *m*/*z*, (^81^Br) 411.0 *m*/*z*.

UV–Vis (CH_2_Cl_2_): λ_max_ [nm] (log ε, [dm^3^·mol^−1^·cm^−1^]): 339, (4.80).

Yield = 63%, R_f_ = 0.36 EtOAc:nHex 1:2, M.p. = 133–135 °C.

 **(6)** 3,5-dibromo-3′,4′,5′-trimethoxy-trans-stilbene

^1^H NMR (800 MHz, DMSO-*d*_6_) δ 7.82 (d, *J* = 1.7 Hz, 2H arom. **H**), 7.68 (t, *J* = 1.7 Hz, 1H arom. **H**), 7.38 (d, *J* = 16.3 Hz, 1H alkenyl **H**), 7.20 (d, *J* = 16.3 Hz, 1H alkenyl **H**), 6.95 (s, 2H aromatic **H**), 3.83 (s, 6H -OC**H**_3_), 3.67 (s, 3H -OC**H**_3_).

^13^C NMR (201 MHz, DMSO-*d*_6_) δ 153.02 arom. **C**-OCH_3_, 141.62 arom. **C**-CH=, 137.87 arom. **C**-OCH_3_, 132.01 arom. **C**-CH=, 131.82 arom. **C**-H, 131.58 alkenyl **C**, 127.89 arom. **C**-H, 124.67 alkenyl **C**, 122.87 arom. **C**-Br, 104.30 arom. **C**-H, 60.08 -O**C**H_3_, 60.05 -O**C**H_3_, 55.85 -O**C**H_3_, 55.82 -O**C**H_3_.

ESI [M+H]^+^ (^79^Br, ^81^Br) 428.9 *m*/*z*, (2x ^81^Br) 430.9 *m*/*z*, (2x ^79^Br) 427.0 *m*/*z*.

UV–Vis (CH_2_Cl_2_): λ_max_ [nm] (log ε, [dm^3^·mol^−1^·cm^−1^]):328, (4.40).

Yield = 19%, R_f_ = 0.76 EtOAc:nHex 1:2, M.p. = 189–190 °C.

### 3.3. Cytotoxic Activity of the Tested Compounds

The MTT cell viability assay was performed according to a previously reported protocol [41]. Briefly, adherent cells were seeded at a density of 5000 cells per 100 µL, and suspension cells at 25,000 cells per 100 µL, in 96-well plates and incubated overnight. Subsequently, test compounds were added in 100 µL of culture medium, followed by incubation for 72 h. After treatment, 20 µL of MTT solution (5 mg/mL, Sigma-Aldrich, Burlington, MA, USA) was added to each well. The resulting formazan crystals were dissolved in DMSO (Sigma-Aldrich, Burlington, MA, USA), and absorbance was recorded using a BioTek ELx800 Absorbance Reader (BioTek Instruments, Inc., Vinooski, VT, USA). IC_50_ values were calculated by nonlinear regression analysis using GraphPad Prism (version 9.1.1, GraphPad Software, Boston, MA, USA). Experiments were conducted in triplicate. Human leukemia Jurkat T-cells, human glioblastoma U251 cells were kindly provided by Collection at the Institute of Molecular Biology and Genetics, National Academy of Sciences of Ukraine (Kyiv, Ukraine). Murine macrophages of J774.2 line were a generous gift from Prof. Sir John Vane (William Harvey Research Institute, London, UK) provided via Prof. Janusz Marcinkiewicz (Jagiellonian University Medical College, Krakow, Poland). Human colorectal adenocarcinoma cell line HT-29 was kindly provided by Prof. Anna Bielawska (Department of Biotechnology, Faculty of Pharmacy, Medical University of Bialystok, Poland). Murine BALB/3T3 fibroblasts was obtained from the Cell Collection of the R.E. Kavetsky Institute of Experimental Pathology, Oncology and Radiobiology (Kyiv, Ukraine).

### 3.4. DNA Intercalation Assay (Methyl Green Displacement Method)

A 485 μL aliquot of salmon sperm DNA (50 μg/mL, Sigma-Aldrich, Burlington, MA, USA) was incubated with 15 μL of methyl green solution (1 mg/mL, Sigma-Aldrich, Burlington, MA, USA) at 37 °C for 1 h. Subsequently, 500 μL of the test compounds, doxorubicin, or ethidium bromide (each at final concentrations of 1 μM and 10 μM) was added to the preformed methyl green–DNA complex. The samples were further incubated at 37 °C for 2 h in the dark. Absorbance was then recorded at 630 nm [41,42]. GraphPad Prism (version 9.1.1, GraphPad Software, Boston, MA, USA) was used for the results analysis. Experiments were conducted in triplicate. Doxorubicin and ethidium bromide were purchased from Sigma-Aldrich, USA.

### 3.5. The Fluorescent Microscopy of Jurkat Cells

Jurkat cells were seeded in 24-well plates at a density of 250,000 cells/mL and incubated overnight prior to treatment. Cells were then treated for 24 h with compounds **4**, **7**, and **8** (0.5 µM) or doxorubicin (0.5 µM) as a positive control. Following treatment, cells were stained with Hoechst 33342 (0.2–0.5 µg/mL; Sigma-Aldrich, USA) and ethidium bromide (EtBr, 1 µg/mL). Fluorescence imaging was performed using a LIM-400 microscope (LABEX INSTRUMENT LIMITED, Ningbo, China) equipped with an MTR3CCD camera. Images were captured and analysed using ImageView software (version 4.11.21522.20221011).

## 4. Conclusions

The strategy of introducing bromine atoms into polymethoxy-*trans*-stilbene molecules allowed us to find a compound, 3-bromo-3′,4′,5′,4-tetramethoxy-*trans*-stilbene, with anticancer activity against Jurkat Human acute T-cell leukaemia cells similar to that of the well-known and documented reference compound DMU-212 (compound **7**) [43], but with a better therapeutic index.

In addition to the molecular targets for polymethoxy *trans*-stilbenes presented in the literature, such as cytochromes from the P450 family, the predictive model proposed new potential targets, including beta-1 chain tubulin and beta-3 chain tubulin. In the study, compounds **4**, **7**, and **8** exhibited low DNA-binding activity, and in parallel, they induced chromatin condensation and DNA fragmentation in Jurkat cells, suggesting pro-apoptotic activity.

In the long run, the synthesis of *cis* derivatives of the studied stilbene systems by isomerisation with UV light (approximately 300 nm) or de novo synthesis using the method described by Stefański et al. seems fully justified and provides further scope for exploration of the medicinal chemistry of stilbene derivatives [44].

## Figures and Tables

**Table 1:** The chemical structures of the synthesised and biologically tested compounds **1**–**12**. DMU-212 was assigned the number **7** according to the code used in the table below.

Compound	Substituents							Yield [%]
	Ring B (Position in Ring)				Ring A (Position in Ring)			
	R_1_ (Pos.2)	R_2_ (Pos.3)	R_3_ (Pos.4)	R_4_ (Pos.5)	R^1^ (Pos.2)	R^2^ (Pos.3)	R^3^ (Pos.2/6)	
**1**	-H	-H	-Br	-H	-H	-OCH_3_	-H	55
**2**	-H	-Br	-OCH_3_	-OCH_3_	-H	-OCH_3_	-H	56
**3**	-H	-Br	-OCH_3_	-Br	-H	-OCH_3_	-H	54
**4**	-H	-Br	-OCH_3_	-H	-H	-OCH_3_	-H	59
**5**	-Br	-H	-OCH_3_	-OCH_3_	-H	-OCH_3_	-H	63
**6**	-H	-Br	-H	-Br	-H	-OCH_3_	-H	19
**7**	-H	-H	-OCH_3_	-H	-H	-OCH_3_	-H	53 *
**8**	-H	-OCH_3_	-OCH_3_	-H	-H	-OCH_3_	-H	44 *
**9**	-H	-OCH_3_	-OCH_3_	-OCH_3_	-H	-OCH_3_	-H	45 *
**10**	-H	-OCH_3_	-OCH_3_	-H	-OCH_3_	-H	-H	53 *
**11**	-H	-OCH_3_	-OCH_3_	-H	-H	-H	-OCH_3_	40 *
**12**	-H	-OCH_3_	-H	-OCH_3_	-H	-OCH_3_	-H	57 *

* Yield according to previous work [27].

**Table 2:** IC_50_ values of the studied compounds based on MTT test data for 72 h of action.

Compound	Tumour Cell Lines [µM]			Pseudo-Normal Cell Lines [µM]	
	Jurkat Human Acute T-Cell Leukaemia	U251 Human Malignant Glioblastoma	HT-29 Human Colorectal Carcinoma	Balb-3T3 Mice Fibro- Blast	J744.2 Mice Macrophage-Like Cells
**1**	2.26	>100	>100	3.20	6.63
**2**	>100	>100	>100	72.79	7.93
**3**	>100	>100	>100	76.36	20.39
**4**	**0.76**	>100	>100	2.85	5.21
**5**	68.55	>100	>100	51.74	71.13
**6**	65.34	>100	>100	3.75	9.97
**7**	**0.65**	>100	>100	2.79	**0.83**
**8**	**0.82**	>100	>100	5.04	3.83
**9**	>100	>100	>100	>100	84.83
**10**	8.95	>100	>100	>100	91.63
**11**	>100	>100	>100	>100	>100
**12**	67.61	>100	>100	>100	>100

Cytotoxicity below 1 µM is in bold and green for tumour and red for pseudo-normal cells.

**Table 3:** Crystal data and structure refinement for 3,5-dibromo-3′,4′,5′,4-tetramethoxy-*trans*-stilbene **3**.

CCDC Number	2499084
Empirical formula	C_18_H_18_Br_2_O_4_
Formula weight	458.14
Temperature [K]	100
Crystal system	monoclinic
Space group (number)	$P2_{1}\;$(4)
*a* [Å]	4.2837(5)
*b* [Å]	12.3535(14)
*c* [Å]	16.562(2)
α [°]	90
β [°]	96.420(6)
γ [°]	90
Volume [Å^3^]	870.95(18)
*Z*	2.0
*ρ*_calc_ [g/cm^3^]	1.747
*μ* [mm^−1^]	4.673
*F*(000)	456
Crystal size [mm^3^]	0.037 × 0.044 × 0.269
Crystal colour	colourless
Crystal shape	post
Radiation	Mo *K_α_* (λ = 0.71073 Å)
2θ range [°]	4.12 to 50.88 (0.83 Å)
Index ranges	−5 ≤ h ≤ 5 −14 ≤ k ≤ 14 −19 ≤ l ≤ 19
Reflections collected	12,582
Independent reflections	3123 *R*_int_ = 0.0232 *R*_sigma_ = 0.0233
Completeness to θ = 25.242°	99.7
Data/Restraints/Parameters	3123/1/221
Goodness-of-fit on *F*^2^	1.125
Final *R* indexes [*I* ≥ 2σ(*I*)]	*R*_1_ = 0.0455 w*R*_2_ = 0.1002
Final *R* indexes [all data]	*R*_1_ = 0.0496 w*R*_2_ = 0.1035
Largest peak/hole [eÅ^−3^]	0.65/−0.56
Flack X parameter	0.480(3)

## Data Availability

Data are contained within the article and Supplementary Materials.

## Supplementary Materials

The following supporting information can be downloaded at: https://www.mdpi.com/article/10.3390/molecules30244788/s1.
