# Supplementary material for: Bromo Analogues of Active 3,4,5,4′-Tetramethoxy-trans-stilbene (DMU-212)—A New Path of Research to Anticancer Agents
Source: Molecules. 2025 Dec 15;30(24):4788. doi: 10.3390/molecules30244788 (PMC12735739; doi:10.3390/molecules30244788)
Supplement: Supplementary file 1 [file molecules-30-04788-s001.zip › molecules-3989948-supplementary.pdf]

Bromo analogues of active 3,4,5,4′-tetramethoxy-*trans*-stilbene (DMU-212) - a new path of research to anticancer agents

Dawid Łażewski [1], Gabriela Korzańska [1], Łukasz Popenda [2], Karolina Chmaj-Wierzchowska [3], Artur Korzański [4], Eduard Potapskyi [1], Julian Myszkiewicz [1], Agnieszka Gielara-Korzańska [1], Agnieszka Zgoła-Grześkowiak [5],
Nataliya Finiuk [6,7], Yuliia Kozak [6,7], Iryna Ivasechko [6,7], Roman Lesyk [7,8,9], Joanna Kuźmińska [10], Tomasz Goslinski [1] and Marcin Wierzchowski [1,*]

1 Poznan University of Medical Sciences, Department of Chemical Technology of Drugs, Grunwaldzka 6 Street, 60-780 Poznan, Poland; lazewskidawid@gmail.com (D.Ł.); gabrysia.korzanska@gmail.com (G.K.); potapskiyed@gmail.com (E.P.); julikmyszk@gmail.com (J.M.); agnieszka.gielara@op.pl (A.G.-K.); tomasz.goslinski@ump.edu.pl (T.G.)
2 Adam Mickiewicz University, NanoBioMedical Centre, Wszechnicy Piastowskiej 3 Street, 61-614 Poznan, Poland; lukasz.popenda@amu.edu.pl
3 Poznan University of Medical Sciences, Department of Maternal and Child Health, 60-701 Poznan, Poland; karolinachmaj@poczta.onet.pl
4 Adam Mickiewicz University, Department of Chemistry, Uniwersytetu Poznanskiego 8 Street, 61-614 Poznan, Poland; artur.korzanski@amu.edu.pl
5 Poznan University of Technology, Institute of Chemistry and Technical Electrochemistry, Berdychowo 4, 60-965 Poznan, Poland; agnieszka.zgola-grzeskowiak@put.poznan.pl
6 Institute of Cell Biology of the National Academy of Sciences of Ukraine, Department of Regulation of Cell Proliferation and Apoptosis, Drahomanov Street 14/16, 79005 Lviv, Ukraine; nataliyafiniuk@gmail.com (N.F.); juliana.kozzak@gmail.com (Y.K.); irynagrytsyna@gmail.com (I.I.)
7 Danylo Halytsky Lviv National Medical University, Molecular Design Center, Pekarska 69, 79010 Lviv, Ukraine; roman.lesyk@gmail.com
8 Danylo Halytsky Lviv National Medical University, Department of Pharmaceutical, Organic and Bioorganic Chemistry, 79010 Lviv, Ukraine
9 University of Information Technology and Management in Rzeszow, Department of Biotechnology and Cell Biology, Medical College, Sucharskiego 2, 35-225 Rzeszow, Poland
10 Poznan University of Medical Sciences, Department of Pharmaceutical Chemistry, Rokietnicka 3, 60-806 Poznan, Poland; jkuzminska@ump.edu.pl
* Correspondence: mwierzch@ump.edu.pl

Supporting Information

Table of contents

1. NMR data 3
   1.1. NMR experiments of 4-bromo-3′,4′, 5′-tri methoxy-*trans*-stilbene (**1**). 3
   1.2. NMR experiments of 4-bromo-4,5,3′,4′, 5′-penta methoxy-*trans*-stilbene (**2**).................... 6
   1.3. NMR experiments of 3,5-dibromo-4,3′,4′, 5′-twtra methoxy-*trans*-stilbene (**3**). 9
   1.4. NMR experiments of 3-bromo-4,3′,4′, 5′-tetra methoxy-*trans*-stilbene (**4**). 12
   1.5. NMR experiments of 2-bromo-3,4,3′,4′, 5′-penta methoxy-*trans*-stilbene (**5**).................... 15
   1.6. NMR experiments of 3,5-dibromo-3′,4′, 5′-tri methoxy-*trans*-stilbene (**6**). 18
2. Mass spectrometry data....................21
   2.1. ESI mass spectra and $[M+H]^+$ ion fragmentation of stilbene **1** 21

2.2. ESI mass spectra and $[M+H]^+$ ion fragmentation of stilbene **2** 23
2.3. ESI mass spectra and $[M+H]^+$ ion fragmentation of stilbene **3** 25
2.4. ESI mass spectra and $[M+H]^+$ ion fragmentation of stilbene **4** 28
2.5. ESI mass spectra and $[M+H]^+$ ion fragmentation of stilbene **5** 30
2.6. ESI mass spectra and $[M+H]^+$ ion fragmentation of stilbene **6** 32
3. UV-vis spectra 34
4. Crystallographic data 36
Table S1. Atomic coordinates and Ueq [Å^2] for **3**. 36
Table S2. Anisotropic displacement parameters (Å^2) for mw241122. The anisotropic displacement factor exponent takes the form: $-2\pi^2[\, h^2(a^*)^2U_{11} + k^2(b^*)^2U_{22} + \ldots + 2hka^*b^*U_{12}\,]$ 37
Table S3. Bond lengths and angles for **3.** 39
Table S4. Torsion angles for **3**. 42
5. Computational data 43
6. HPLC data 52

1. NMR data

* denotes solvent signal

~ denotes water signal

1.1. NMR experiments of 4-bromo-3′,4′, 5′-tri methoxy-*trans*-stilbene (**1**).

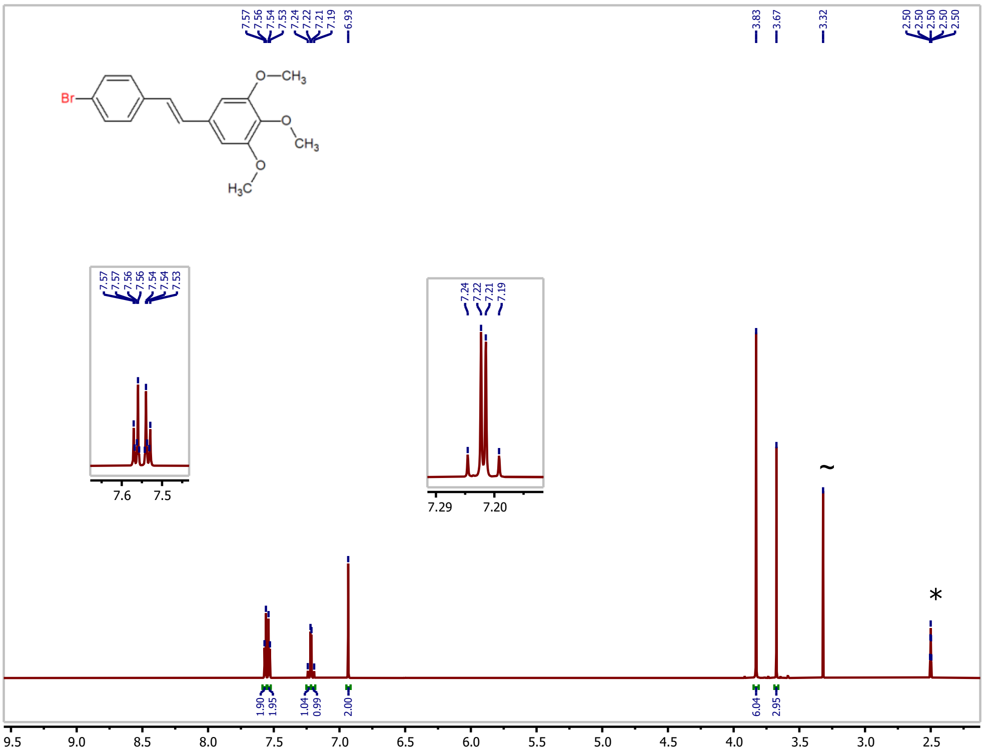

Figure S1. ^{1}H NMR of stilbene **1** (800 MHz, DMSO-d_6, 298K).

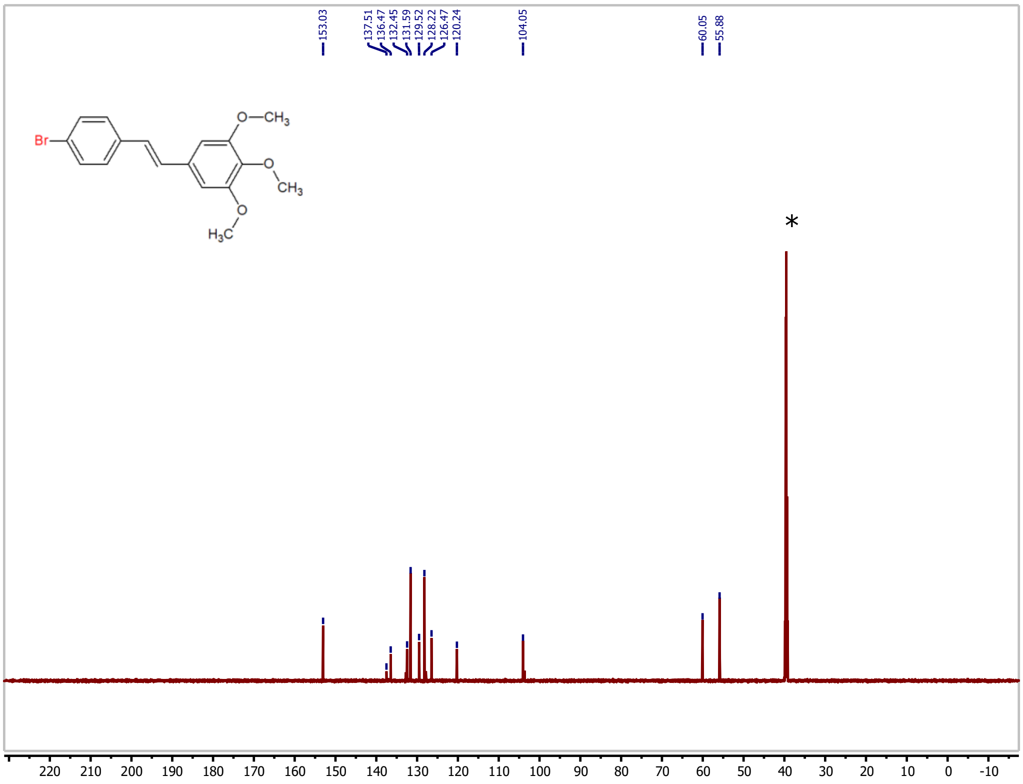

Figure S2. ^{13}C NMR of stilbene **1** (201 MHz, DMSO-d_6, 298K).

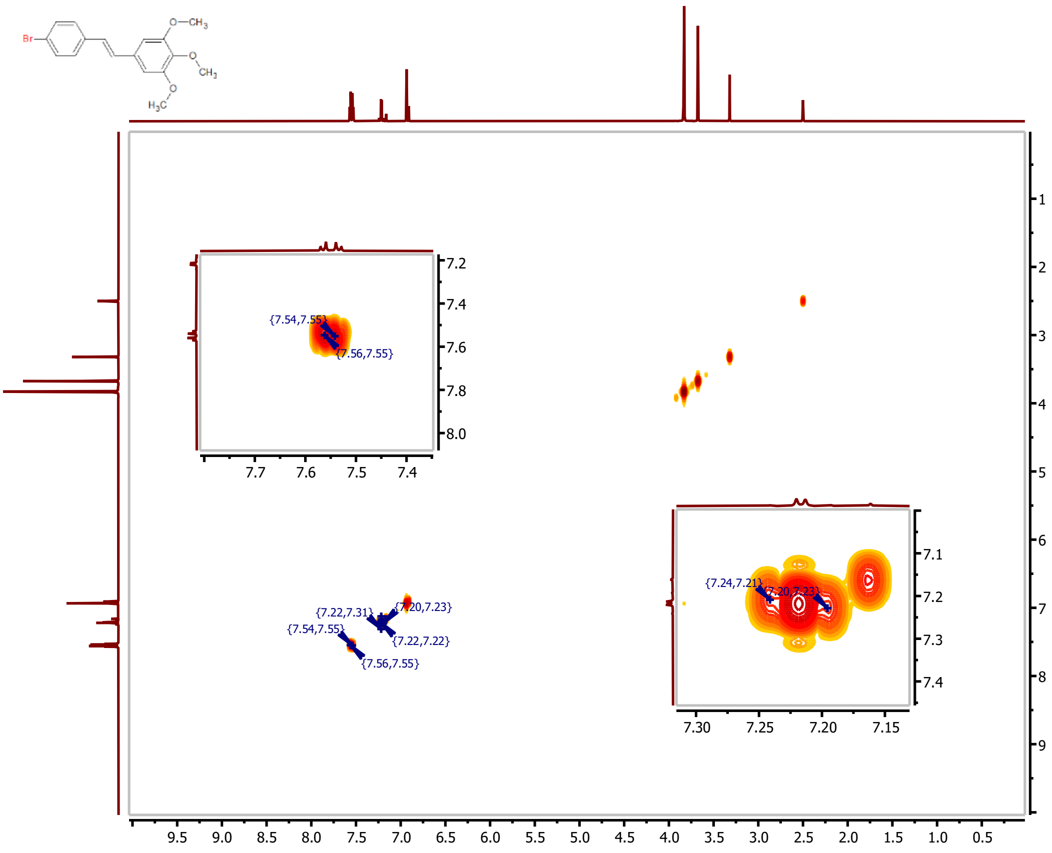

Figure S3. ^{1}H-^{1}H COSY of stilbene **1** (DMSO-d_6, 298K).

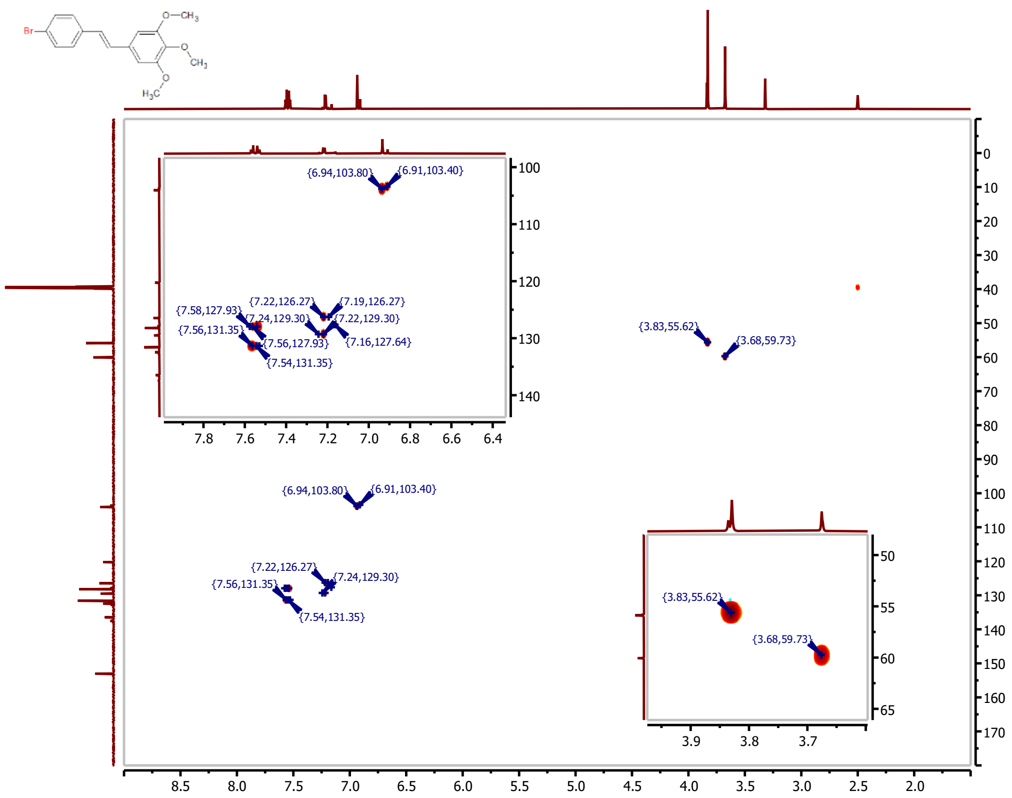

Figure S4. ^{1}H-^{13}C HSQC of stilbene **1** (DMSO-d_6, 298K).

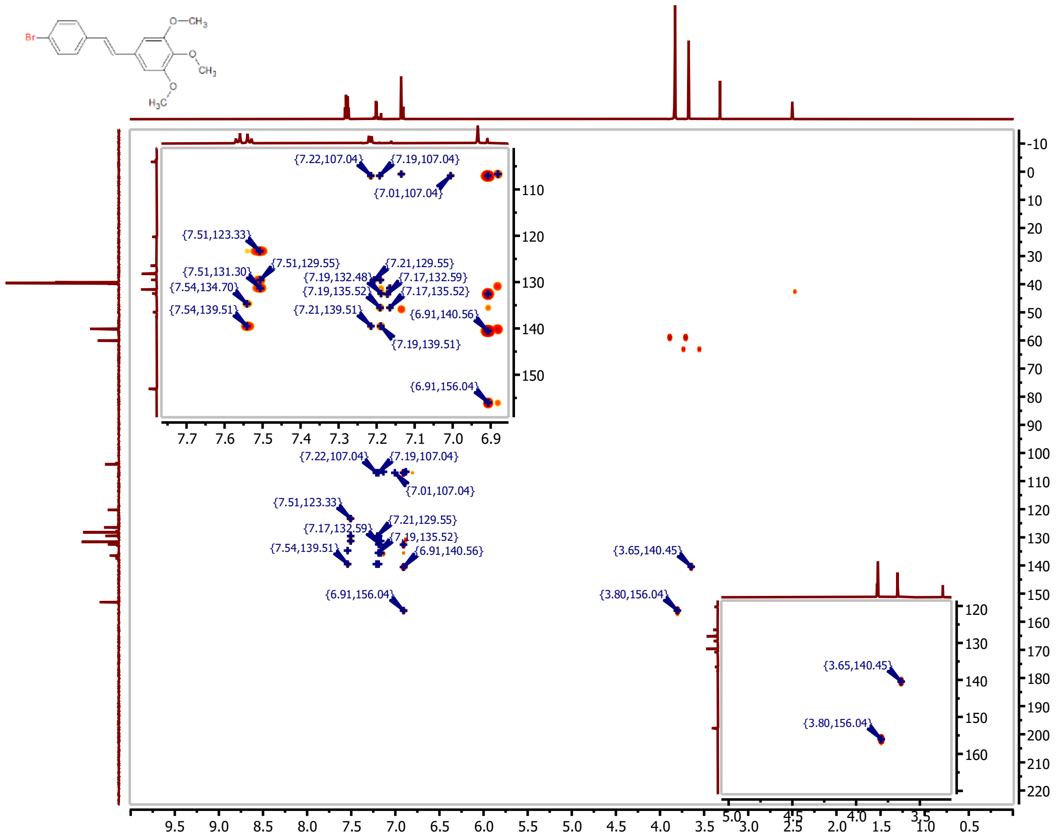

Figure S5. ^{1}H-^{13}C HMBC of stilbene **1** (DMSO-d_6, 298K).

1.2. NMR experiments of 4-bromo-4,5,3′,4′, 5′-penta methoxy-*trans*-stilbene (**2**).

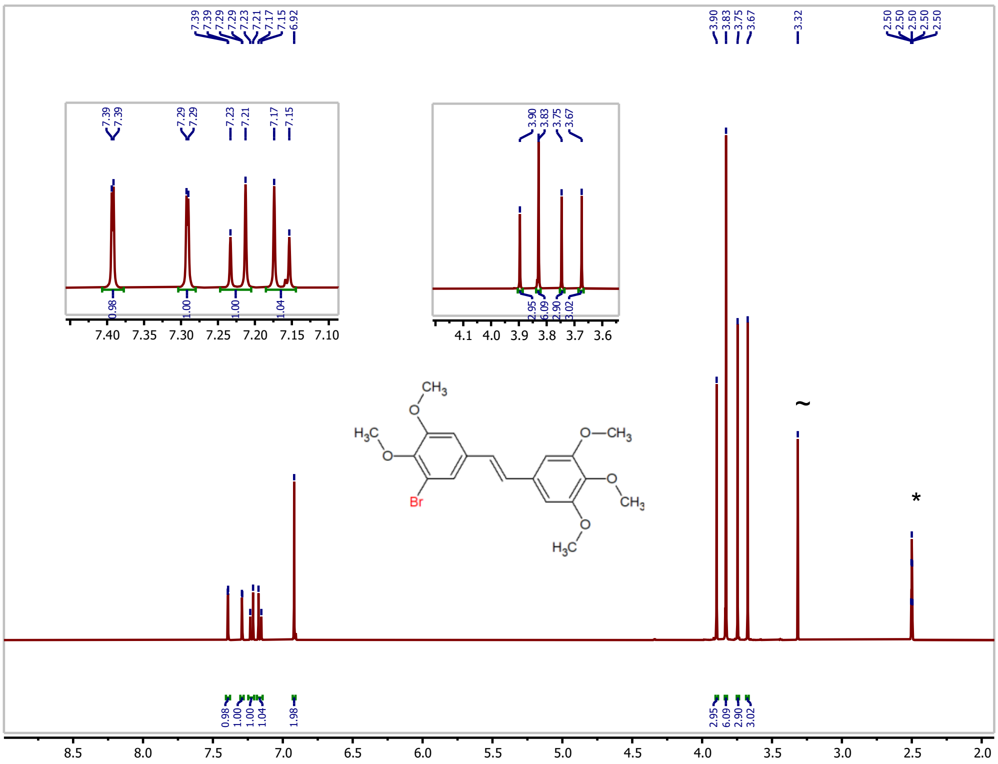

Figure S6. ^{1}H NMR of stilbene **2** (800 MHz, DMSO-d_6, 298K).

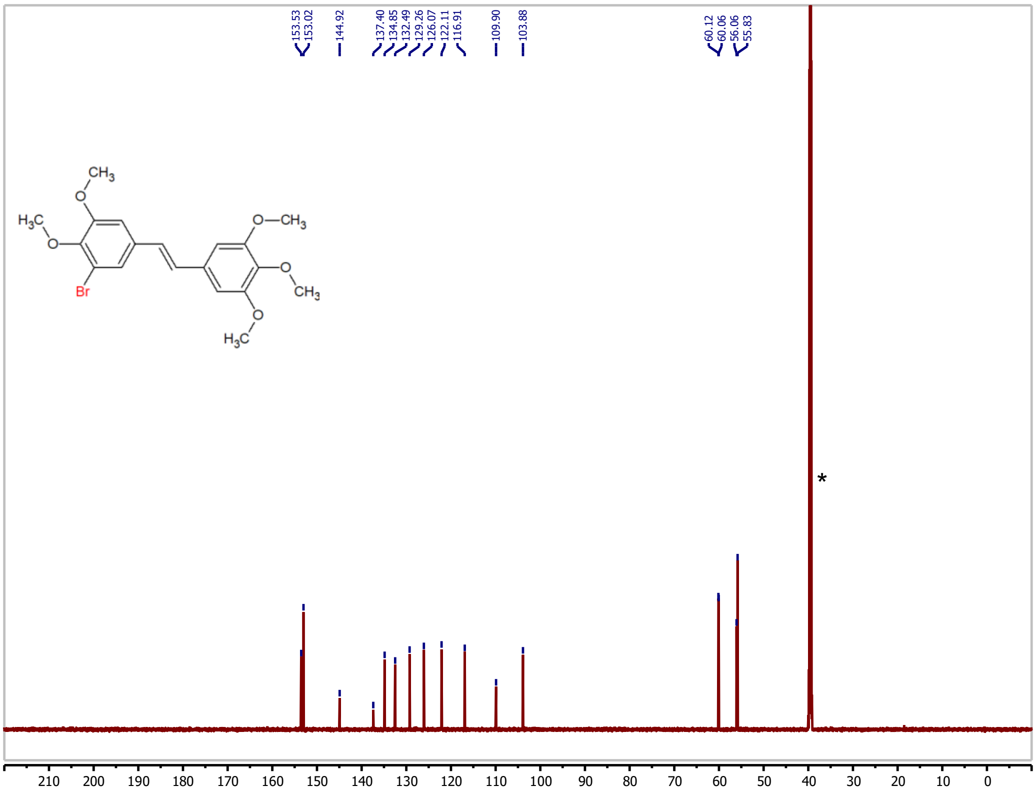

Figure S7. ^{13}C NMR of stilbene **2** (201 MHz, DMSO-d_6, 298K).

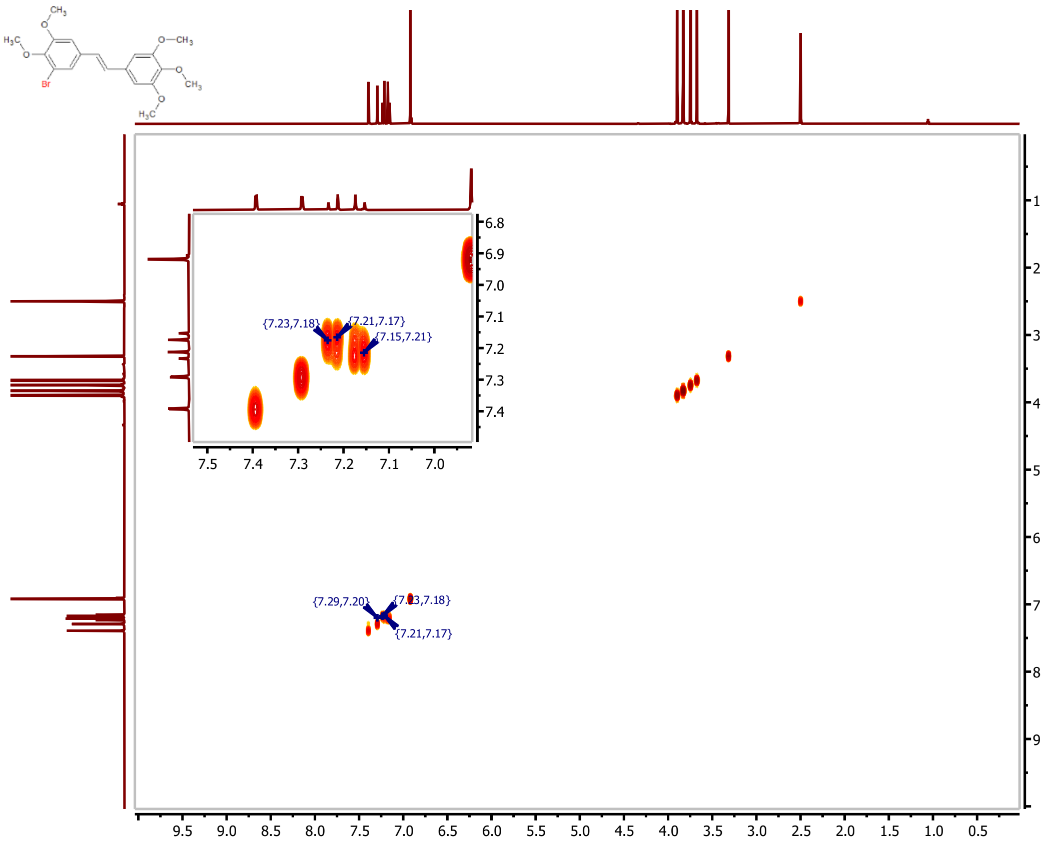

Figure S8. ^{1}H-^{1}H COSY of stilbene **2** (DMSO-*d*$_6$, 298K).

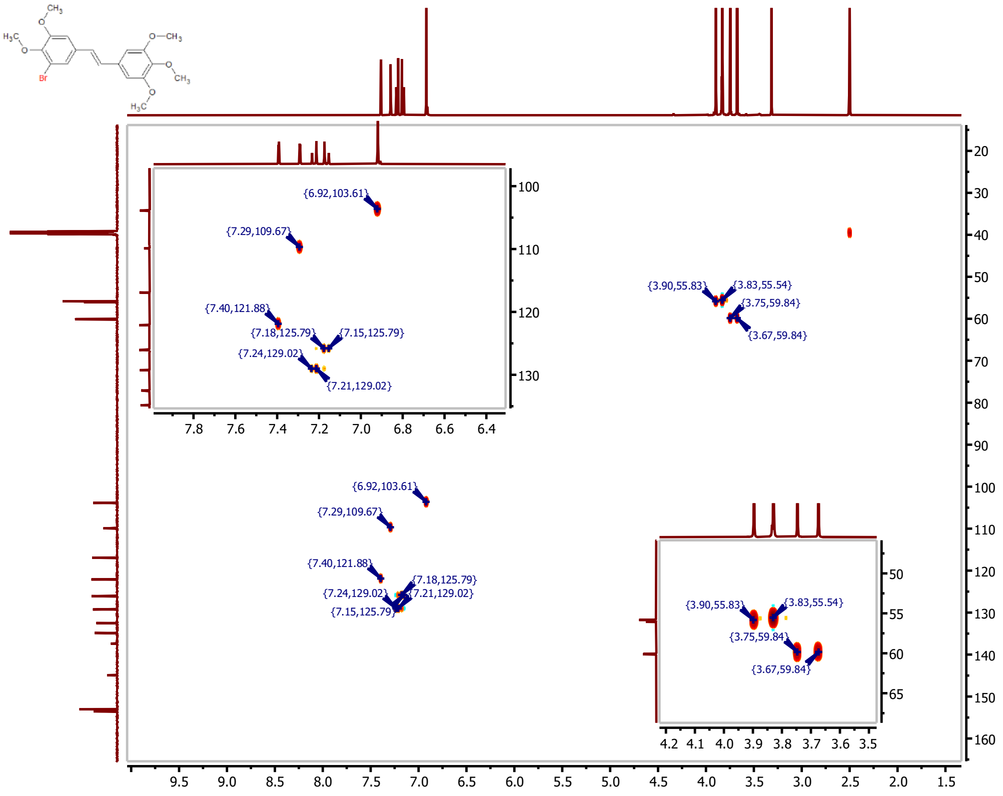

Figure S9. ^{1}H-^{13}C HSQC of stilbene **2** (DMSO-*d*$_6$, 298K).

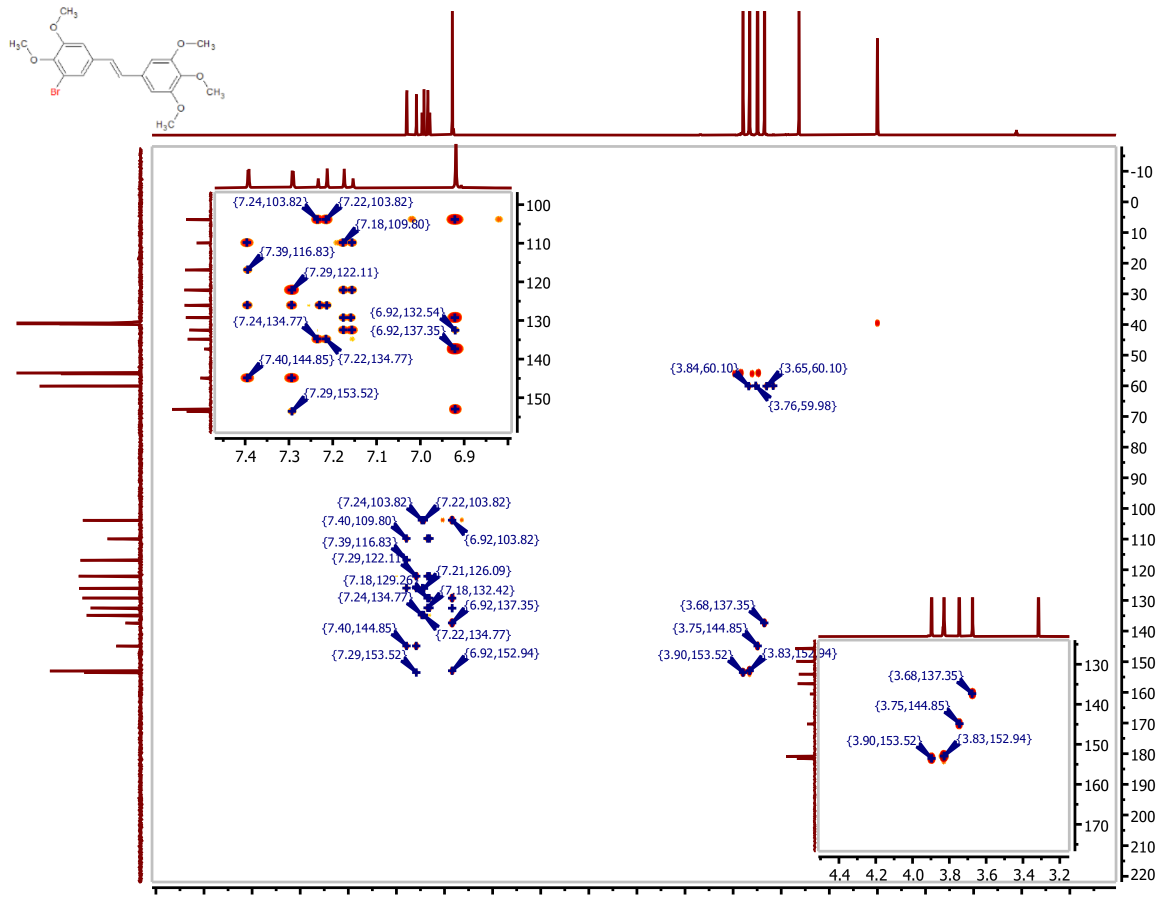

Figure S10. ^1H-^{13}C HMBC of stilbene **2** (800 MHz, 201 MHz, DMSO-d_6)

1.3.NMR experiments of 3,5-dibromo-4,3′,4′, 5′-twtra methoxy-*trans*-stilbene (**3**).

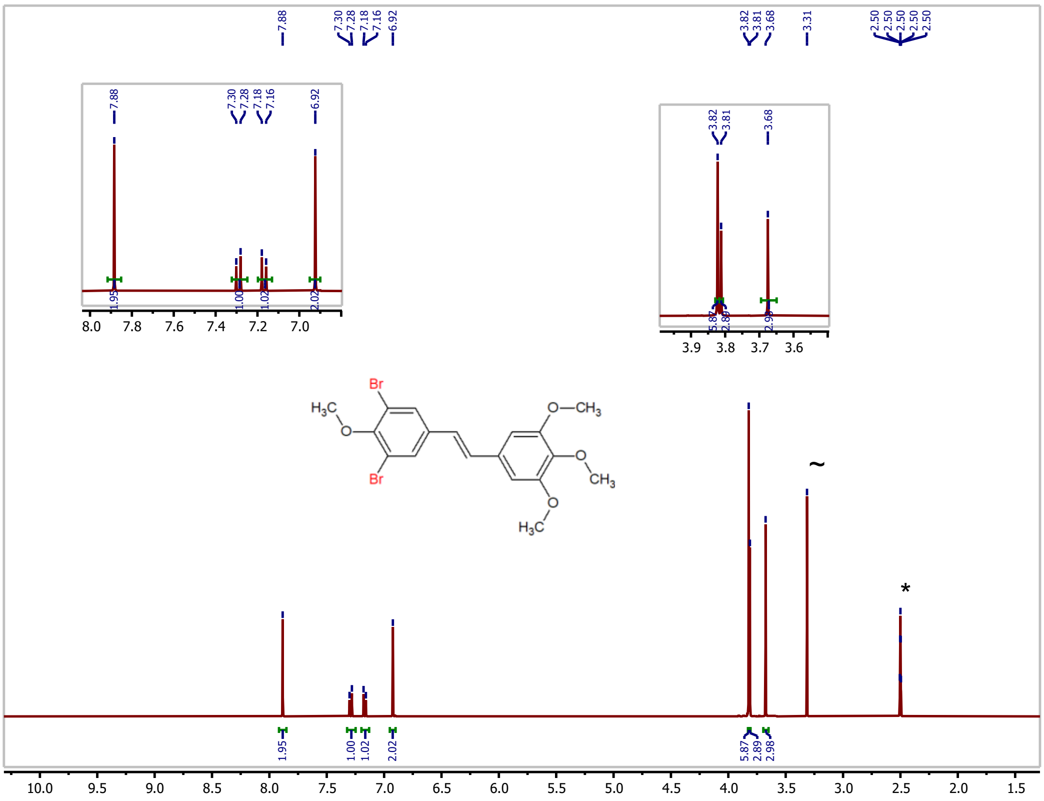

Figure S11. ^{1}H NMR of stilbene **3** (800 MHz, DMSO-d_6, 298K).

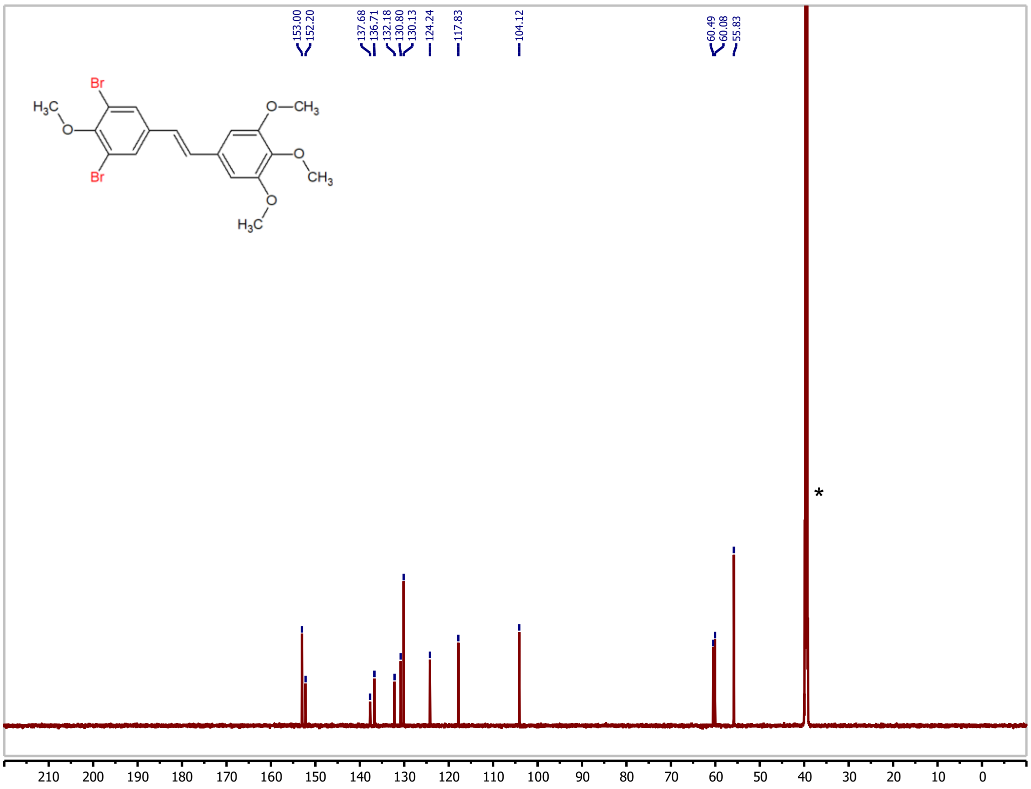

Figure S12. ^{13}C NMR of stilbene **3** (201 MHz, DMSO-d_6, 298K).

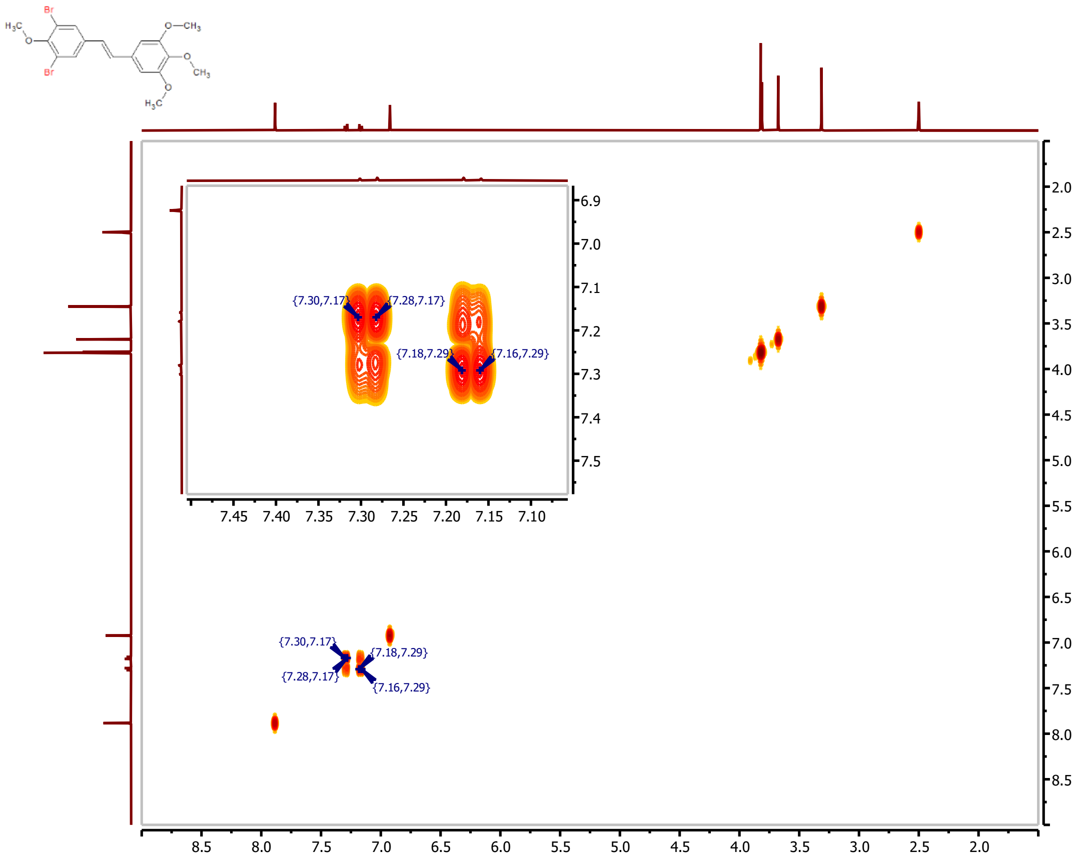

Figure S13. ^{1}H-^{1}H COSY of stilbene **3** (DMSO-d_6, 298K).

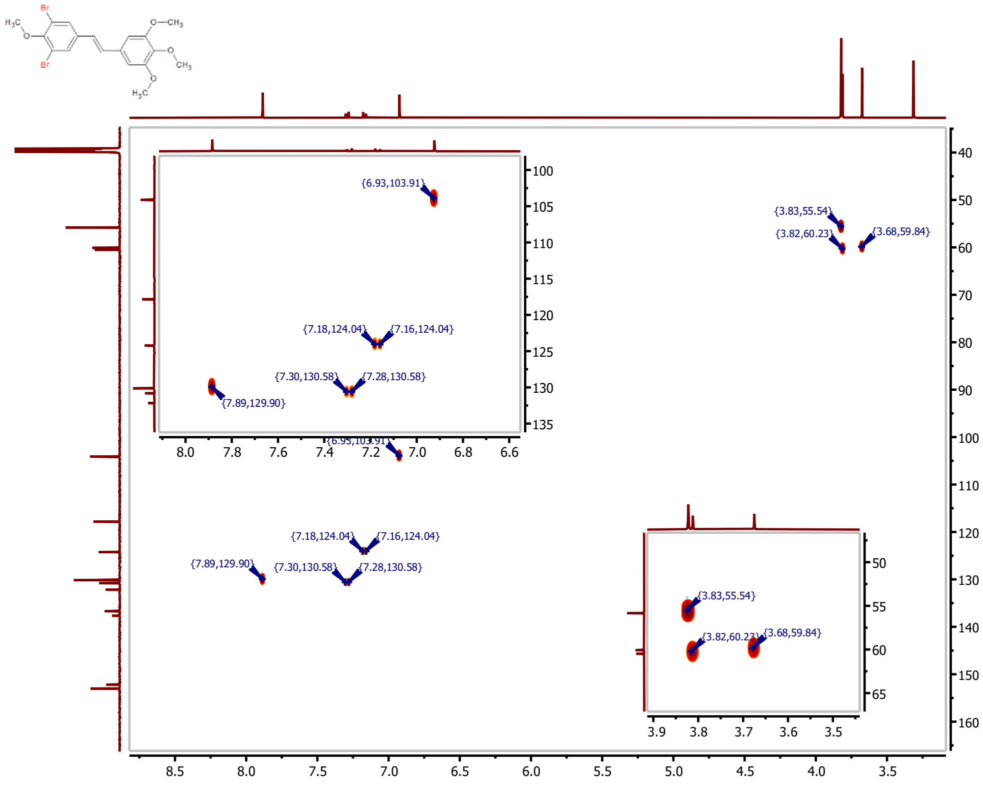

Figure S14. ^{1}H-^{13}C HSQC of stilbene **3** (DMSO-d_6, 298K).

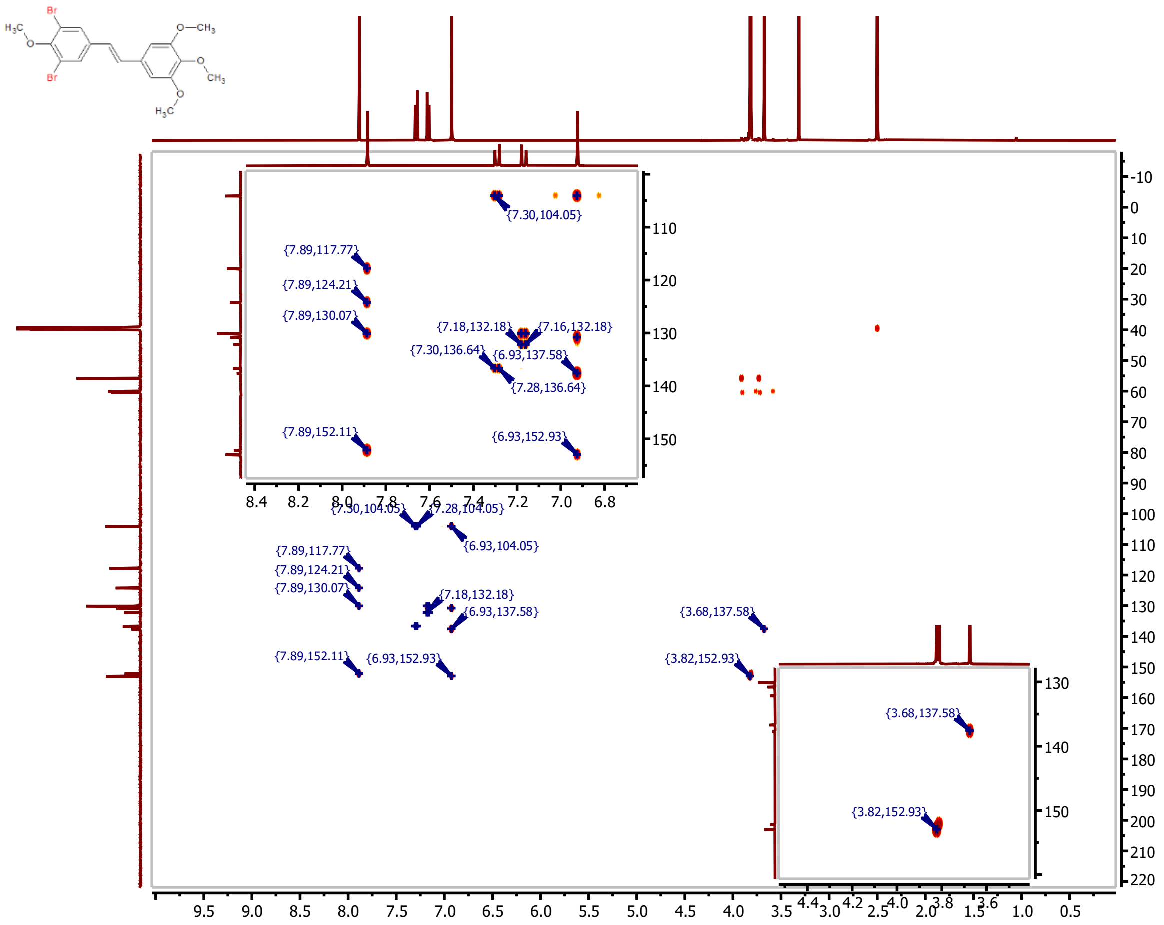

Figure S15. ^{1}H-^{13}C HMBC of stilbene **3** (DMSO-d_6, 298K).

1.4.NMR experiments of 3-bromo-4,3′,4′, 5′-tetra methoxy-*trans*-stilbene (**4**).

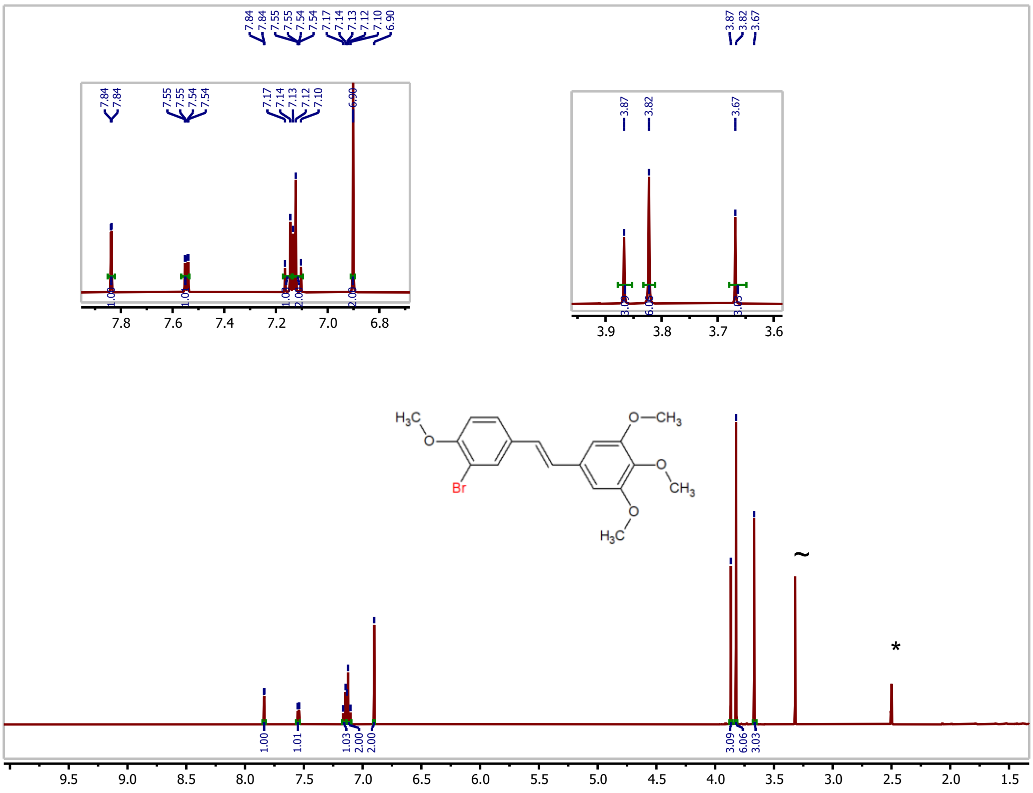

Figure S16. ^{1}H NMR of stilbene **4** (800 MHz, DMSO-d_6, 298K).

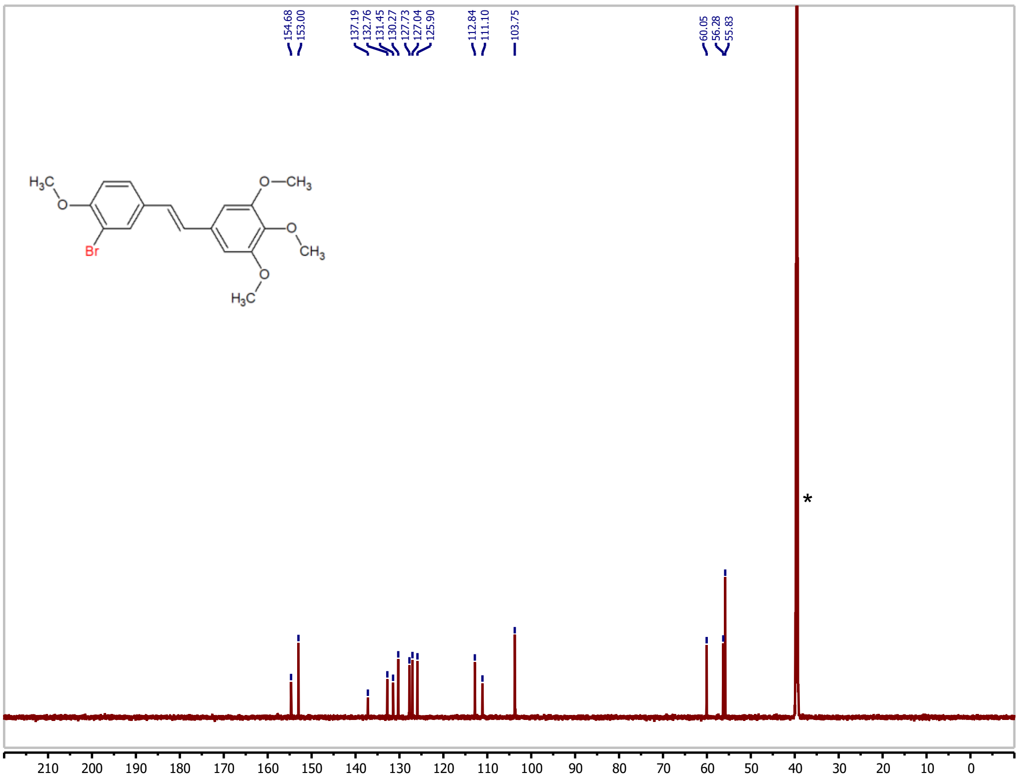

Figure S17. ^{13}C NMR of stilbene **4** (201 MHz, DMSO-d_6, 298K).

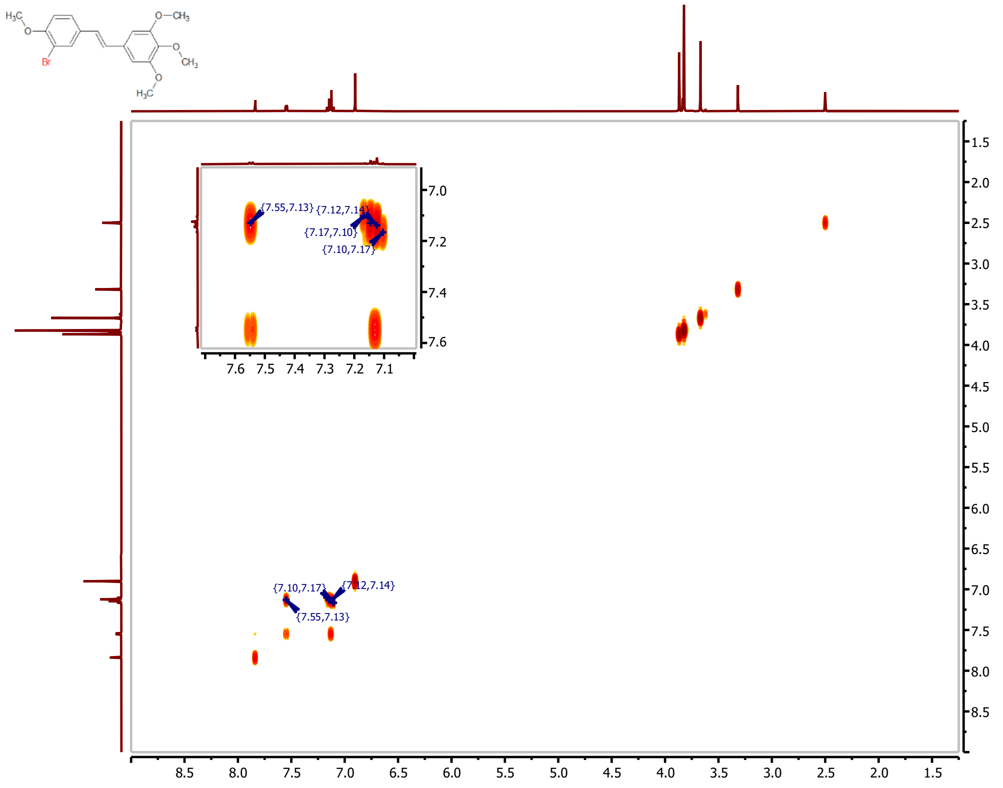

Figure S18. ^{1}H-^{1}H COSY of stilbene **4** (DMSO-d_6, 298K).

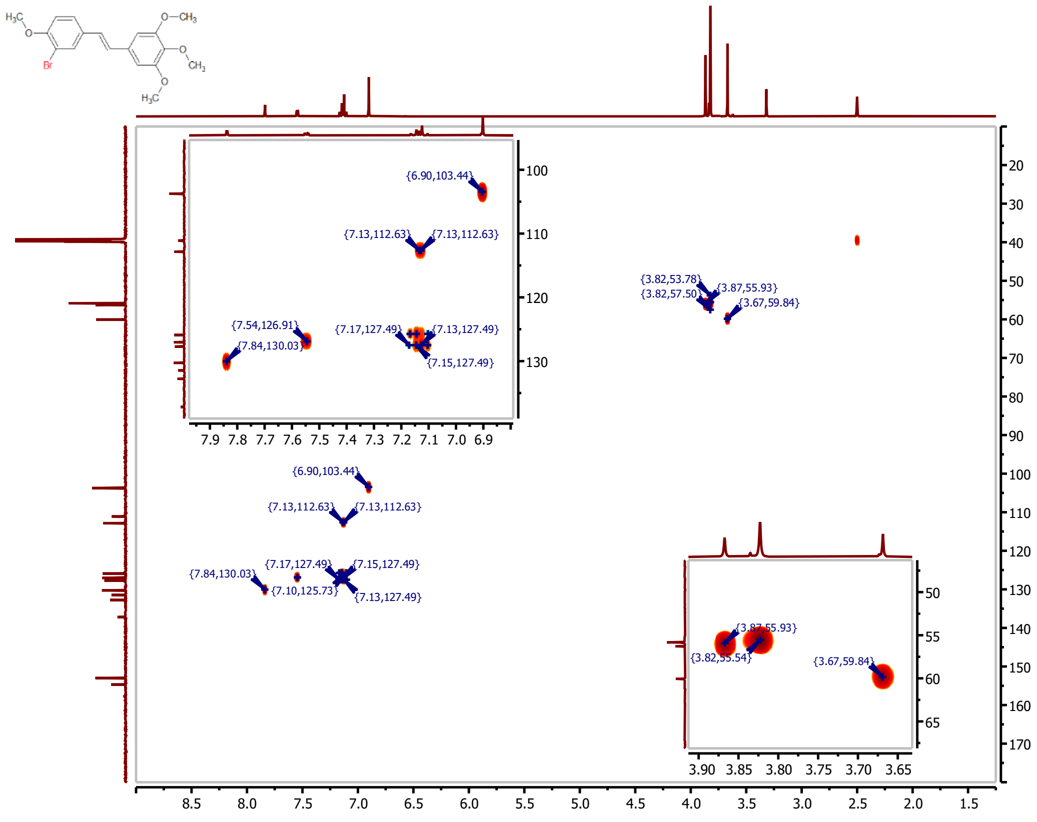

Figure S19. ^{1}H-^{13}C HSQC of stilbene **4** (DMSO-d_6, 298K).

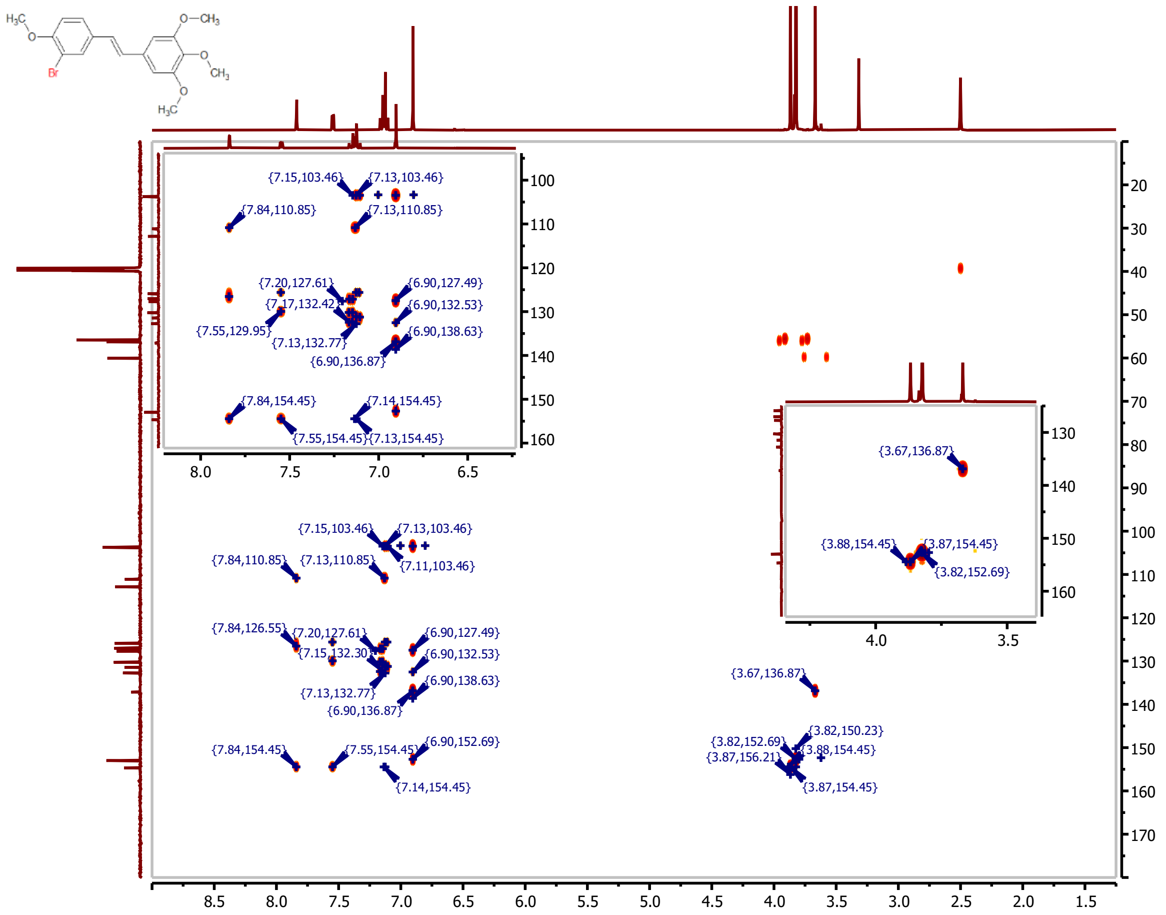

Figure S20. ^{1}H-^{13}C HMBC of stilbene **4** (DMSO-d_6, 298K).

1.5.NMR experiments of 2-bromo-3,4,3′,4′, 5′-penta methoxy-*trans*-stilbene (**5**).

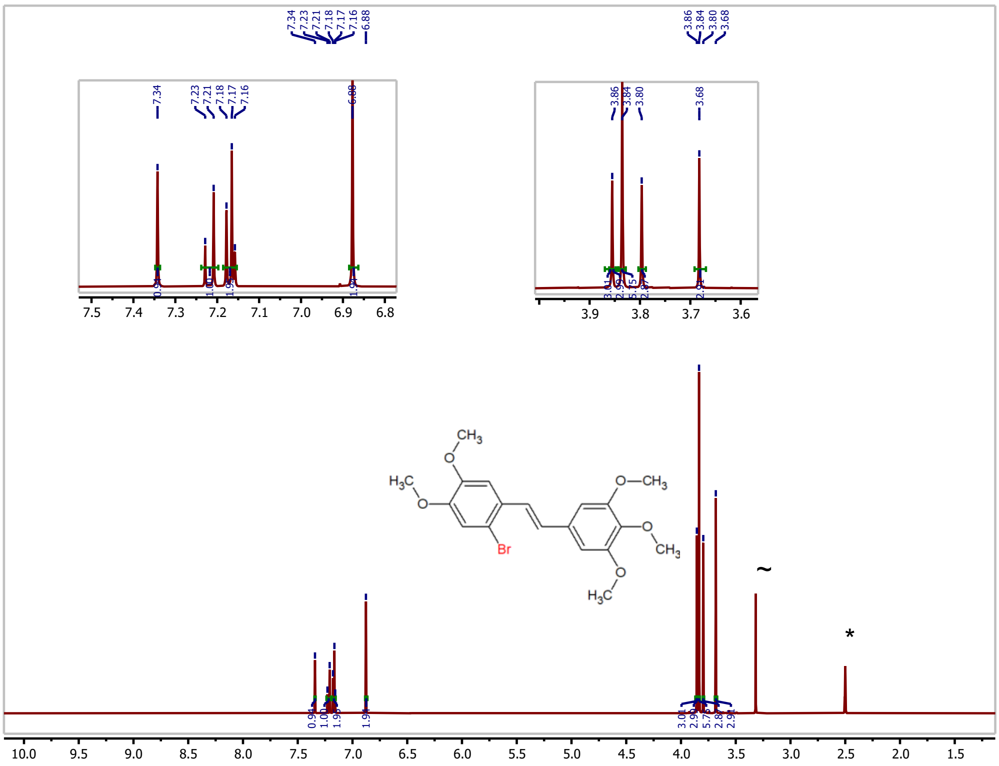

Figure S21. ^{1}H NMR of stilbene **5** (800 MHz, DMSO-d_6, 298K).

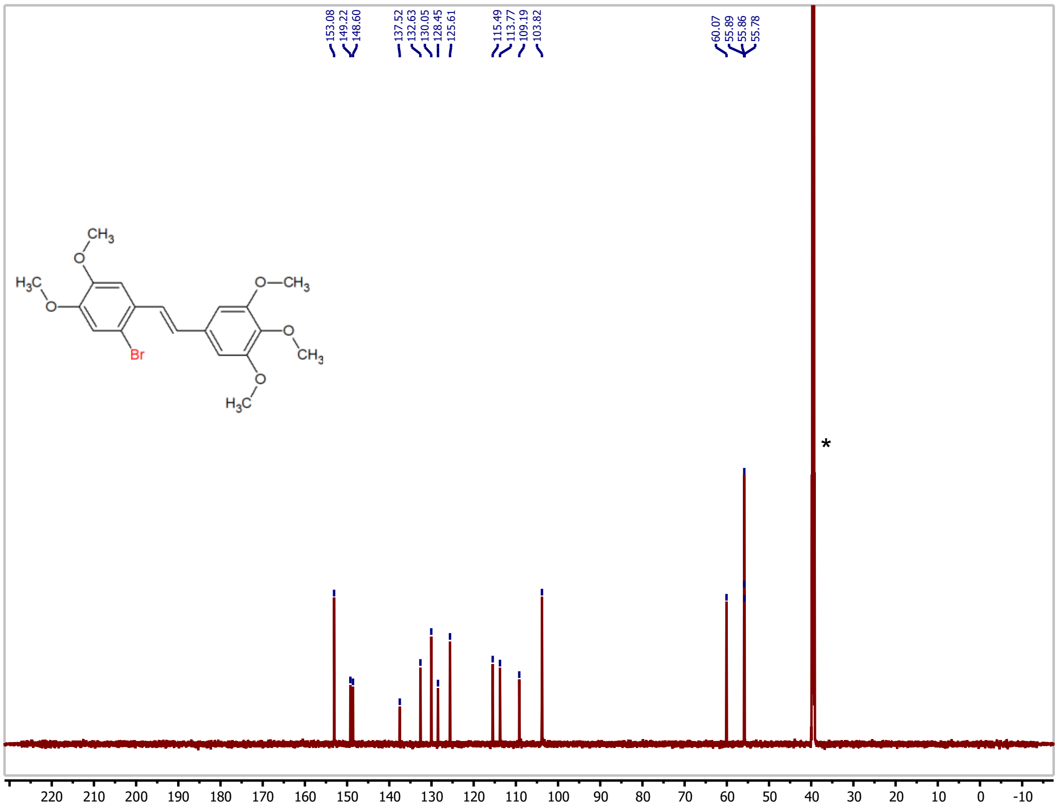

Figure S22. ^{13}C NMR of stilbene **5** (201 MHz, DMSO-d_6, 298K).

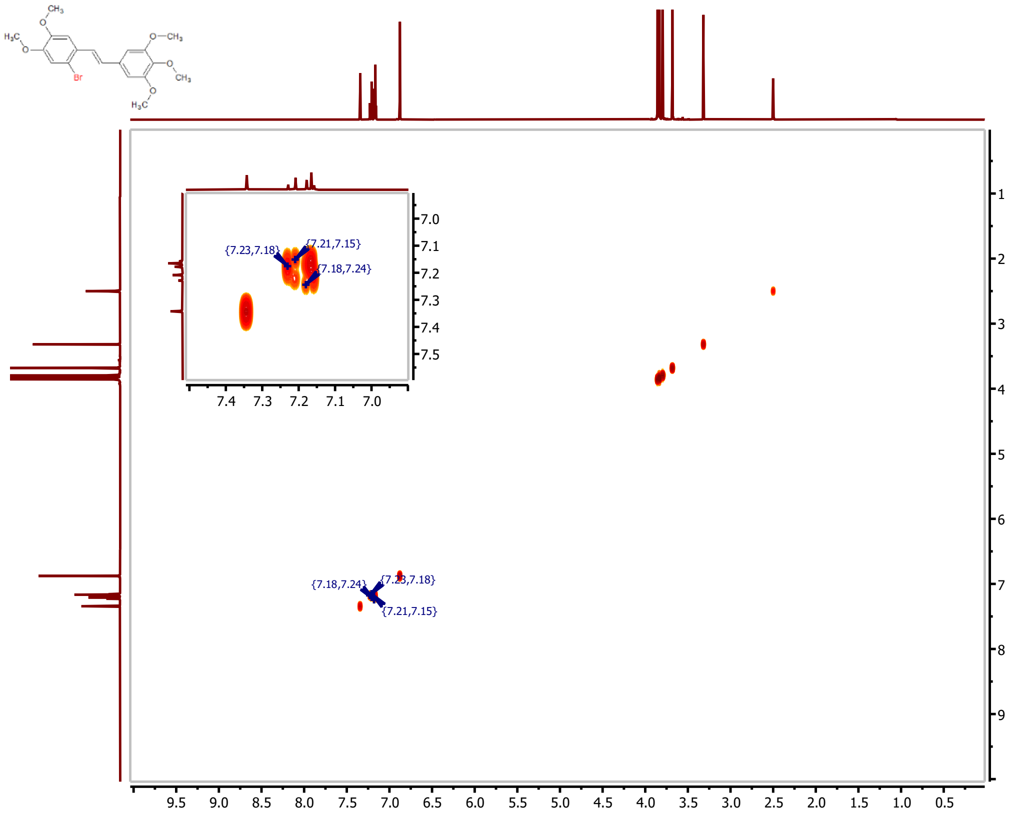

Figure S23. ^{1}H-^{1}H COSY of stilbene **5** (DMSO-*d*$_6$, 298K).

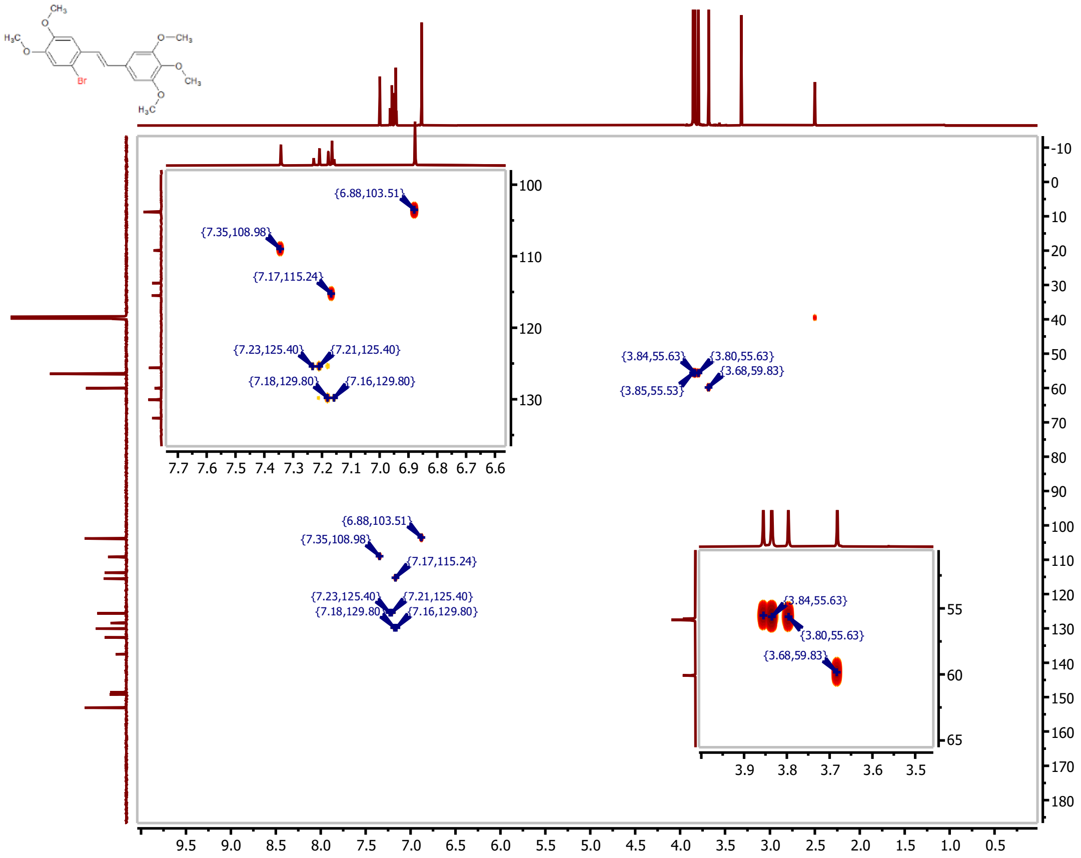

Figure S24. ^{1}H-^{13}C HSQC of stilbene **5** (DMSO-*d*$_6$, 298K).

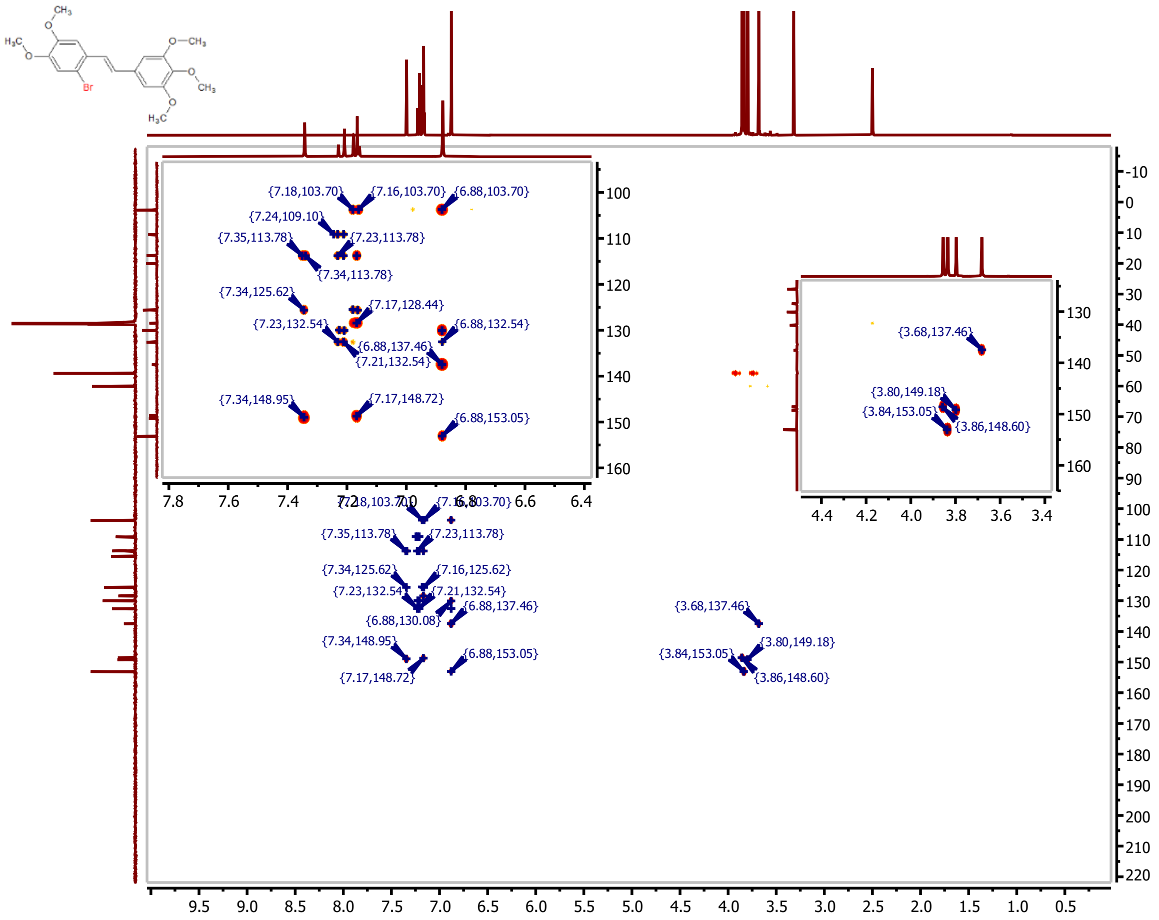

Figure S25. ^{1}H-^{13}C HMBC of stilbene **5** (DMSO-d_6, 298K).

1.6.NMR experiments of 3,5-dibromo-3′,4′, 5′-tri methoxy-*trans*-stilbene (**6**).

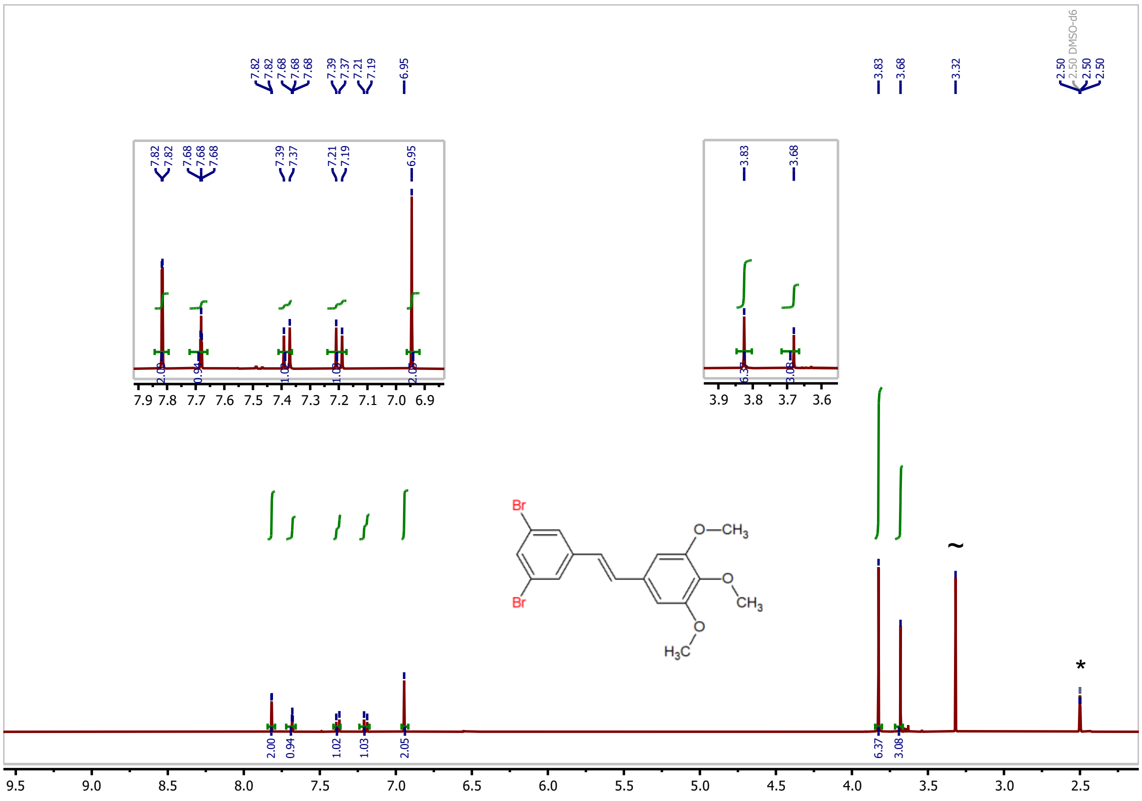

Figure S26. ^{1}H NMR of stilbene **6** (800 MHz, DMSO-d_6, 298K).

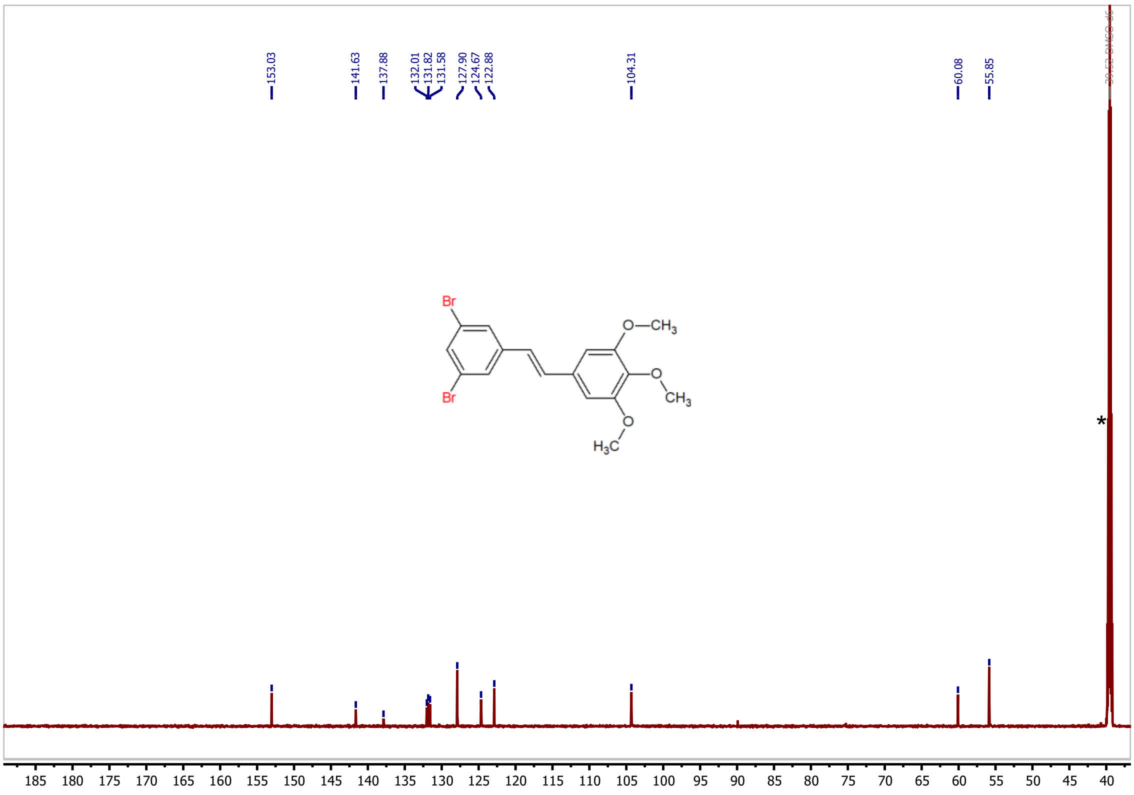

Figure S27. ^{13}C NMR of stilbene **6** (201 MHz, DMSO-d_6, 298K).

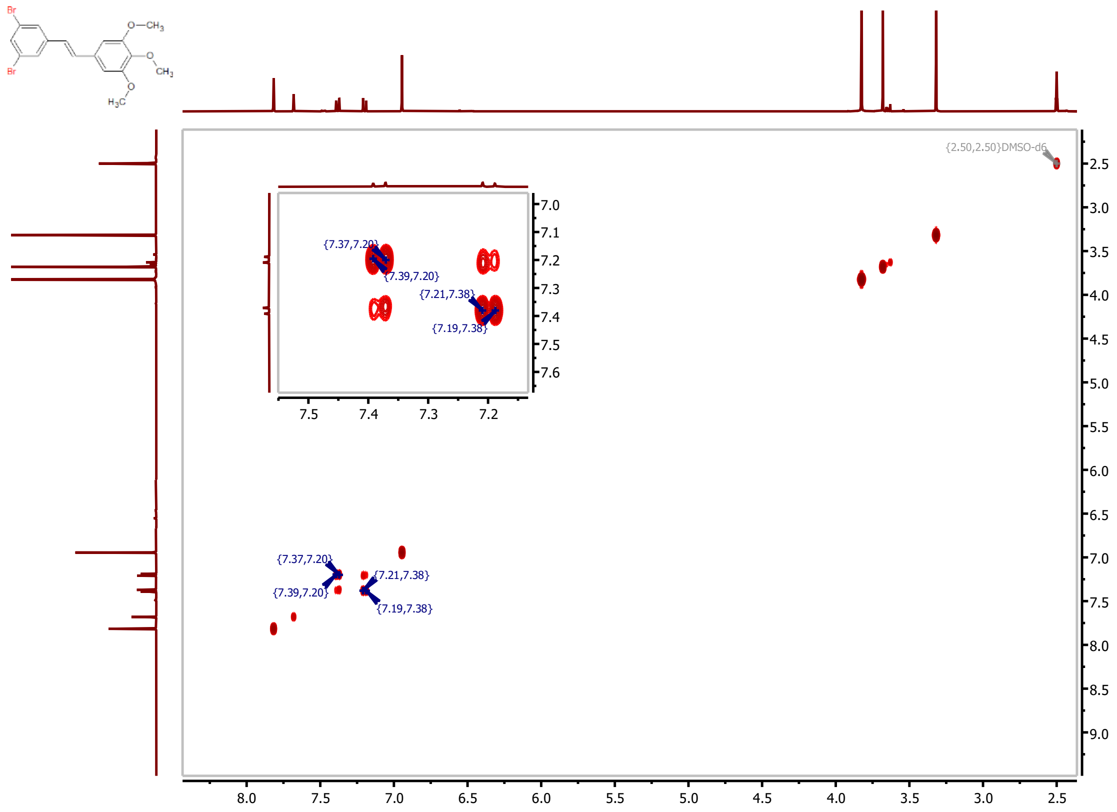

Figure S28. ^{1}H-^{1}H COSY of stilbene **6** (DMSO-*d*$_6$, 298K).

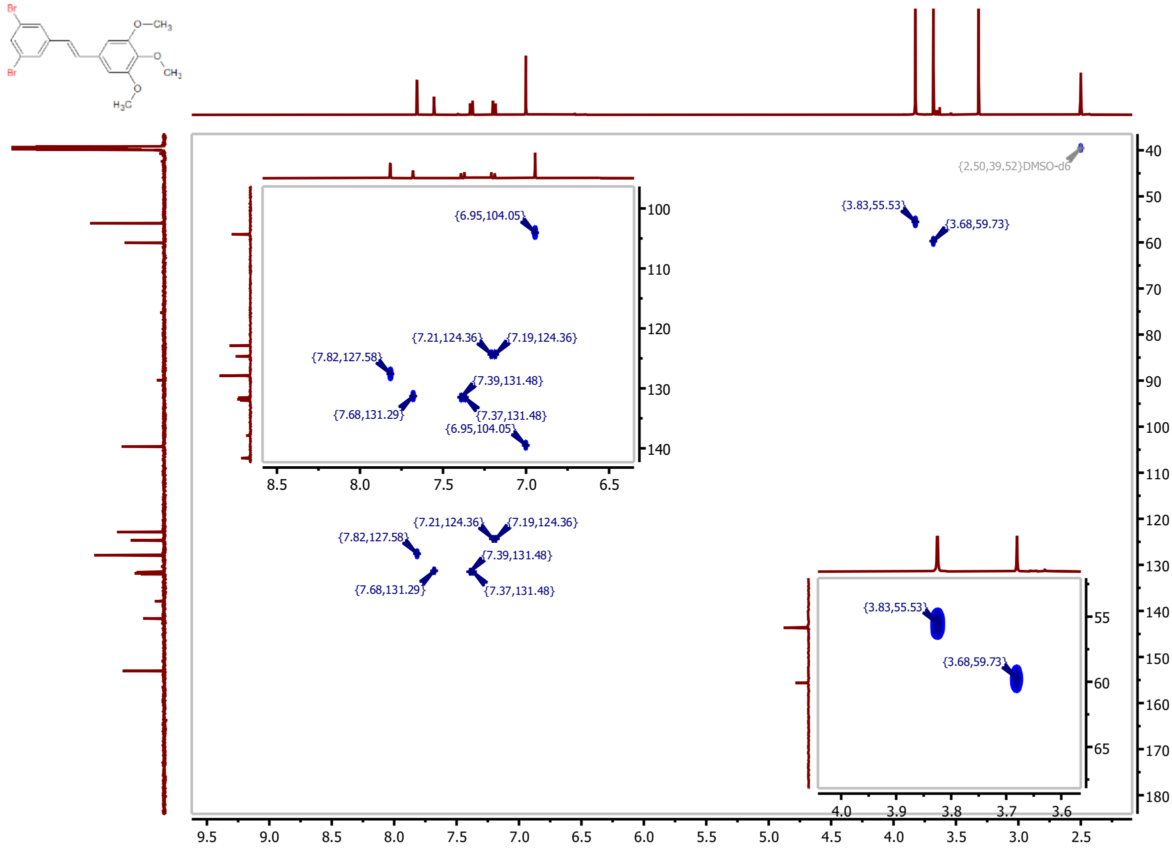

Figure S29. ^{1}H-^{13}C HSQC of stilbene **6** (DMSO-*d*$_6$, 298K).

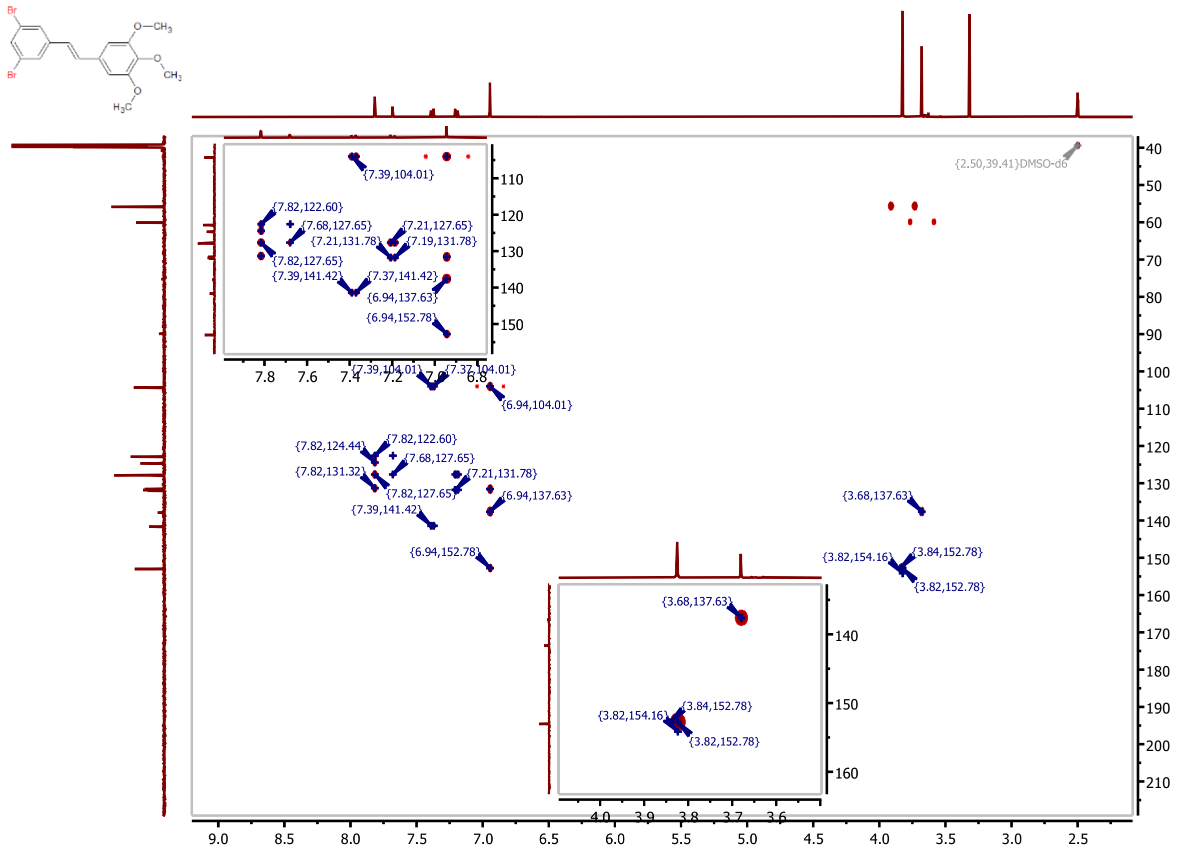

Figure S30. ^{1}H-^{13}C HMBC of stilbene **6** (DMSO-d_6, 298K).

2. Mass spectrometry data

2.1. ESI mass spectra and [M+H]$^+$ ion fragmentation of stilbene **1**

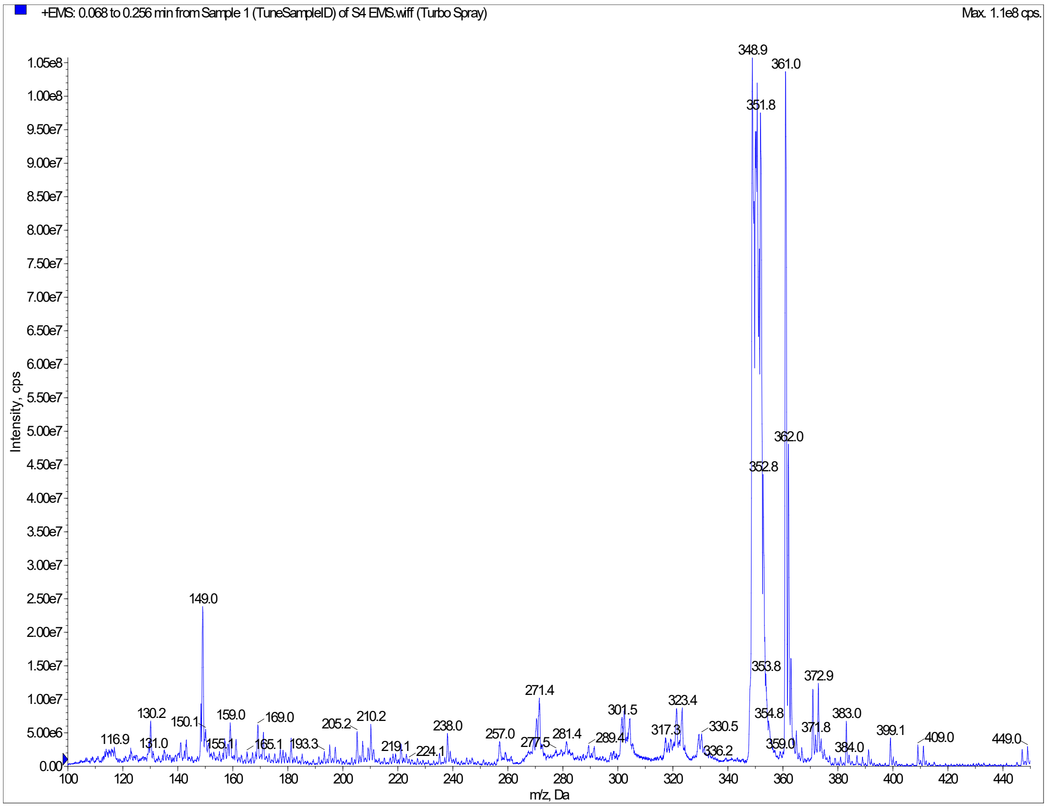

Figure S31. Fullscan MS spectrum of **1** [M+H]$^+$ (^{79}Br) 348.9 m/z, (^{81}Br) 351.8 m/z

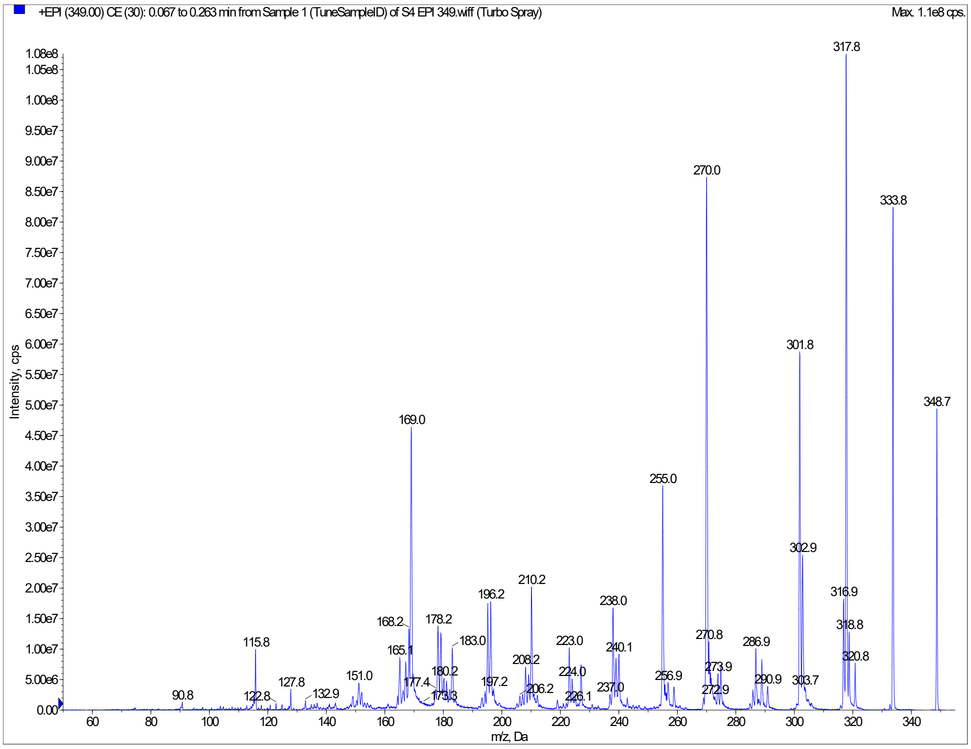

Figure S32. Fragmentation spectrum of **1** [M+H]$^+$ (^{79}Br) 348.9 m/z

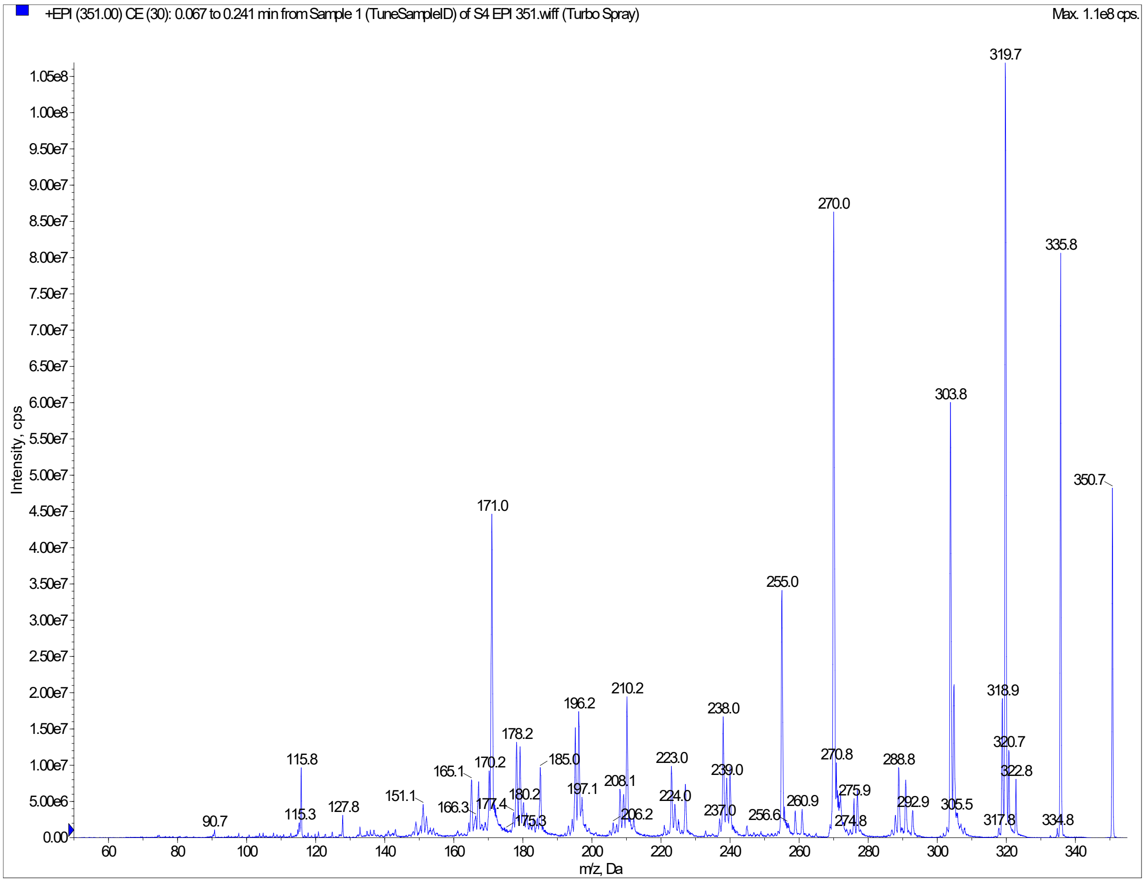

Figure S33. Fragmentation spectrum of **1** [M+H]+ ([81]Br) 351.8 m/z

2.2. ESI mass spectra and [M+H]+ ion fragmentation of stilbene **2**

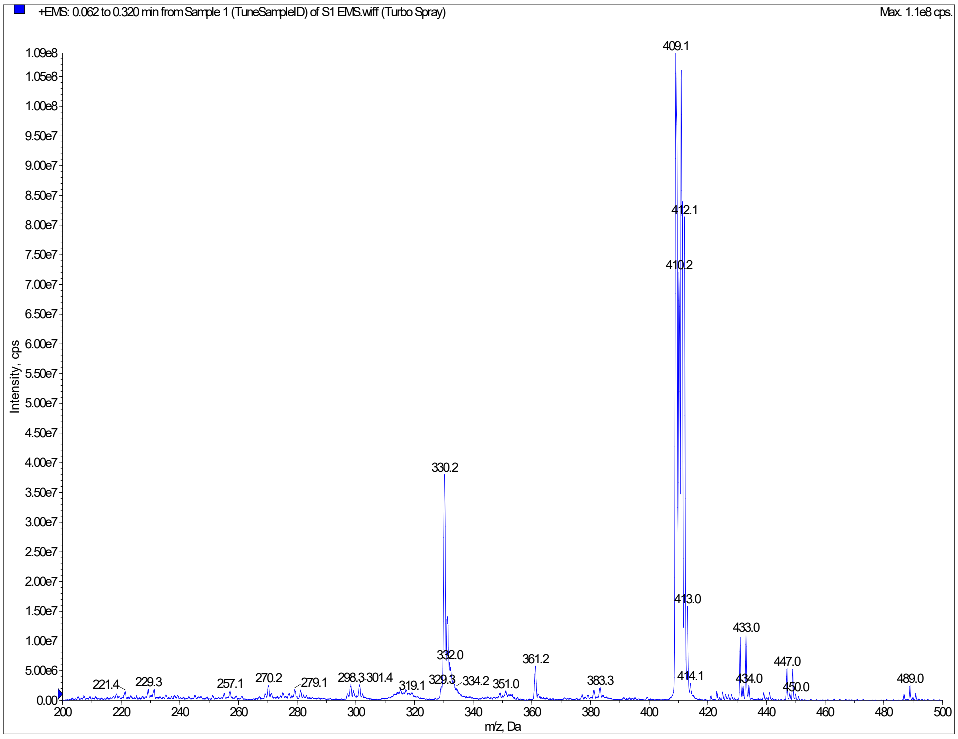

Figure S34. Fullscan MS spectrum of **2** $[M+H]^+$ (^{79}Br) 409.1 m/z, (^{81}Br) 411.0 m/z

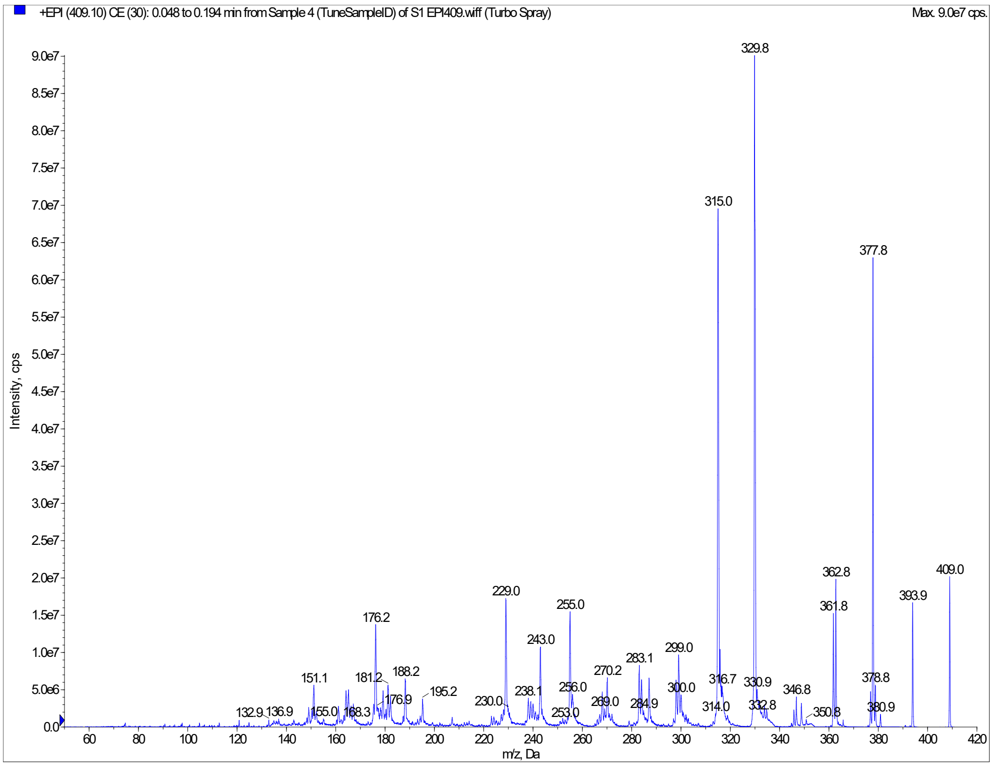

Figure S35. Fragmentation spectrum of **2** $[M+H]^+$ (^{79}Br) 409.1 m/z

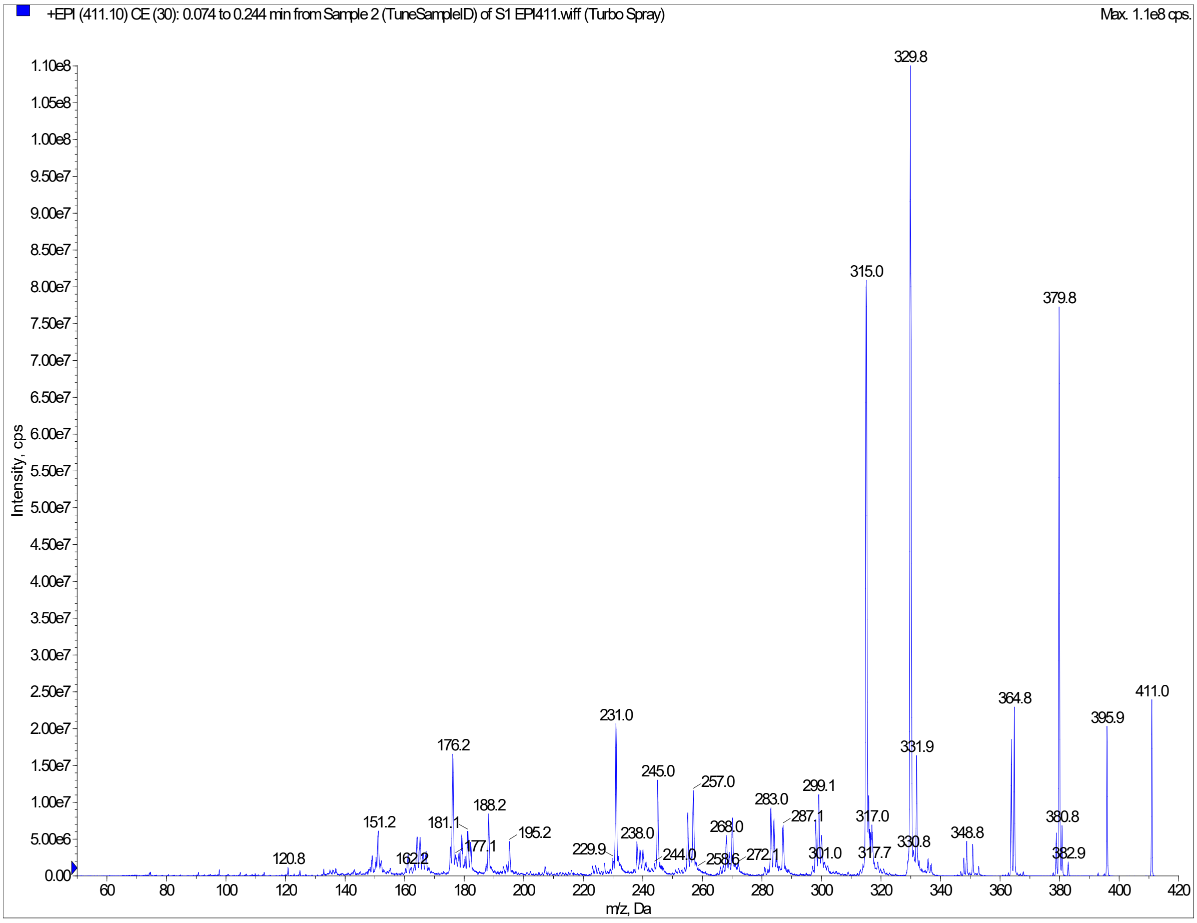

Figure S36. Fragmentation spectrum of **2** $[M+H]^+$ (^{81}Br) 411.0 m/z

2.3.ESI mass spectra and $[M+H]^+$ ion fragmentation of stilbene **3**

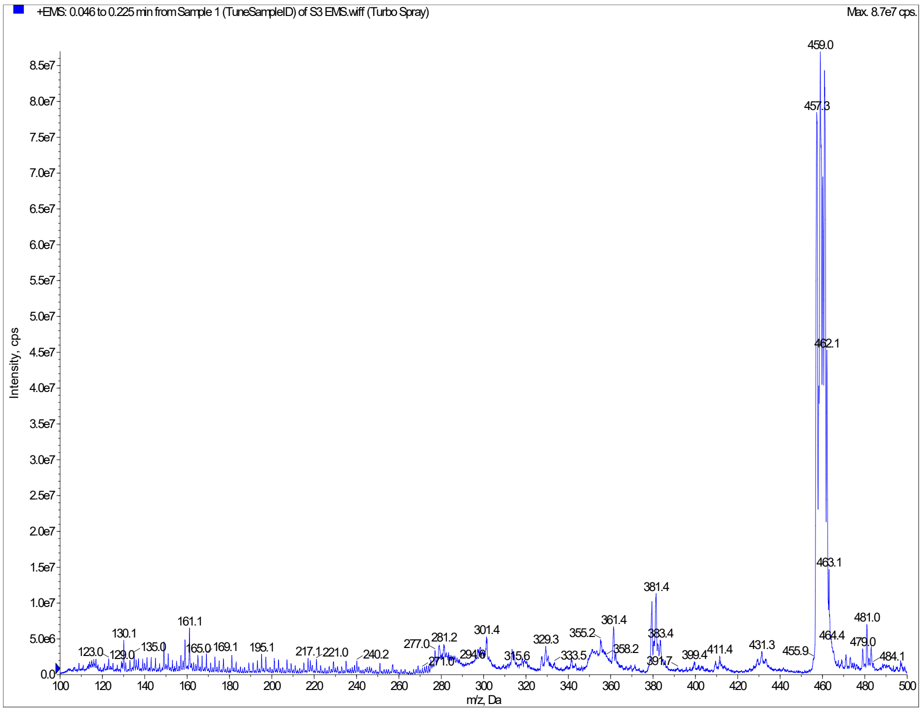

Figure S37. Fullscan MS spectrum of **3** $[M+H]^+$ (^{79}Br, ^{81}Br) 458.9 m/z, (2x ^{81}Br) 461.0 m/z, (2x ^{79}Br) 457.2 m/z

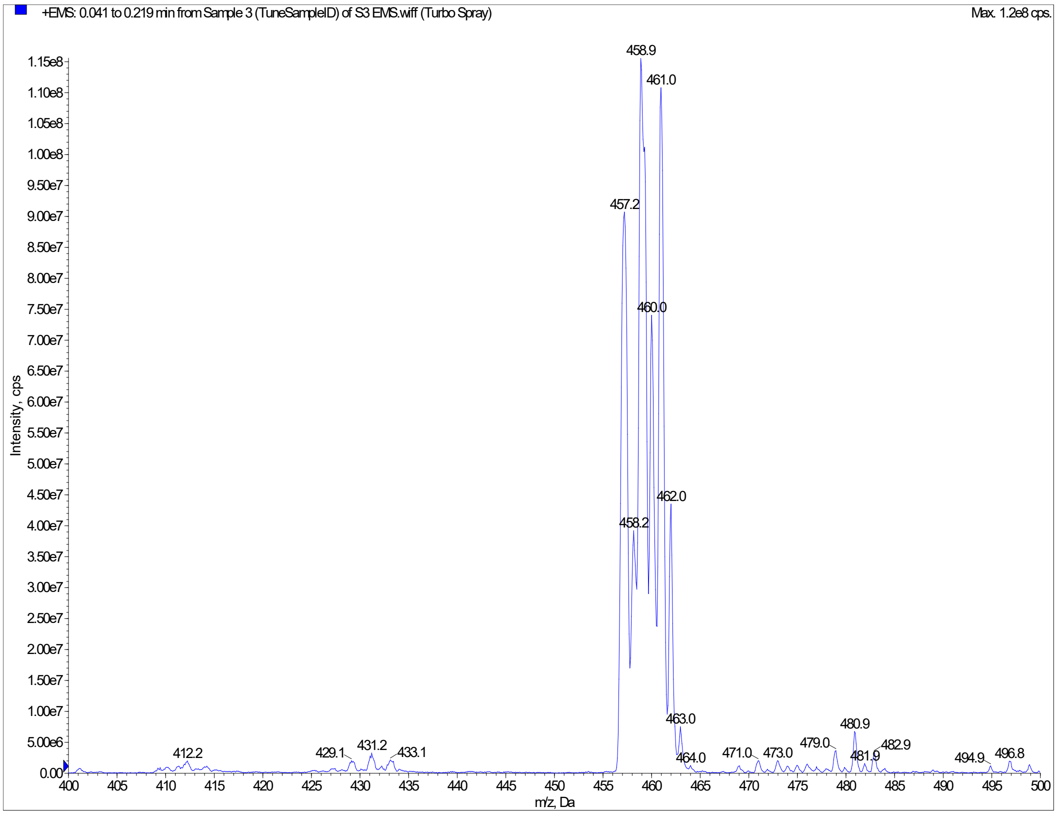

Figure S38. Fullscan MS spectrum of **3** $[M+H]^+$ (^{79}Br, ^{81}Br) 458.9 m/z, (2x ^{81}Br) 461.0 m/z, (2x ^{79}Br) 457.2 m/z

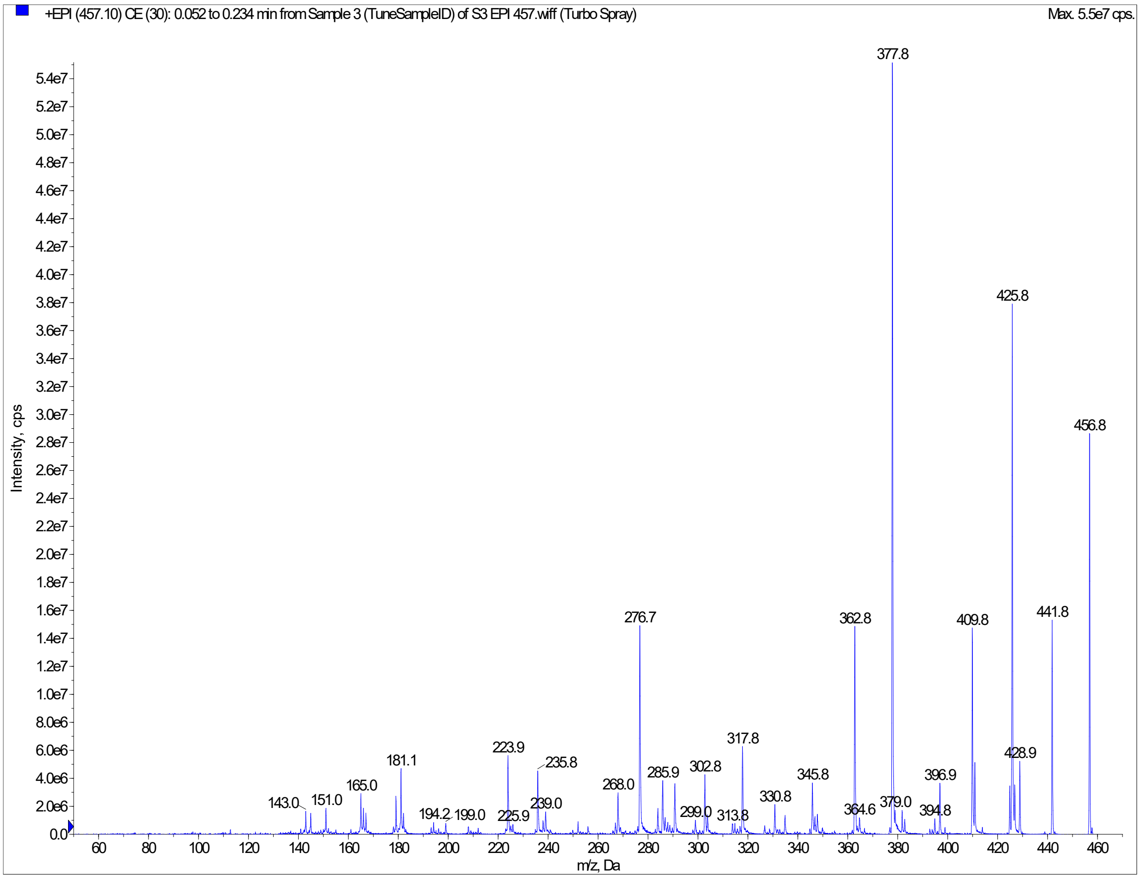

Figure S39. Fragmentation spectrum of **3** [M+H]$^+$ (2x ^{79}Br) 457.2 m/z

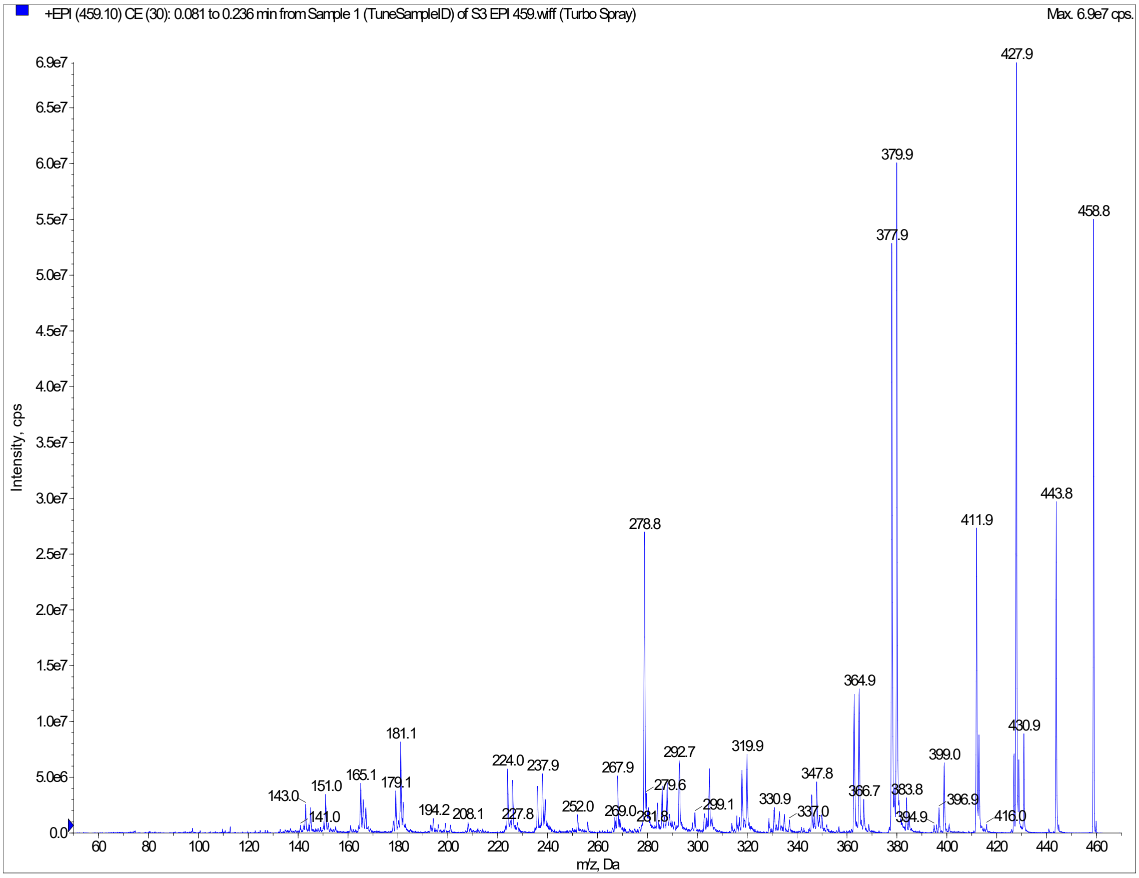

Figure S40. Fragmentation spectrum of **3** [M+H]$^+$ (^{79}Br, ^{81}Br) 458.9 m/z

2.4. ESI mass spectra and $[M+H]^+$ ion fragmentation of stilbene **4**

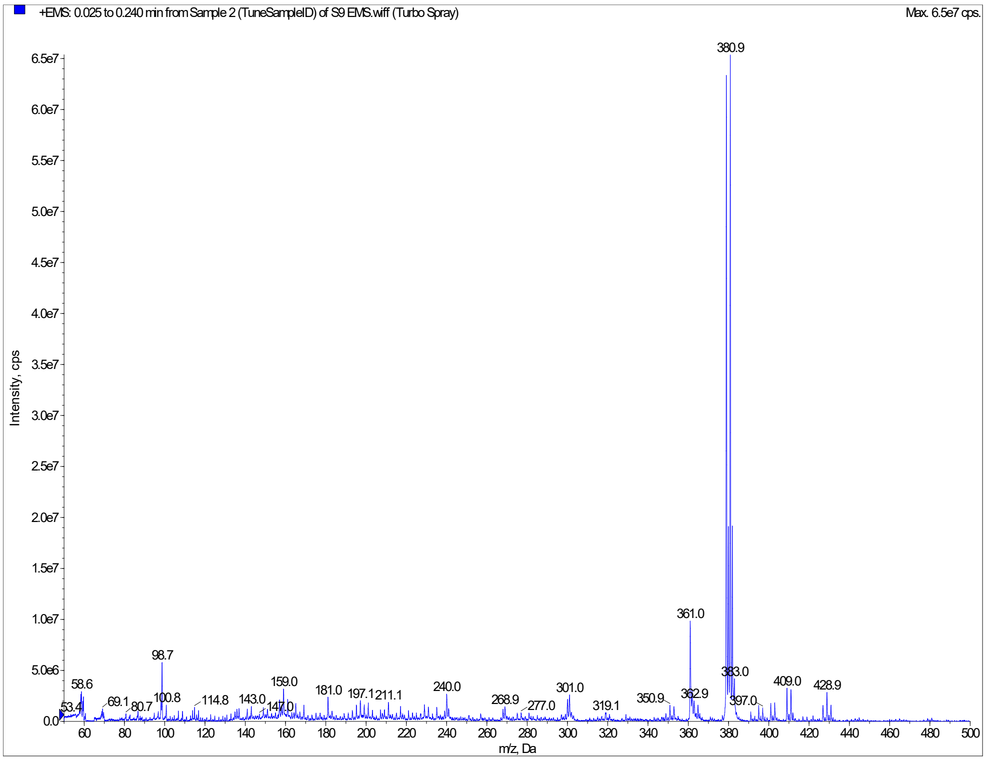

Figure S41. Fullscan MS spectrum of **4** $[M+H]^+$ (^{79}Br) 379.0 m/z, (^{81}Br) 380.9 m/z

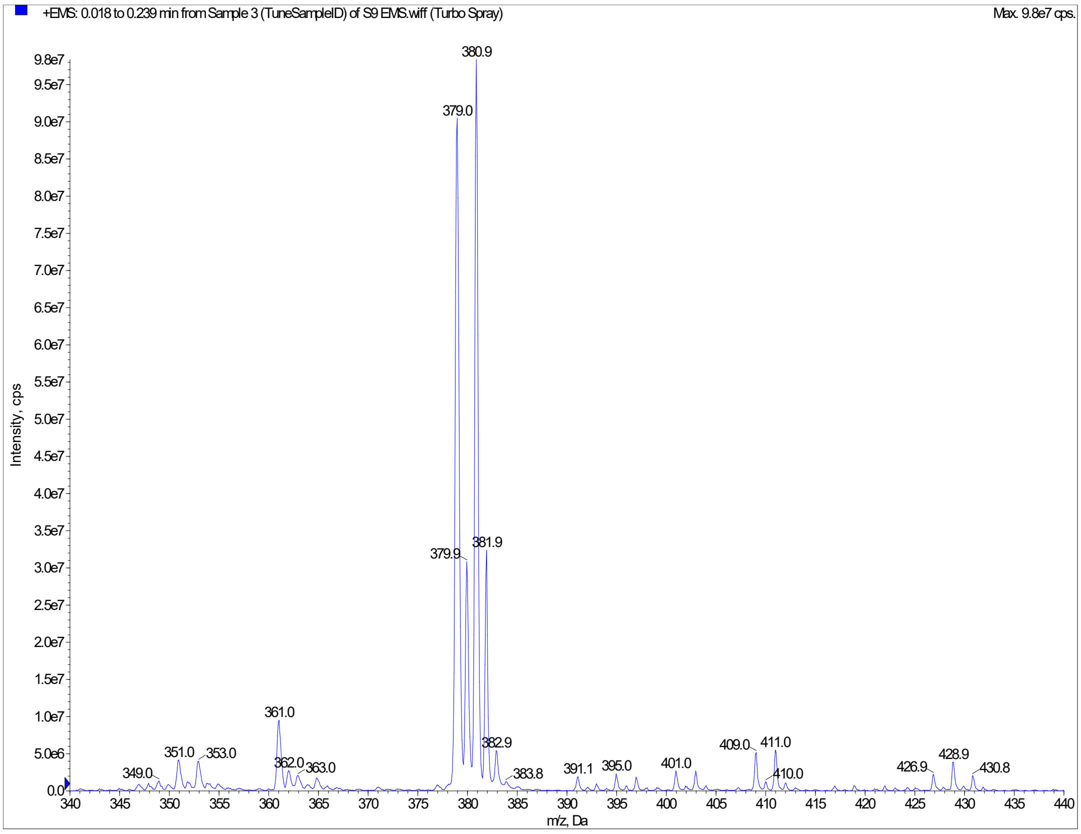

Figure S42. Fullscan MS spectrum of **4** $[M+H]^+$ (^{79}Br) 379.0 m/z, (^{81}Br) 380.9 m/z

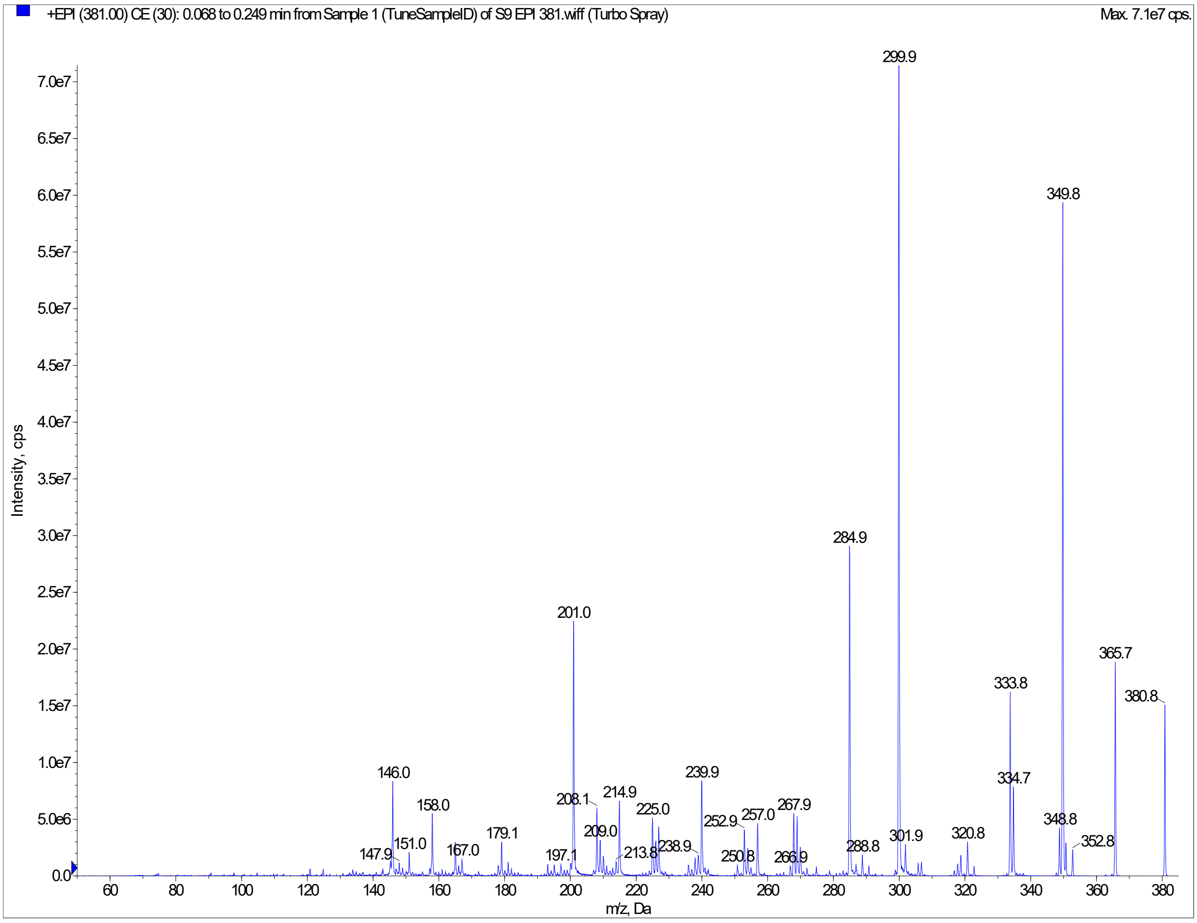

Figure S43. Fragmentation spectrum of **4** [M+H]+ ([81]Br) 380.9 m/z

2.5. ESI mass spectra and $[M+H]^+$ ion fragmentation of stilbene **5**

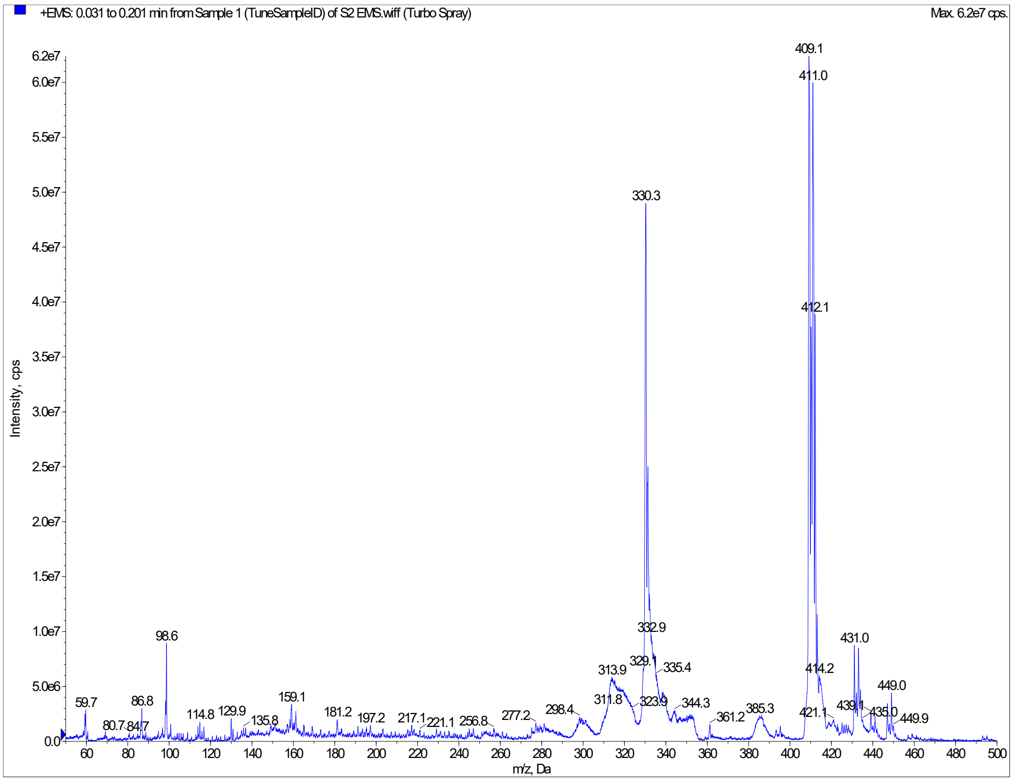

Figure S44. Fullscan MS spectrum of **5** $[M+H]^+$ (^{79}Br) 409.1 m/z, (^{81}Br) 411.0 m/z

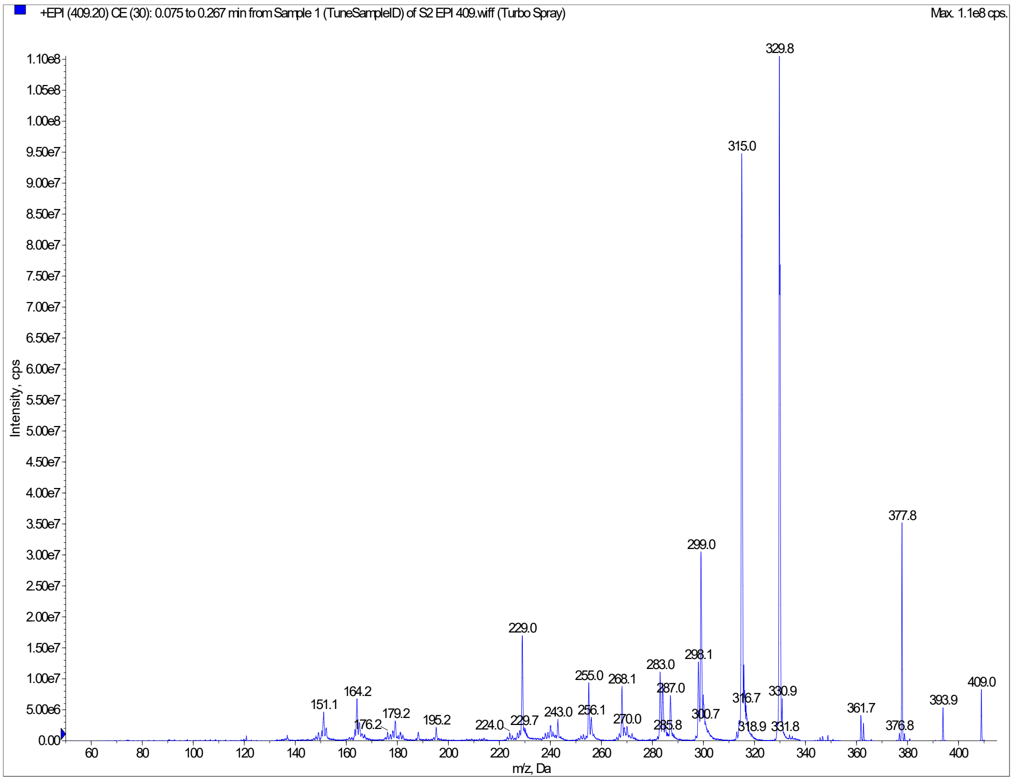

Figure S45. Fragmentation spectrum of **5** $[M+H]^+$ (^{79}Br) 409.1 m/z

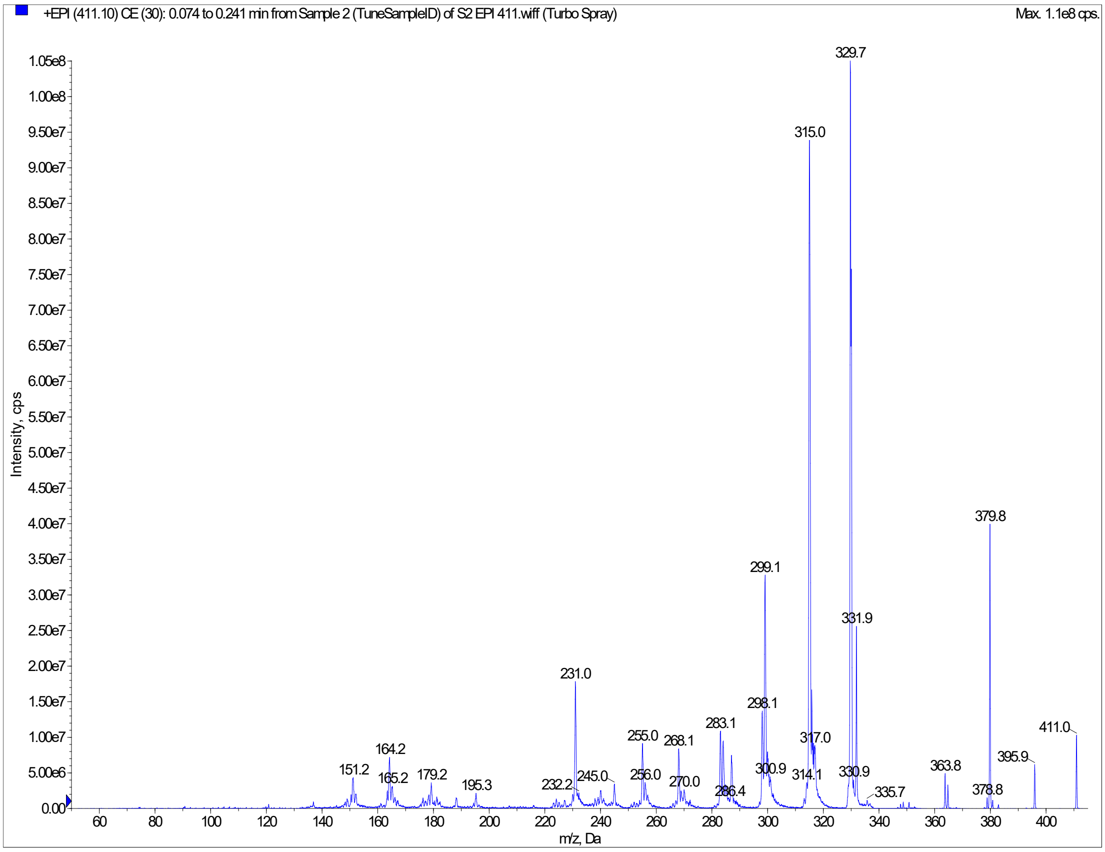

Figure S46. Fragmentation spectrum of **5** [M+H]$^+$ (^{81}Br) 411.0 m/z

2.6. ESI mass spectra and $[M+H]^+$ ion fragmentation of stilbene **6**

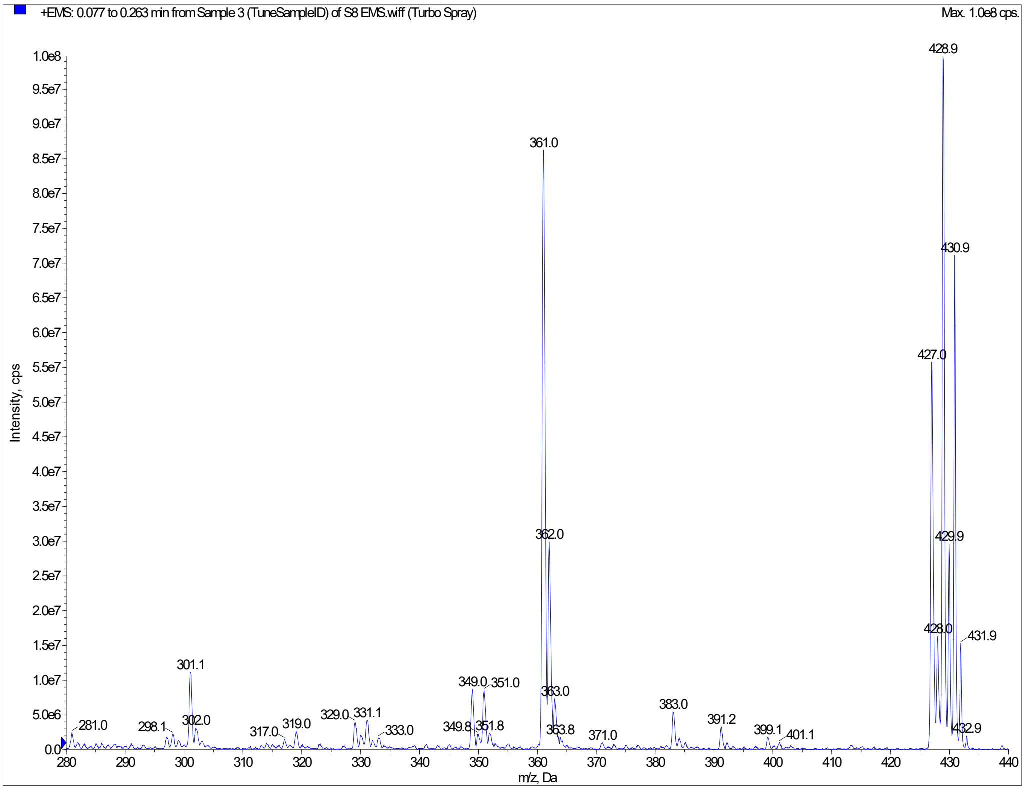

Figure S47. Fullscan MS spectrum of **6** $[M+H]^+$ (^{79}Br, ^{81}Br) 428.9 m/z, (2x ^{81}Br) 430.9 m/z, (2x ^{79}Br) 427.0 m/z

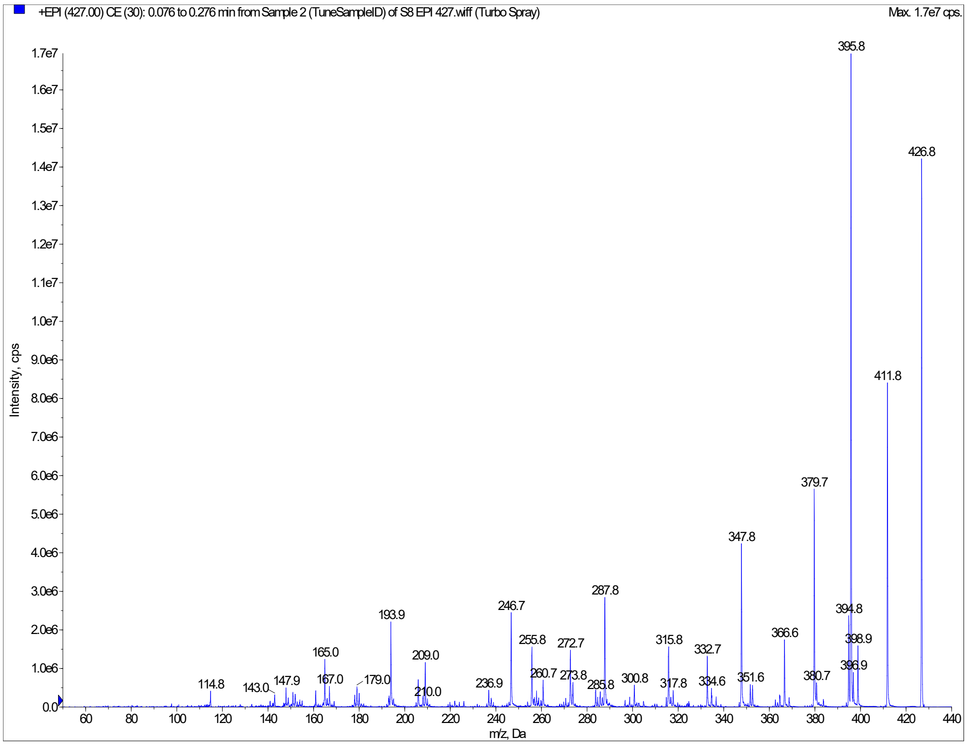

Figure S48. Fragmentation spectrum of **6** $[M+H]^+$ (2x ^{79}Br) 427.0 m/z

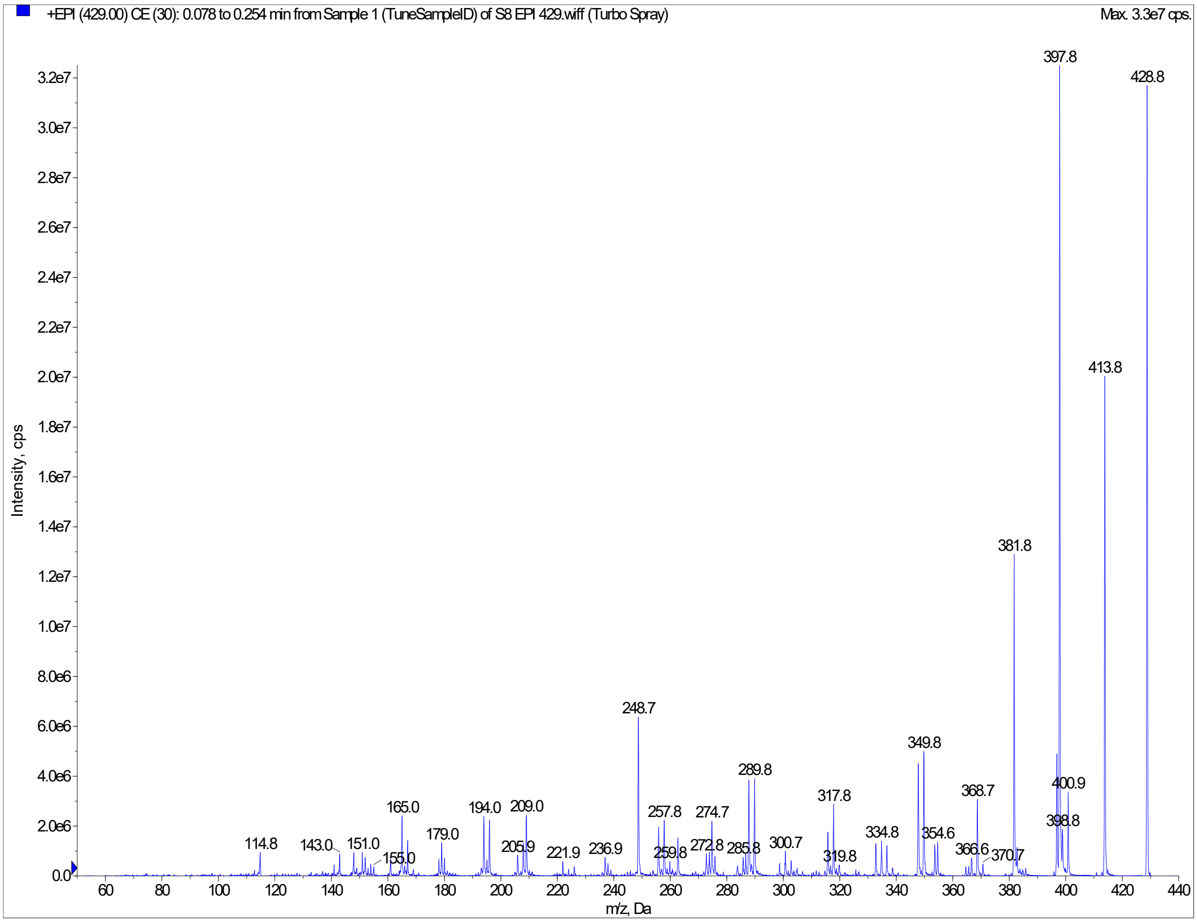

Figure S49. Fragmentation spectrum of **6** $[M+H]^+$ (^{79}Br, ^{81}Br) 428.9 m/z

3. UV-vis spectra

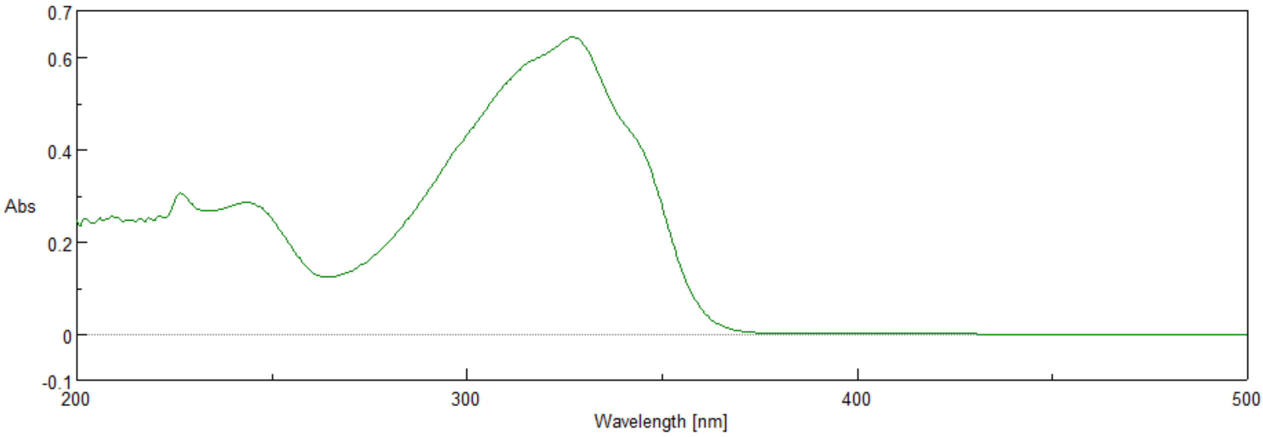

Figure S50. UV-Vis spectrum of stilbene **1** in DCM

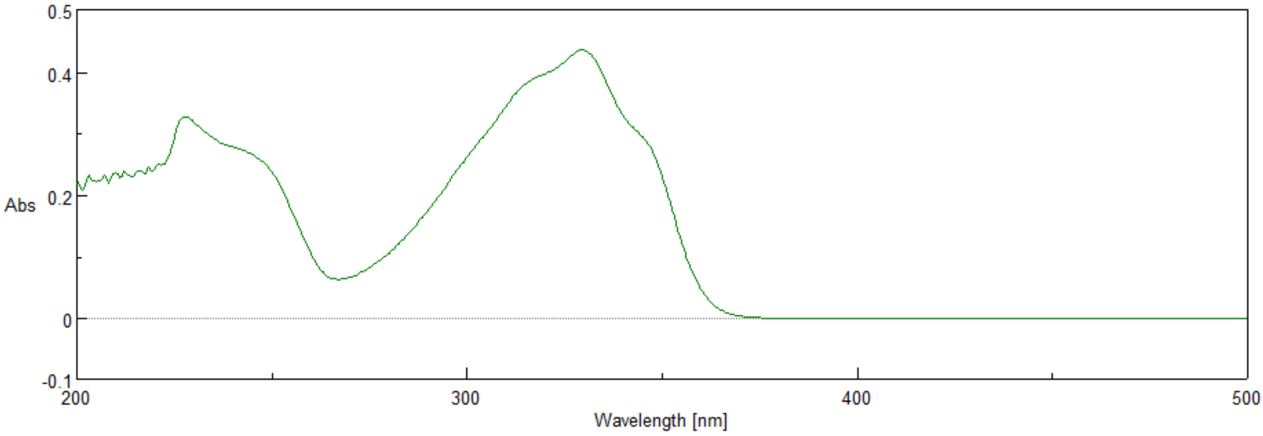

Figure S51. UV-Vis spectrum of stilbene **2** in DCM

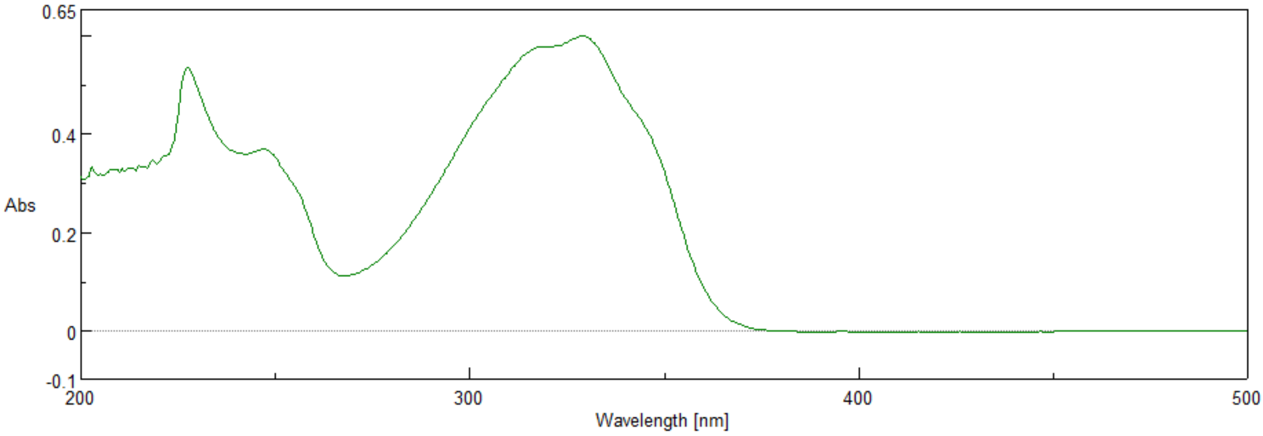

Figure S52. UV-Vis spectrum of stilbene **3** in DCM

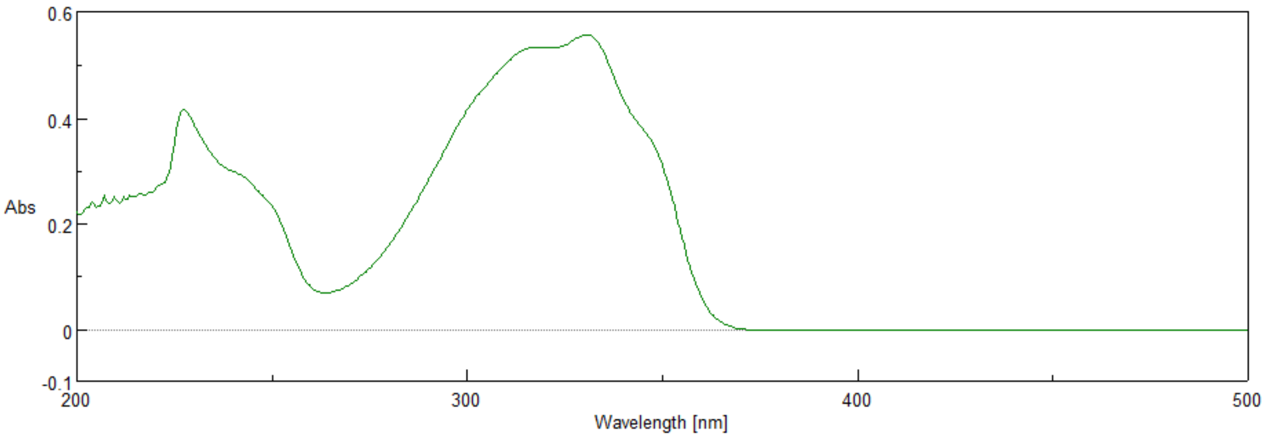

Figure S53. UV-Vis spectrum of stilbene **4** in DCM

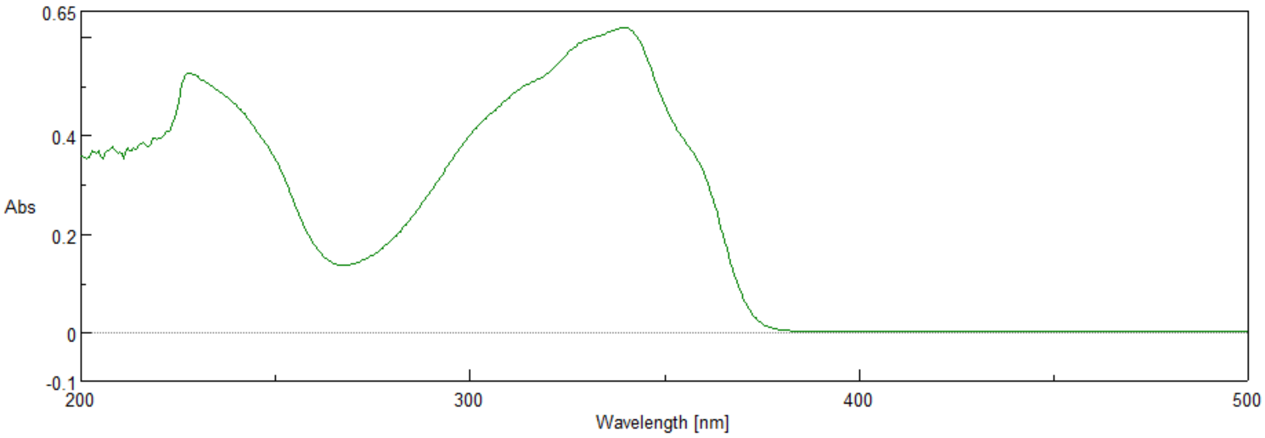

Figure S54. UV-Vis spectrum of stilbene **5** in DCM

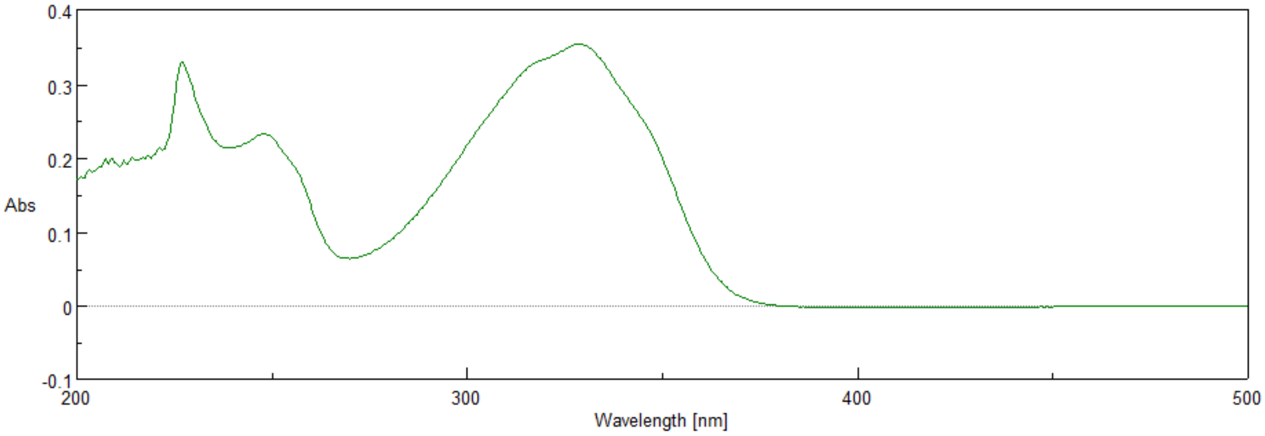

Figure S55. UV-Vis spectrum of stilbene **6** in DCM

4. Crystallographic data

Table S1. Atomic coordinates and Ueq [Å^2] for **3**.

Atom	x	y	z	U_{eq}
Br23	0.3201(2)	0.25986(7)	0.35376(6)	0.0244(3)
Br24	−0.2336(2)	0.67824(8)	0.33612(6)	0.0278(3)
O21	−0.0542(15)	0.4529(5)	0.2769(4)	0.0188(15)
O15	0.9652(17)	0.3487(6)	0.9353(4)	0.0247(16)
O19	0.3787(16)	0.6673(6)	0.9170(4)	0.0253(15)
O17	0.7434(15)	0.5224(6)	1.0047(4)	0.0219(15)
C2	0.044(2)	0.4688(9)	0.3572(6)	0.018(2)
C14	0.734(2)	0.4058(9)	0.8012(6)	0.021(2)
H14	0.823714	0.347605	0.776509	0.025
C3	0.208(2)	0.3898(8)	0.4052(6)	0.021(2)
C9	0.545(2)	0.4786(8)	0.7537(6)	0.018(2)
C4	0.289(2)	0.4027(8)	0.4875(7)	0.023(2)
H4	0.402350	0.348822	0.517084	0.028
C10	0.419(2)	0.5669(8)	0.7924(6)	0.020(2)
H10	0.290657	0.616036	0.761712	0.024
C6	0.046(3)	0.5774(9)	0.4800(6)	0.025(2)
H6	−0.008382	0.641621	0.504120	0.030
C1	−0.028(2)	0.5628(8)	0.3979(7)	0.022(2)
C5	0.202(2)	0.4974(8)	0.5270(6)	0.020(2)
C13	0.792(2)	0.4189(8)	0.8842(7)	0.021(2)
C11	0.481(2)	0.5820(8)	0.8747(6)	0.020(2)
C7	0.278(3)	0.5135(8)	0.6154(7)	0.024(2)
H7	0.168023	0.568292	0.638349	0.028
C12	0.667(2)	0.5075(8)	0.9225(7)	0.020(2)
C8	0.482(2)	0.4602(9)	0.6655(6)	0.019(2)
H8	0.596985	0.405984	0.643408	0.023
C22	0.175(2)	0.4822(9)	0.2232(6)	0.024(2)
H22A	0.089867	0.469388	0.167973	0.036
H22B	0.360960	0.439158	0.235781	0.036
H22C	0.227349	0.557393	0.230275	0.036
C20	0.180(2)	0.7435(9)	0.8746(7)	0.029(2)
H20A	0.293846	0.782351	0.837201	0.043
H20B	0.104449	0.793402	0.912481	0.043
H20C	0.004756	0.706883	0.845269	0.043
C16	1.098(2)	0.2570(10)	0.9017(6)	0.030(2)
H16A	1.234151	0.279806	0.862668	0.045
H16B	0.933404	0.212397	0.875331	0.045
H16C	1.216270	0.216476	0.944046	0.045
C18	0.489(2)	0.5057(10)	1.0522(7)	0.029(3)
H18A	0.322907	0.555523	1.035246	0.044
H18B	0.561020	0.517419	1.108511	0.044
H18C	0.413428	0.432859	1.044725	0.044

U_{eq} is defined as 1/3 of the trace of the orthogonalized U_{ij} tensor.

Table S2. Anisotropic displacement parameters (Å^2) for mw241122. The anisotropic displacement factor exponent takes the form: $-2\pi^2[\ h^2(a^*)^2U_{11} + k^2(b^*)^2U_{22} + \ldots + 2hka^*b^*U_{12}\ ]$

**Atom**	U_{11}	U_{22}	U_{33}	U_{23}	U_{13}	U_{12}
Br23	0.0270(5)	0.0183(5)	0.0282(5)	−0.0025(5)	0.0045(4)	0.0026(4)
Br24	0.0314(6)	0.0217(5)	0.0296(6)	0.0004(5)	0.0005(4)	0.0078(5)
O21	0.017(3)	0.024(4)	0.014(4)	−0.002(3)	−0.001(3)	−0.003(3)
O15	0.028(4)	0.023(4)	0.022(4)	0.001(3)	0.000(3)	0.004(3)
O19	0.026(4)	0.022(4)	0.029(4)	−0.004(3)	0.007(3)	0.001(3)
O17	0.018(4)	0.033(4)	0.015(4)	−0.001(3)	0.002(3)	−0.001(3)
C2	0.010(4)	0.020(5)	0.025(6)	−0.001(4)	0.005(4)	0.001(3)
C14	0.022(5)	0.023(6)	0.019(6)	−0.003(4)	0.007(4)	−0.004(4)
C3	0.026(5)	0.015(5)	0.023(6)	−0.004(4)	0.010(4)	−0.007(4)
C9	0.022(5)	0.017(5)	0.014(5)	0.004(4)	0.000(4)	−0.009(4)
C4	0.018(5)	0.020(5)	0.031(6)	0.009(4)	0.003(4)	0.004(4)
C10	0.021(5)	0.016(5)	0.023(5)	0.007(4)	0.000(4)	0.004(4)
C6	0.031(6)	0.022(5)	0.022(5)	0.002(4)	0.008(4)	0.003(4)
C1	0.023(5)	0.018(5)	0.026(6)	−0.004(4)	0.008(4)	−0.003(4)
C5	0.023(5)	0.016(5)	0.022(5)	−0.006(4)	0.003(4)	−0.011(4)
C13	0.011(5)	0.015(5)	0.038(6)	−0.002(4)	0.007(4)	−0.008(4)
C11	0.018(5)	0.015(5)	0.025(6)	−0.003(4)	0.002(4)	−0.005(4)
C7	0.027(6)	0.019(5)	0.027(6)	−0.001(4)	0.014(5)	−0.003(4)
C12	0.013(5)	0.020(5)	0.025(6)	−0.001(4)	0.002(4)	−0.004(4)
C8	0.019(5)	0.016(5)	0.022(6)	−0.002(4)	0.002(4)	−0.001(4)
C22	0.022(5)	0.031(6)	0.020(5)	0.007(5)	0.006(4)	0.004(4)

C20	0.029(5)	0.023(6)	0.034(6)	−0.003(5)	0.006(5)	0.007(5)
C16	0.032(5)	0.024(5)	0.032(6)	0.007(6)	0.000(4)	0.003(5)
C18	0.019(5)	0.039(7)	0.031(6)	−0.002(5)	0.008(5)	−0.006(5)

Table S3. Bond lengths and angles for **3.**

**Atom–Atom**	**Length [Å]**
Br23–C3	1.904(10)
Br24–C1	1.912(11)
O21–C2	1.363(12)
O21–C22	1.443(12)
O15–C13	1.371(13)
O15–C16	1.409(14)
O19–C11	1.366(12)
O19–C20	1.404(12)
O17–C12	1.376(13)
O17–C18	1.427(12)
C2–C3	1.398(15)
C2–C1	1.393(14)
C14–H14	0.9300
C14–C9	1.395(15)
C14–C13	1.379(15)
C3–C4	1.377(15)
C9–C10	1.403(14)
C9–C8	1.472(14)
C4–H4	0.9300
C4–C5	1.411(14)
C10–H10	0.9300
C10–C11	1.372(15)
C6–H6	0.9300
C6–C1	1.373(16)
C6–C5	1.382(15)
C5–C7	1.478(15)
C13–C12	1.401(14)
C11–C12	1.403(14)
C7–H7	0.9300
C7–C8	1.314(15)
C8–H8	0.9300
C22–H22A	0.9600
C22–H22B	0.9600
C22–H22C	0.9600
C20–H20A	0.9600
C20–H20B	0.9600
C20–H20C	0.9600
C16–H16A	0.9600
C16–H16B	0.9600
C16–H16C	0.9600
C18–H18A	0.9600
C18–H18B	0.9600
C18–H18C	0.9600
**Atom–Atom–Atom**	**Angle [°]**
C2–O21–C22	114.1(8)
C13–O15–C16	118.6(8)

C11–O19–C20	118.2(8)
C12–O17–C18	114.5(8)
O21–C2–C3	122.3(9)
O21–C2–C1	122.2(10)
C1–C2–C3	115.5(9)
C9–C14–H14	119.5
C13–C14–H14	119.5
C13–C14–C9	120.9(10)
C2–C3–Br23	118.0(8)
C4–C3–Br23	119.4(8)
C4–C3–C2	122.6(9)
C14–C9–C10	118.2(9)
C14–C9–C8	119.3(9)
C10–C9–C8	122.5(9)
C3–C4–H4	119.9
C3–C4–C5	120.2(9)
C5–C4–H4	119.9
C9–C10–H10	119.4
C11–C10–C9	121.2(9)
C11–C10–H10	119.4
C1–C6–H6	119.8
C1–C6–C5	120.5(10)
C5–C6–H6	119.8
C2–C1–Br24	118.4(8)
C6–C1–Br24	118.2(8)

C6–C1–C2	123.3(10)
C4–C5–C7	122.0(10)
C6–C5–C4	117.9(9)
C6–C5–C7	120.0(9)
O15–C13–C14	124.4(9)
O15–C13–C12	115.0(10)
C14–C13–C12	120.6(10)
O19–C11–C10	125.2(9)
O19–C11–C12	114.2(9)
C10–C11–C12	120.5(9)
C5–C7–H7	116.0
C8–C7–C5	128.1(10)
C8–C7–H7	116.0
O17–C12–C13	119.5(9)
O17–C12–C11	121.9(9)
C13–C12–C11	118.5(10)
C9–C8–H8	117.1
C7–C8–C9	125.7(10)
C7–C8–H8	117.1
O21–C22–H22A	109.5
O21–C22–H22B	109.5
O21–C22–H22C	109.5
H22A–C22–H22B	109.5
H22A–C22–H22C	109.5
H22B–C22–H22C	109.5

O19–C20–H20A	109.5
O19–C20–H20B	109.5
O19–C20–H20C	109.5
H20A–C20–H20B	109.5
H20A–C20–H20C	109.5
H20B–C20–H20C	109.5
O15–C16–H16A	109.5
O15–C16–H16B	109.5
O15–C16–H16C	109.5
H16A–C16–H16B	109.5

H16A–C16–H16C	109.5
H16B–C16–H16C	109.5
O17–C18–H18A	109.5
O17–C18–H18B	109.5
O17–C18–H18C	109.5
H18A–C18–H18B	109.5
H18A–C18–H18C	109.5
H18B–C18–H18C	109.5

Table S4. Torsion angles for **3**.

Atom–Atom–Atom–Atom	Torsion Angle [°]
Br23–C3–C4–C5	−178.6(7)
O21–C2–C3–Br23	4.3(13)
O21–C2–C3–C4	−175.7(9)
O21–C2–C1–Br24	−6.3(13)
O21–C2–C1–C6	174.8(9)
O15–C13–C12–O17	4.9(13)
O15–C13–C12–C11	−178.7(9)
O19–C11–C12–O17	−1.7(14)
O19–C11–C12–C13	−178.0(8)
C2–C3–C4–C5	1.4(16)
C14–C9–C10–C11	0.3(15)
C14–C9–C8–C7	170.9(11)
C14–C13–C12–O17	−176.0(9)
C14–C13–C12–C11	0.4(14)
C3–C2–C1–Br24	177.0(7)
C3–C2–C1–C6	−2.0(15)
C3–C4–C5–C6	−2.9(15)
C3–C4–C5–C7	178.6(9)
C9–C14–C13–O15	177.3(9)
C9–C14–C13–C12	−1.7(15)
C9–C10–C11–O19	177.6(9)
C9–C10–C11–C12	−1.7(15)
C4–C5–C7–C8	15.7(16)
C10–C9–C8–C7	−8.8(17)
C10–C11–C12–O17	177.6(9)
C10–C11–C12–C13	1.3(15)
C6–C5–C7–C8	−162.7(11)
C1–C2–C3–Br23	−179.0(7)
C1–C2–C3–C4	1.0(15)
C1–C6–C5–C4	2.0(15)
C1–C6–C5–C7	−179.5(10)
C5–C6–C1–Br24	−178.5(8)
C5–C6–C1–C2	0.4(16)
C5–C7–C8–C9	−178.8(10)
C13–C14–C9–C10	1.4(15)
C13–C14–C9–C8	−178.4(9)
C8–C9–C10–C11	−180.0(10)
C22–O21–C2–C3	−88.0(11)
C22–O21–C2–C1	95.5(11)
C20–O19–C11–C10	3.5(14)
C20–O19–C11–C12	−177.2(9)
C16–O15–C13–C14	0.6(14)
C16–O15–C13–C12	179.7(9)
C18–O17–C12–C13	−111.2(10)
C18–O17–C12–C11	72.6(12)

5. Computational data

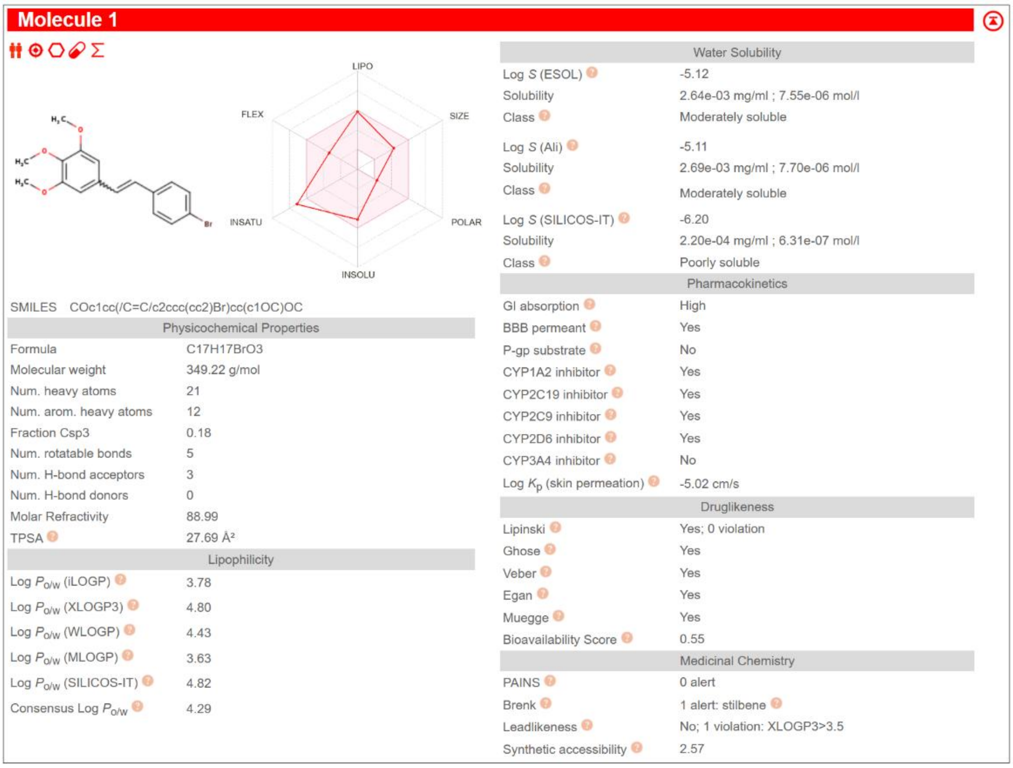

Molecule 1

SMILES COc1cc(/C=C/c2ccc(cc2)Br)cc(c1OC)OC

Physicochemical Properties	
Formula	C17H17BrO3
Molecular weight	349.22 g/mol
Num. heavy atoms	21
Num. arom. heavy atoms	12
Fraction Csp3	0.18
Num. rotatable bonds	5
Num. H-bond acceptors	3
Num. H-bond donors	0
Molar Refractivity	88.99
TPSA	27.69 Å²

Lipophilicity	
Log $P_{o/w}$ (iLOGP)	3.78
Log $P_{o/w}$ (XLOGP3)	4.80
Log $P_{o/w}$ (WLOGP)	4.43
Log $P_{o/w}$ (MLOGP)	3.63
Log $P_{o/w}$ (SILICOS-IT)	4.82
Consensus Log $P_{o/w}$	4.29

Water Solubility	
Log *S* (ESOL)	-5.12
Solubility	2.64e-03 mg/ml ; 7.55e-06 mol/l
Class	Moderately soluble
Log *S* (Ali)	-5.11
Solubility	2.69e-03 mg/ml ; 7.70e-06 mol/l
Class	Moderately soluble
Log *S* (SILICOS-IT)	-6.20
Solubility	2.20e-04 mg/ml ; 6.31e-07 mol/l
Class	Poorly soluble

Pharmacokinetics	
GI absorption	High
BBB permeant	Yes
P-gp substrate	No
CYP1A2 inhibitor	Yes
CYP2C19 inhibitor	Yes
CYP2C9 inhibitor	Yes
CYP2D6 inhibitor	Yes
CYP3A4 inhibitor	No
Log K_p (skin permeation)	-5.02 cm/s

Druglikeness	
Lipinski	Yes; 0 violation
Ghose	Yes
Veber	Yes
Egan	Yes
Muegge	Yes
Bioavailability Score	0.55

Medicinal Chemistry	
PAINS	0 alert
Brenk	1 alert: stilbene
Leadlikeness	No; 1 violation: XLOGP3>3.5
Synthetic accessibility	2.57

Figure S56. SwissDrugDesign module prediction for compound **1**

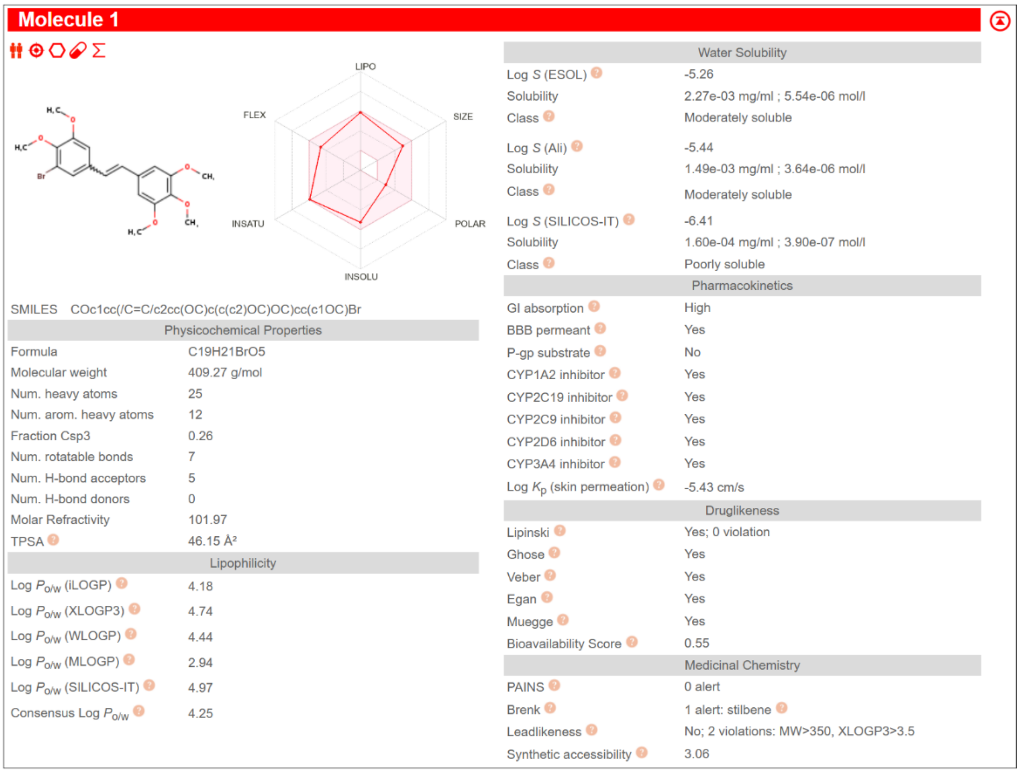

Molecule 1

SMILES COc1cc(/C=C/c2cc(OC)c(c(c2)OC)OC)cc(c1OC)Br

Physicochemical Properties	
Formula	C19H21BrO5
Molecular weight	409.27 g/mol
Num. heavy atoms	25
Num. arom. heavy atoms	12
Fraction Csp3	0.26
Num. rotatable bonds	7
Num. H-bond acceptors	5
Num. H-bond donors	0
Molar Refractivity	101.97
TPSA	46.15 Å²

Lipophilicity	
Log $P_{o/w}$ (iLOGP)	4.18
Log $P_{o/w}$ (XLOGP3)	4.74
Log $P_{o/w}$ (WLOGP)	4.44
Log $P_{o/w}$ (MLOGP)	2.94
Log $P_{o/w}$ (SILICOS-IT)	4.97
Consensus Log $P_{o/w}$	4.25

Water Solubility	
Log *S* (ESOL)	-5.26
Solubility	2.27e-03 mg/ml ; 5.54e-06 mol/l
Class	Moderately soluble
Log *S* (Ali)	-5.44
Solubility	1.49e-03 mg/ml ; 3.64e-06 mol/l
Class	Moderately soluble
Log *S* (SILICOS-IT)	-6.41
Solubility	1.60e-04 mg/ml ; 3.90e-07 mol/l
Class	Poorly soluble

Pharmacokinetics	
GI absorption	High
BBB permeant	Yes
P-gp substrate	No
CYP1A2 inhibitor	Yes
CYP2C19 inhibitor	Yes
CYP2C9 inhibitor	Yes
CYP2D6 inhibitor	Yes
CYP3A4 inhibitor	Yes
Log K_p (skin permeation)	-5.43 cm/s

Druglikeness	
Lipinski	Yes; 0 violation
Ghose	Yes
Veber	Yes
Egan	Yes
Muegge	Yes
Bioavailability Score	0.55

Medicinal Chemistry	
PAINS	0 alert
Brenk	1 alert: stilbene
Leadlikeness	No; 2 violations: MW>350, XLOGP3>3.5
Synthetic accessibility	3.06

Figure S57. SwissDrugDesign module prediction for compound **2**

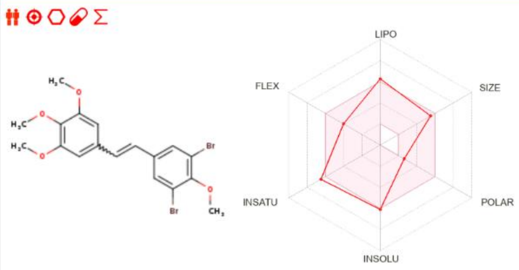

Molecule 1

SMILES COc1c(Br)cc(cc1Br)/C=C/c1cc(OC)c(c(c1)OC)OC

Physicochemical Properties	
Formula	C18H18Br2O4
Molecular weight	458.14 g/mol
Num. heavy atoms	24
Num. arom. heavy atoms	12
Fraction Csp3	0.22
Num. rotatable bonds	6
Num. H-bond acceptors	4
Num. H-bond donors	0
Molar Refractivity	103.18
TPSA	36.92 Å²
**Lipophilicity**	
Log $P_{o/w}$ (iLOGP)	4.27
Log $P_{o/w}$ (XLOGP3)	5.46
Log $P_{o/w}$ (WLOGP)	5.20
Log $P_{o/w}$ (MLOGP)	3.88
Log $P_{o/w}$ (SILICOS-IT)	5.58
Consensus Log $P_{o/w}$	4.88

Water Solubility	
Log *S* (ESOL)	-6.09
Solubility	3.69e-04 mg/ml ; 8.05e-07 mol/l
Class	Poorly soluble
Log *S* (Ali)	-5.99
Solubility	4.66e-04 mg/ml ; 1.02e-06 mol/l
Class	Moderately soluble
Log *S* (SILICOS-IT)	-7.09
Solubility	3.76e-05 mg/ml ; 8.20e-08 mol/l
Class	Poorly soluble
**Pharmacokinetics**	
GI absorption	High
BBB permeant	Yes
P-gp substrate	No
CYP1A2 inhibitor	Yes
CYP2C19 inhibitor	Yes
CYP2C9 inhibitor	Yes
CYP2D6 inhibitor	Yes
CYP3A4 inhibitor	No
Log K_p (skin permeation)	-5.22 cm/s
**Druglikeness**	
Lipinski	Yes; 0 violation
Ghose	Yes
Veber	Yes
Egan	Yes
Muegge	No; 1 violation: XLOGP3>5
Bioavailability Score	0.55
**Medicinal Chemistry**	
PAINS	0 alert
Brenk	1 alert: stilbene
Leadlikeness	No; 2 violations: MW>350, XLOGP3>3.5
Synthetic accessibility	2.90

Figure S58. SwissDrugDesign module prediction for compound **3**

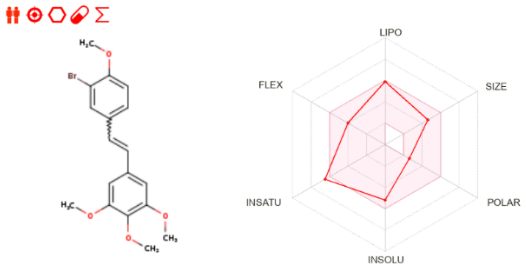

Molecule 1

SMILES COc1cc(/C=C/c2ccc(c(c2)Br)OC)cc(c1OC)OC

Physicochemical Properties	
Formula	C18H19BrO4
Molecular weight	379.25 g/mol
Num. heavy atoms	23
Num. arom. heavy atoms	12
Fraction Csp3	0.22
Num. rotatable bonds	6
Num. H-bond acceptors	4
Num. H-bond donors	0
Molar Refractivity	95.48
TPSA	36.92 Å²
**Lipophilicity**	
Log $P_{o/w}$ (iLOGP)	4.02
Log $P_{o/w}$ (XLOGP3)	4.77
Log $P_{o/w}$ (WLOGP)	4.44
Log $P_{o/w}$ (MLOGP)	3.28
Log $P_{o/w}$ (SILICOS-IT)	4.89
Consensus Log $P_{o/w}$	4.28

Water Solubility	
Log *S* (ESOL)	-5.19
Solubility	2.47e-03 mg/ml ; 6.51e-06 mol/l
Class	Moderately soluble
Log *S* (Ali)	-5.28
Solubility	2.01e-03 mg/ml ; 5.29e-06 mol/l
Class	Moderately soluble
Log *S* (SILICOS-IT)	-6.31
Solubility	1.87e-04 mg/ml ; 4.94e-07 mol/l
Class	Poorly soluble
**Pharmacokinetics**	
GI absorption	High
BBB permeant	Yes
P-gp substrate	No
CYP1A2 inhibitor	Yes
CYP2C19 inhibitor	Yes
CYP2C9 inhibitor	Yes
CYP2D6 inhibitor	Yes
CYP3A4 inhibitor	Yes
Log K_p (skin permeation)	-5.23 cm/s
**Druglikeness**	
Lipinski	Yes; 0 violation
Ghose	Yes
Veber	Yes
Egan	Yes
Muegge	Yes
Bioavailability Score	0.55
**Medicinal Chemistry**	
PAINS	0 alert
Brenk	1 alert: stilbene
Leadlikeness	No; 2 violations: MW>350, XLOGP3>3.5
Synthetic accessibility	2.81

Figure S59. SwissDrugDesign module prediction for compound **4**

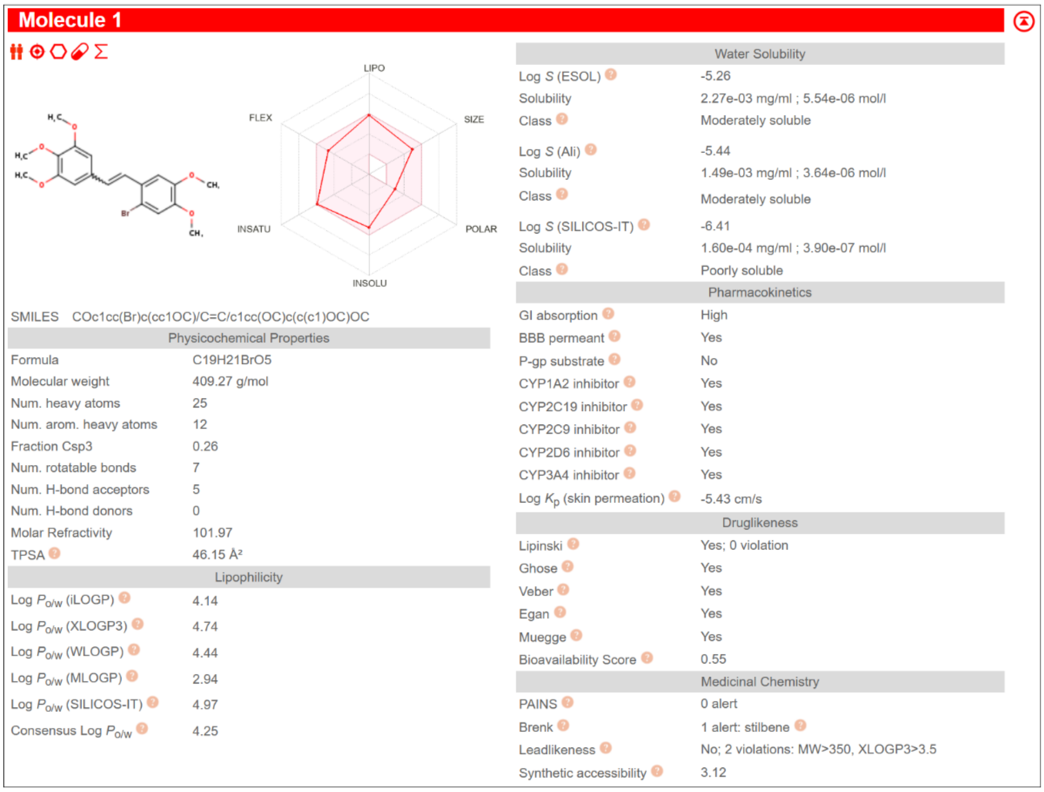

Molecule 1

SMILES COc1cc(Br)c(cc1OC)/C=C/c1cc(OC)c(c(c1)OC)OC

Physicochemical Properties	
Formula	C19H21BrO5
Molecular weight	409.27 g/mol
Num. heavy atoms	25
Num. arom. heavy atoms	12
Fraction Csp3	0.26
Num. rotatable bonds	7
Num. H-bond acceptors	5
Num. H-bond donors	0
Molar Refractivity	101.97
TPSA	46.15 Å²

Lipophilicity	
Log $P_{o/w}$ (iLOGP)	4.14
Log $P_{o/w}$ (XLOGP3)	4.74
Log $P_{o/w}$ (WLOGP)	4.44
Log $P_{o/w}$ (MLOGP)	2.94
Log $P_{o/w}$ (SILICOS-IT)	4.97
Consensus Log $P_{o/w}$	4.25

Water Solubility	
Log *S* (ESOL)	-5.26
Solubility	2.27e-03 mg/ml ; 5.54e-06 mol/l
Class	Moderately soluble
Log *S* (Ali)	-5.44
Solubility	1.49e-03 mg/ml ; 3.64e-06 mol/l
Class	Moderately soluble
Log *S* (SILICOS-IT)	-6.41
Solubility	1.60e-04 mg/ml ; 3.90e-07 mol/l
Class	Poorly soluble

Pharmacokinetics	
GI absorption	High
BBB permeant	Yes
P-gp substrate	No
CYP1A2 inhibitor	Yes
CYP2C19 inhibitor	Yes
CYP2C9 inhibitor	Yes
CYP2D6 inhibitor	Yes
CYP3A4 inhibitor	Yes
Log K_p (skin permeation)	-5.43 cm/s

Druglikeness	
Lipinski	Yes; 0 violation
Ghose	Yes
Veber	Yes
Egan	Yes
Muegge	Yes
Bioavailability Score	0.55

Medicinal Chemistry	
PAINS	0 alert
Brenk	1 alert: stilbene
Leadlikeness	No; 2 violations: MW>350, XLOGP3>3.5
Synthetic accessibility	3.12

Figure S60. SwissDrugDesign module prediction for compound **5**

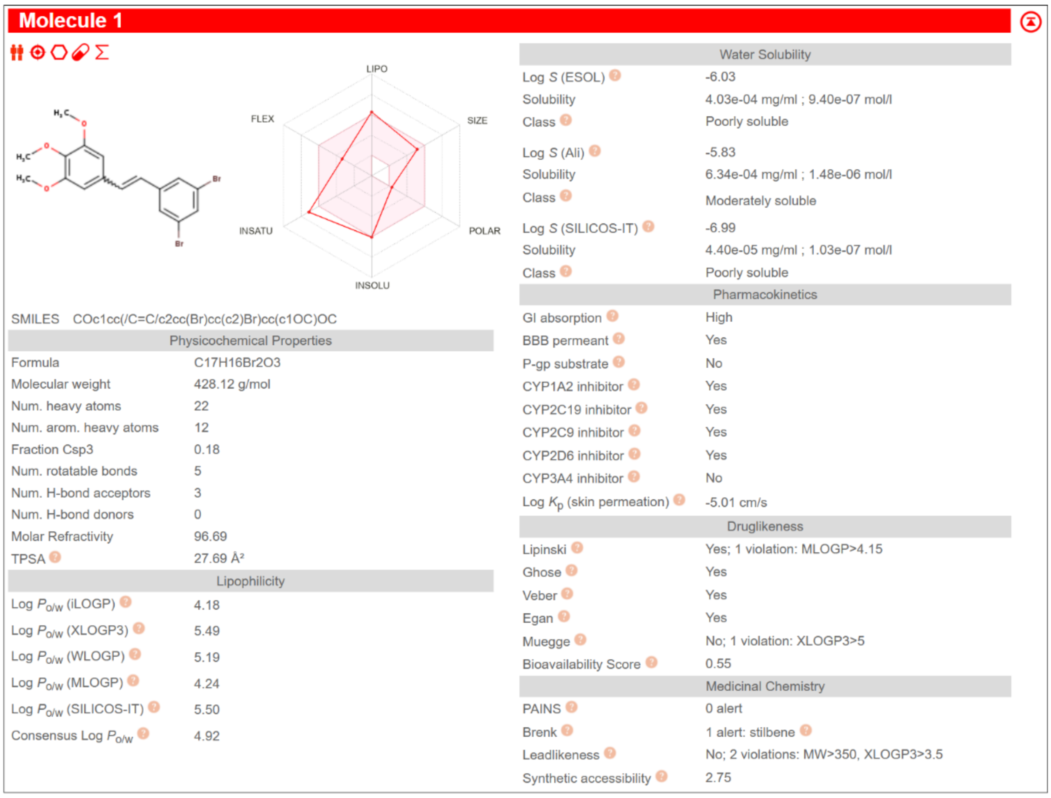

Molecule 1

SMILES COc1cc(/C=C/c2cc(Br)cc(c2)Br)cc(c1OC)OC

Physicochemical Properties	
Formula	C17H16Br2O3
Molecular weight	428.12 g/mol
Num. heavy atoms	22
Num. arom. heavy atoms	12
Fraction Csp3	0.18
Num. rotatable bonds	5
Num. H-bond acceptors	3
Num. H-bond donors	0
Molar Refractivity	96.69
TPSA	27.69 Å²

Lipophilicity	
Log $P_{o/w}$ (iLOGP)	4.18
Log $P_{o/w}$ (XLOGP3)	5.49
Log $P_{o/w}$ (WLOGP)	5.19
Log $P_{o/w}$ (MLOGP)	4.24
Log $P_{o/w}$ (SILICOS-IT)	5.50
Consensus Log $P_{o/w}$	4.92

Water Solubility	
Log *S* (ESOL)	-6.03
Solubility	4.03e-04 mg/ml ; 9.40e-07 mol/l
Class	Poorly soluble
Log *S* (Ali)	-5.83
Solubility	6.34e-04 mg/ml ; 1.48e-06 mol/l
Class	Moderately soluble
Log *S* (SILICOS-IT)	-6.99
Solubility	4.40e-05 mg/ml ; 1.03e-07 mol/l
Class	Poorly soluble

Pharmacokinetics	
GI absorption	High
BBB permeant	Yes
P-gp substrate	No
CYP1A2 inhibitor	Yes
CYP2C19 inhibitor	Yes
CYP2C9 inhibitor	Yes
CYP2D6 inhibitor	Yes
CYP3A4 inhibitor	No
Log K_p (skin permeation)	-5.01 cm/s

Druglikeness	
Lipinski	Yes; 1 violation: MLOGP>4.15
Ghose	Yes
Veber	Yes
Egan	Yes
Muegge	No; 1 violation: XLOGP3>5
Bioavailability Score	0.55

Medicinal Chemistry	
PAINS	0 alert
Brenk	1 alert: stilbene
Leadlikeness	No; 2 violations: MW>350, XLOGP3>3.5
Synthetic accessibility	2.75

Figure S61. SwissDrugDesign module prediction for compound **6**

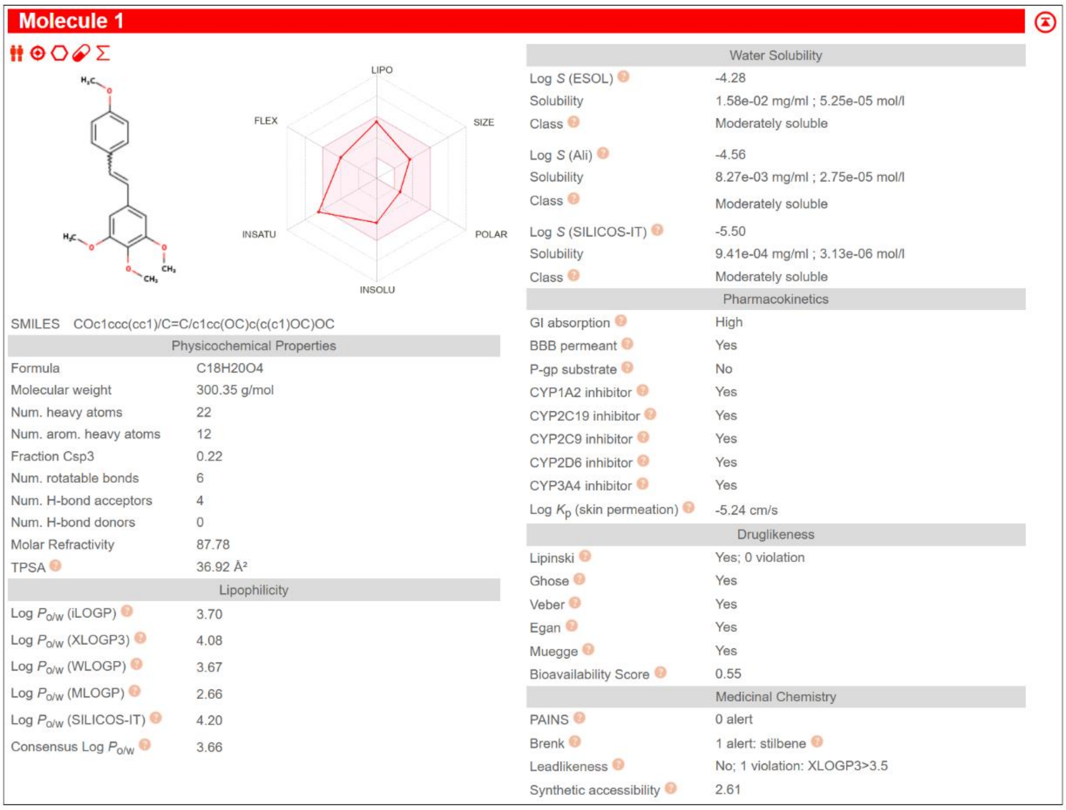

SMILES COc1ccc(cc1)/C=C/c1cc(OC)c(c(c1)OC)OC

Physicochemical Properties	
Formula	C18H20O4
Molecular weight	300.35 g/mol
Num. heavy atoms	22
Num. arom. heavy atoms	12
Fraction Csp3	0.22
Num. rotatable bonds	6
Num. H-bond acceptors	4
Num. H-bond donors	0
Molar Refractivity	87.78
TPSA	36.92 Å²

Lipophilicity	
Log $P_{o/w}$ (iLOGP)	3.70
Log $P_{o/w}$ (XLOGP3)	4.08
Log $P_{o/w}$ (WLOGP)	3.67
Log $P_{o/w}$ (MLOGP)	2.66
Log $P_{o/w}$ (SILICOS-IT)	4.20
Consensus Log $P_{o/w}$	3.66

Water Solubility	
Log *S* (ESOL)	-4.28
Solubility	1.58e-02 mg/ml ; 5.25e-05 mol/l
Class	Moderately soluble
Log *S* (Ali)	-4.56
Solubility	8.27e-03 mg/ml ; 2.75e-05 mol/l
Class	Moderately soluble
Log *S* (SILICOS-IT)	-5.50
Solubility	9.41e-04 mg/ml ; 3.13e-06 mol/l
Class	Moderately soluble

Pharmacokinetics	
GI absorption	High
BBB permeant	Yes
P-gp substrate	No
CYP1A2 inhibitor	Yes
CYP2C19 inhibitor	Yes
CYP2C9 inhibitor	Yes
CYP2D6 inhibitor	Yes
CYP3A4 inhibitor	Yes
Log K_p (skin permeation)	-5.24 cm/s

Druglikeness	
Lipinski	Yes; 0 violation
Ghose	Yes
Veber	Yes
Egan	Yes
Muegge	Yes
Bioavailability Score	0.55

Medicinal Chemistry	
PAINS	0 alert
Brenk	1 alert: stilbene
Leadlikeness	No; 1 violation: XLOGP3>3.5
Synthetic accessibility	2.61

Figure S62. SwissDrugDesign module prediction for compound **7**

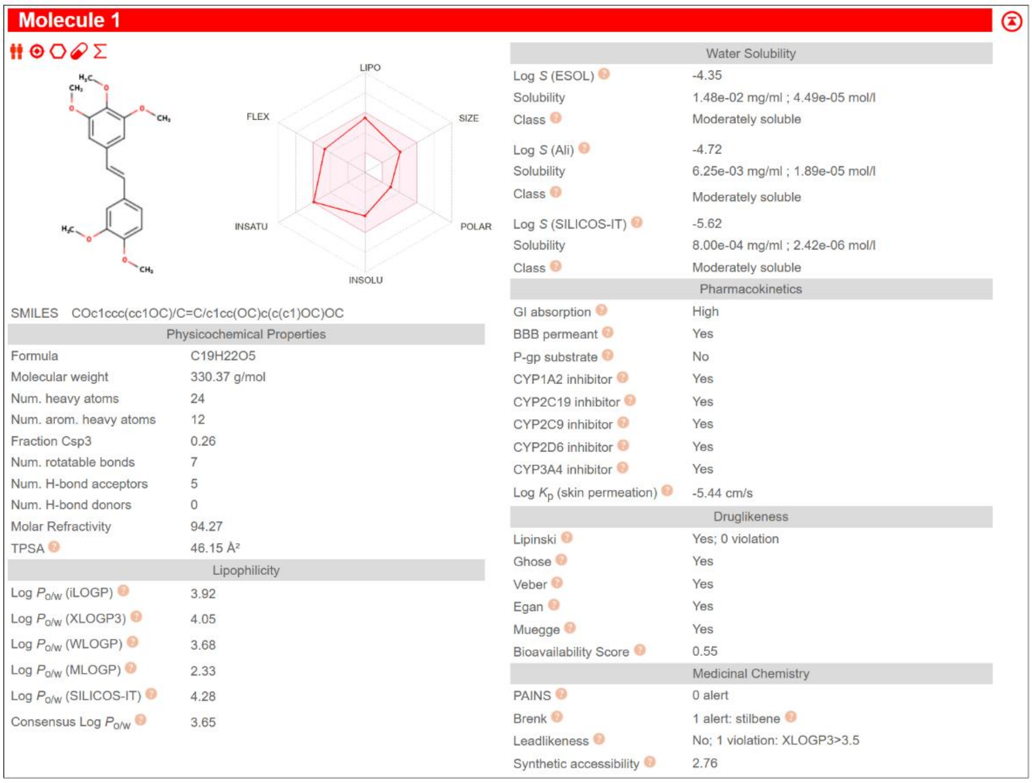

SMILES COc1ccc(cc1OC)/C=C/c1cc(OC)c(c(c1)OC)OC

Physicochemical Properties	
Formula	C19H22O5
Molecular weight	330.37 g/mol
Num. heavy atoms	24
Num. arom. heavy atoms	12
Fraction Csp3	0.26
Num. rotatable bonds	7
Num. H-bond acceptors	5
Num. H-bond donors	0
Molar Refractivity	94.27
TPSA	46.15 Å²

Lipophilicity	
Log $P_{o/w}$ (iLOGP)	3.92
Log $P_{o/w}$ (XLOGP3)	4.05
Log $P_{o/w}$ (WLOGP)	3.68
Log $P_{o/w}$ (MLOGP)	2.33
Log $P_{o/w}$ (SILICOS-IT)	4.28
Consensus Log $P_{o/w}$	3.65

Water Solubility	
Log *S* (ESOL)	-4.35
Solubility	1.48e-02 mg/ml ; 4.49e-05 mol/l
Class	Moderately soluble
Log *S* (Ali)	-4.72
Solubility	6.25e-03 mg/ml ; 1.89e-05 mol/l
Class	Moderately soluble
Log *S* (SILICOS-IT)	-5.62
Solubility	8.00e-04 mg/ml ; 2.42e-06 mol/l
Class	Moderately soluble

Pharmacokinetics	
GI absorption	High
BBB permeant	Yes
P-gp substrate	No
CYP1A2 inhibitor	Yes
CYP2C19 inhibitor	Yes
CYP2C9 inhibitor	Yes
CYP2D6 inhibitor	Yes
CYP3A4 inhibitor	Yes
Log K_p (skin permeation)	-5.44 cm/s

Druglikeness	
Lipinski	Yes; 0 violation
Ghose	Yes
Veber	Yes
Egan	Yes
Muegge	Yes
Bioavailability Score	0.55

Medicinal Chemistry	
PAINS	0 alert
Brenk	1 alert: stilbene
Leadlikeness	No; 1 violation: XLOGP3>3.5
Synthetic accessibility	2.76

Figure S63. SwissDrugDesign module prediction for compound **8**

Molecule 1

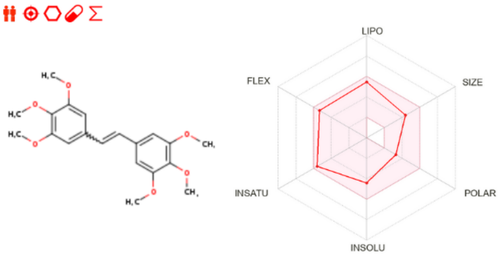

SMILES COc1cc(/C=C/c2cc(OC)c(c(c2)OC)OC)cc(c1OC)OC

Physicochemical Properties	
Formula	C20H24O6
Molecular weight	360.40 g/mol
Num. heavy atoms	26
Num. arom. heavy atoms	12
Fraction Csp3	0.30
Num. rotatable bonds	8
Num. H-bond acceptors	6
Num. H-bond donors	0
Molar Refractivity	100.76
TPSA	55.38 Å²

Lipophilicity	
Log $P_{o/w}$ (iLOGP)	4.08
Log $P_{o/w}$ (XLOGP3)	4.02
Log $P_{o/w}$ (WLOGP)	3.69
Log $P_{o/w}$ (MLOGP)	2.01
Log $P_{o/w}$ (SILICOS-IT)	4.36
Consensus Log $P_{o/w}$	3.63

Water Solubility	
Log *S* (ESOL)	-4.42
Solubility	1.37e-02 mg/ml ; 3.80e-05 mol/l
Class	Moderately soluble
Log *S* (Ali)	-4.89
Solubility	4.69e-03 mg/ml ; 1.30e-05 mol/l
Class	Moderately soluble
Log *S* (SILICOS-IT)	-5.72
Solubility	6.80e-04 mg/ml ; 1.89e-06 mol/l
Class	Moderately soluble

Pharmacokinetics	
GI absorption	High
BBB permeant	Yes
P-gp substrate	No
CYP1A2 inhibitor	No
CYP2C19 inhibitor	Yes
CYP2C9 inhibitor	Yes
CYP2D6 inhibitor	Yes
CYP3A4 inhibitor	Yes
Log K_p (skin permeation)	-5.64 cm/s

Druglikeness	
Lipinski	Yes; 0 violation
Ghose	Yes
Veber	Yes
Egan	Yes
Muegge	Yes
Bioavailability Score	0.55

Medicinal Chemistry	
PAINS	0 alert
Brenk	1 alert: stilbene
Leadlikeness	No; 3 violations: MW>350, Rotors>7, XLOGP3>3.5
Synthetic accessibility	2.90

Figure S64. SwissDrugDesign module prediction for compound **9**

Molecule 1

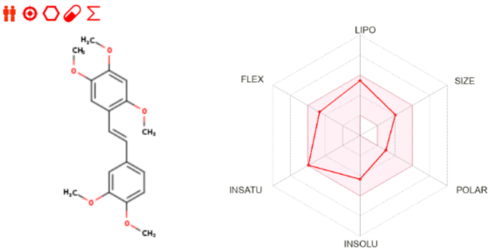

SMILES COc1cc(OC)c(cc1/C=C/c1ccc(c(c1)OC)OC)OC

Physicochemical Properties	
Formula	C19H22O5
Molecular weight	330.37 g/mol
Num. heavy atoms	24
Num. arom. heavy atoms	12
Fraction Csp3	0.26
Num. rotatable bonds	7
Num. H-bond acceptors	5
Num. H-bond donors	0
Molar Refractivity	94.27
TPSA	46.15 Å²

Lipophilicity	
Log $P_{o/w}$ (iLOGP)	3.92
Log $P_{o/w}$ (XLOGP3)	4.05
Log $P_{o/w}$ (WLOGP)	3.68
Log $P_{o/w}$ (MLOGP)	2.33
Log $P_{o/w}$ (SILICOS-IT)	4.28
Consensus Log $P_{o/w}$	3.65

Water Solubility	
Log *S* (ESOL)	-4.35
Solubility	1.48e-02 mg/ml ; 4.49e-05 mol/l
Class	Moderately soluble
Log *S* (Ali)	-4.72
Solubility	6.25e-03 mg/ml ; 1.89e-05 mol/l
Class	Moderately soluble
Log *S* (SILICOS-IT)	-5.62
Solubility	8.00e-04 mg/ml ; 2.42e-06 mol/l
Class	Moderately soluble

Pharmacokinetics	
GI absorption	High
BBB permeant	Yes
P-gp substrate	No
CYP1A2 inhibitor	Yes
CYP2C19 inhibitor	Yes
CYP2C9 inhibitor	Yes
CYP2D6 inhibitor	Yes
CYP3A4 inhibitor	Yes
Log K_p (skin permeation)	-5.44 cm/s

Druglikeness	
Lipinski	Yes; 0 violation
Ghose	Yes
Veber	Yes
Egan	Yes
Muegge	Yes
Bioavailability Score	0.55

Medicinal Chemistry	
PAINS	0 alert
Brenk	1 alert: stilbene
Leadlikeness	No; 1 violation: XLOGP3>3.5
Synthetic accessibility	3.01

Figure S65. SwissDrugDesign module prediction for compound **10**

Molecule 1

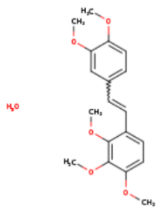

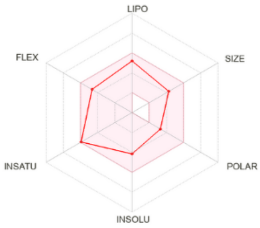

SMILES COc1ccc(cc1OC)/C=C/c1ccc(c(c1OC)OC)OC.O

Physicochemical Properties	
Formula	C19H24O6
Molecular weight	348.39 g/mol
Num. heavy atoms	25
Num. arom. heavy atoms	12
Fraction Csp3	0.26
Num. rotatable bonds	7
Num. H-bond acceptors	6
Num. H-bond donors	1
Molar Refractivity	97.32
TPSA	55.38 Å²

Lipophilicity	
Log $P_{o/w}$ (iLOGP)	3.85
Log $P_{o/w}$ (XLOGP3)	3.57
Log $P_{o/w}$ (WLOGP)	3.62
Log $P_{o/w}$ (MLOGP)	1.52
Log $P_{o/w}$ (SILICOS-IT)	4.28
Consensus Log $P_{o/w}$	3.37

Water Solubility	
Log *S* (ESOL)	-4.14
Solubility	2.51e-02 mg/ml ; 7.21e-05 mol/l
Class	Moderately soluble
Log *S* (Ali)	-4.42
Solubility	1.33e-02 mg/ml ; 3.81e-05 mol/l
Class	Moderately soluble
Log *S* (SILICOS-IT)	-5.62
Solubility	8.43e-04 mg/ml ; 2.42e-06 mol/l
Class	Moderately soluble

Pharmacokinetics	
GI absorption	High
BBB permeant	Yes
P-gp substrate	No
CYP1A2 inhibitor	No
CYP2C19 inhibitor	Yes
CYP2C9 inhibitor	Yes
CYP2D6 inhibitor	Yes
CYP3A4 inhibitor	Yes
Log K_p (skin permeation)	-5.89 cm/s

Druglikeness	
Lipinski	Yes; 0 violation
Ghose	Yes
Veber	Yes
Egan	Yes
Muegge	Yes
Bioavailability Score	0.55

Medicinal Chemistry	
PAINS	0 alert
Brenk	1 alert: stilbene
Leadlikeness	No; 1 violation: XLOGP3>3.5
Synthetic accessibility	3.10

Figure S66. SwissDrugDesign module prediction for compound **11**

Molecule 1

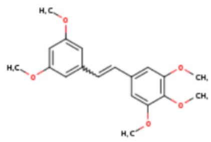

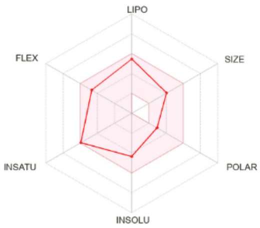

SMILES COc1cc(/C=C/c2cc(OC)c(c(c2)OC)OC)cc(c1)OC

Physicochemical Properties	
Formula	C19H22O5
Molecular weight	330.37 g/mol
Num. heavy atoms	24
Num. arom. heavy atoms	12
Fraction Csp3	0.26
Num. rotatable bonds	7
Num. H-bond acceptors	5
Num. H-bond donors	0
Molar Refractivity	94.27
TPSA	46.15 Å²

Lipophilicity	
Log $P_{o/w}$ (iLOGP)	4.06
Log $P_{o/w}$ (XLOGP3)	4.05
Log $P_{o/w}$ (WLOGP)	3.68
Log $P_{o/w}$ (MLOGP)	2.33
Log $P_{o/w}$ (SILICOS-IT)	4.28
Consensus Log $P_{o/w}$	3.68

Water Solubility	
Log *S* (ESOL)	-4.35
Solubility	1.48e-02 mg/ml ; 4.49e-05 mol/l
Class	Moderately soluble
Log *S* (Ali)	-4.72
Solubility	6.25e-03 mg/ml ; 1.89e-05 mol/l
Class	Moderately soluble
Log *S* (SILICOS-IT)	-5.62
Solubility	8.00e-04 mg/ml ; 2.42e-06 mol/l
Class	Moderately soluble

Pharmacokinetics	
GI absorption	High
BBB permeant	Yes
P-gp substrate	No
CYP1A2 inhibitor	Yes
CYP2C19 inhibitor	Yes
CYP2C9 inhibitor	Yes
CYP2D6 inhibitor	Yes
CYP3A4 inhibitor	Yes
Log K_p (skin permeation)	-5.44 cm/s

Druglikeness	
Lipinski	Yes; 0 violation
Ghose	Yes
Veber	Yes
Egan	Yes
Muegge	Yes
Bioavailability Score	0.55

Medicinal Chemistry	
PAINS	0 alert
Brenk	1 alert: stilbene
Leadlikeness	No; 1 violation: XLOGP3>3.5
Synthetic accessibility	2.76

Figure S67. SwissDrugDesign module prediction for compound **12**

Table S5. The five most probable molecular targets predicted by SwissTargetPrediction model for **1**.

SwissTargetPrediction

Target	Common name	Uniprot ID	ChEMBL ID	Target Class	Probability*	Known actives (3D/2D)
Aryl hydrocarbon receptor	AHR	P35869	CHEMBL3201	Transcription factor	0.106099949133	0 / 4
Cyclooxygenase-1	PTGS1	P23219	CHEMBL221	Oxidoreductase	0.106099949133	42 / 20
Cyclooxygenase-2	PTGS2	P35354	CHEMBL230	Oxidoreductase	0.0978745343258	242 / 53
P-glycoprotein 1	ABCB1	P08183	CHEMBL4302	Primary active transporter	0.0978745343258	40 / 10
Beta amyloid A4 protein	APP	P05067	CHEMBL2487	Membrane receptor	0.0978745343258	27 / 43

Table S6. The five most probable molecular targets predicted by SwissTargetPrediction model for **2**.

SwissTargetPrediction

Target	Common name	Uniprot ID	ChEMBL ID	Target Class	Probability*	Known actives (3D/2D)
Tubulin beta-1 chain	TUBB1	Q9H4B7	CHEMBL1915	Structural protein	0.139088623131	10 / 25
P-glycoprotein 1	ABCB1	P08183	CHEMBL4302	Primary active transporter	0.0972399887602	14 / 3
Beta amyloid A4 protein	APP	P05067	CHEMBL2487	Membrane receptor	0.0972399887602	16 / 28
Tubulin beta-3 chain	TUBB3	Q13509	CHEMBL2597	Structural protein	0.0972399887602	0 / 1
Cyclooxygenase-1	PTGS1	P23219	CHEMBL221	Oxidoreductase	0.0972399887602	0 / 11

Table S7. The five most probable molecular targets predicted by SwissTargetPrediction model for **3**.

SwissTargetPrediction

Target	Common name	Uniprot ID	ChEMBL ID	Target Class	Probability*	Known actives (3D/2D)
Tubulin beta-1 chain	TUBB1	Q9H4B7	CHEMBL1915	Structural protein	0.742679213102	30 / 27
P-glycoprotein 1	ABCB1	P08183	CHEMBL4302	Primary active transporter	0.112748418065	86 / 3
Beta amyloid A4 protein	APP	P05067	CHEMBL2487	Membrane receptor	0.112748418065	71 / 29
Cytochrome P450 1B1	CYP1B1	Q16678	CHEMBL4878	Cytochrome P450	0.104671941128	37 / 25
Estrogen receptor alpha	ESR1	P03372	CHEMBL206	Nuclear receptor	0.104671941128	32 / 8

Table S8. The five most probable molecular targets predicted by SwissTargetPrediction moled for **4**.

SwissTargetPrediction

Target	Common name	Uniprot ID	ChEMBL ID	Target Class	Probability*	Known actives (3D/2D)
Tubulin beta-1 chain	TUBB1	Q9H4B7	CHEMBL1915	Structural protein	0.659830583012	14 / 28
P-glycoprotein 1	ABCB1	P08183	CHEMBL4302	Primary active transporter	0.109945769839	30 / 5
Beta amyloid A4 protein	APP	P05067	CHEMBL2487	Membrane receptor	0.109945769839	39 / 38
Cytochrome P450 1B1	CYP1B1	Q16678	CHEMBL4878	Cytochrome P450	0.109945769839	11 / 25
Tubulin beta-3 chain	TUBB3	Q13509	CHEMBL2597	Structural protein	0.101613854776	0 / 1

Table S9. The five most probable molecular targets predicted by SwissTargetPrediction moled for **5**.

SwissTargetPrediction

Target	Common name	Uniprot ID	ChEMBL ID	Target Class	Probability*	Known actives (3D/2D)
P-glycoprotein 1	ABCB1	P08183	CHEMBL4302	Primary active transporter	0.0972399887602	14 / 5
Tubulin beta-3 chain	TUBB3	Q13509	CHEMBL2597	Structural protein	0.0972399887602	0 / 1
Tubulin beta-1 chain	TUBB1	Q9H4B7	CHEMBL1915	Structural protein	0.0972399887602	10 / 28
Cyclooxygenase-1	PTGS1	P23219	CHEMBL221	Oxidoreductase	0.0972399887602	0 / 13
Aryl hydrocarbon receptor	AHR	P35869	CHEMBL3201	Transcription factor	0.0972399887602	0 / 4

Table S10. The five most probable molecular targets predicted by SwissTargetPrediction moled for **6**.

SwissTargetPrediction

Target	Common name	Uniprot ID	ChEMBL ID	Target Class	Probability*	Known actives (3D/2D)
Tubulin beta-1 chain	TUBB1	Q9H4B7	CHEMBL1915	Structural protein	0.253686853584	7 / 28
Beta amyloid A4 protein	APP	P05067	CHEMBL2487	Membrane receptor	0.119403562123	38 / 40
Tubulin beta-3 chain	TUBB3	Q13509	CHEMBL2597	Structural protein	0.11150186548	0 / 1
Cyclooxygenase-1	PTGS1	P23219	CHEMBL221	Oxidoreductase	0.11150186548	94 / 14
Cyclooxygenase-2	PTGS2	P35354	CHEMBL230	Oxidoreductase	0.11150186548	424 / 41

Table S11. The five most probable molecular targets predicted by SwissTargetPrediction moled for **7**.

SwissTargetPrediction

Target	Common name	Uniprot ID	ChEMBL ID	Target Class	Probability*	Known actives (3D/2D)
Tubulin beta-1 chain	TUBB1	Q9H4B7	CHEMBL1915	Structural protein	0.672321427696	14 / 32
P-glycoprotein 1	ABCB1	P08183	CHEMBL4302	Primary active transporter	0.506417696451	35 / 10
Cytochrome P450 1B1	CYP1B1	Q16678	CHEMBL4878	Cytochrome P450	0.340526084149	11 / 25
Tubulin beta-3 chain	TUBB3	Q13509	CHEMBL2597	Structural protein	0.340526084149	0 / 1
Quinone reductase 2	NQO2	P16083	CHEMBL3959	Enzyme	0.222013735009	6 / 18

Table S12. The five most probable molecular targets predicted by SwissTargetPrediction moled for **8**.

SwissTargetPrediction

Target	Common name	Uniprot ID	ChEMBL ID	Target Class	Probability*	Known actives (3D/2D)
P-glycoprotein 1	ABCB1	P08183	CHEMBL4302	Primary active transporter	0.540820499434	20 / 10
Tubulin beta-3 chain	TUBB3	Q13509	CHEMBL2597	Structural protein	0.330703494192	0 / 1
Tubulin beta-1 chain	TUBB1	Q9H4B7	CHEMBL1915	Structural protein	0.258108849137	12 / 32
Cyclooxygenase-1	PTGS1	P23219	CHEMBL221	Oxidoreductase	0.225803139262	0 / 26
Cytochrome P450 1B1	CYP1B1	Q16678	CHEMBL4878	Cytochrome P450	0.185437279733	9 / 25

Table S13. The five most probable molecular targets predicted by SwissTargetPrediction moled for **10**.

SwissTargetPrediction

Target	Common name	Uniprot ID	ChEMBL ID	Target Class	Probability*	Known actives (3D/2D)
Cytochrome P450 1A1	CYP1A1	P04798	CHEMBL2231	Cytochrome P450	0.849652886369	7 / 9
Cytochrome P450 1B1	CYP1B1	Q16678	CHEMBL4878	Cytochrome P450	0.849652886369	26 / 25
Cytochrome P450 1A2	CYP1A2	P05177	CHEMBL3356	Cytochrome P450	0.209643327323	3 / 5
HMG-CoA reductase	HMGCR	P04035	CHEMBL402	Oxidoreductase	0.201584697716	0 / 2
Tubulin beta-1 chain	TUBB1	Q9H4B7	CHEMBL1915	Structural protein	0.13696956545	20 / 30

6. HPLC data

Table S14. HPLC data for compound **1-12**.

Compound number	Retetion time [min]	Purity [%]
**1**	3.43	99.64
**2**	2.77	97.89
**3**	4.28	98.58
**4**	2.97	100
**5**	2.79	99.43
**6**	5.54	100
**7**	2.59	99.54
**8**	2.29	100
**9**	2.33	99.23
**10**	2.72	100
**11**	2.51	100
**12**	2.57	99.94

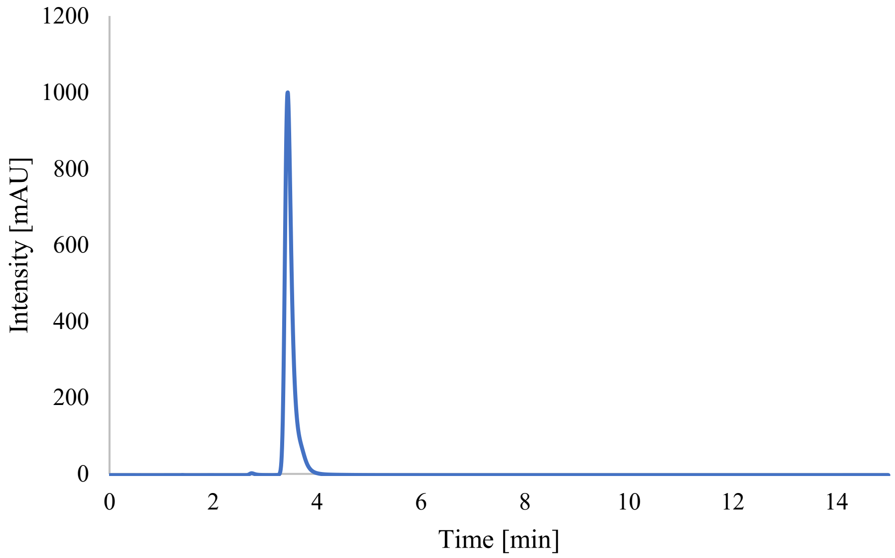

Figure. S68. HPLC chromatogram for stilbene **1**.

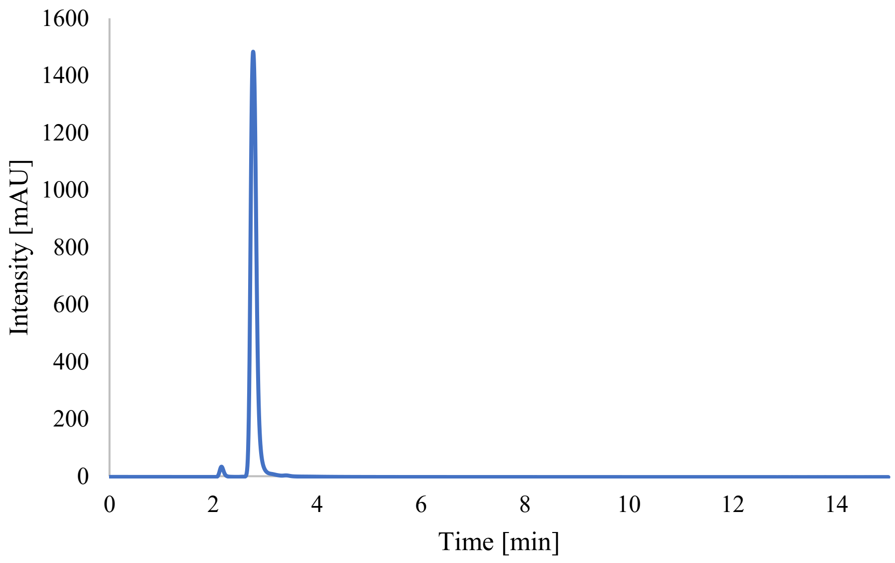

Figure. S69. HPLC chromatogram for stilbene **2**.

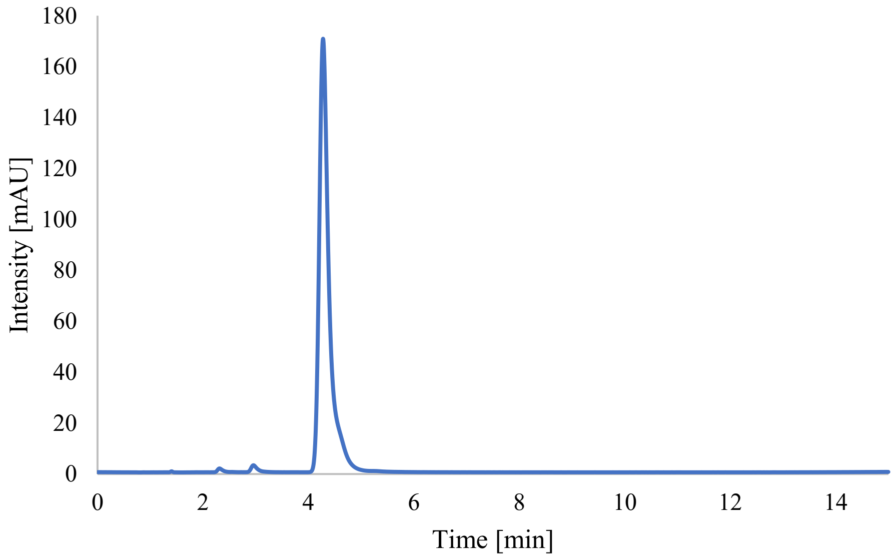

Figure. S70. HPLC chromatogram for stilbene **3**.

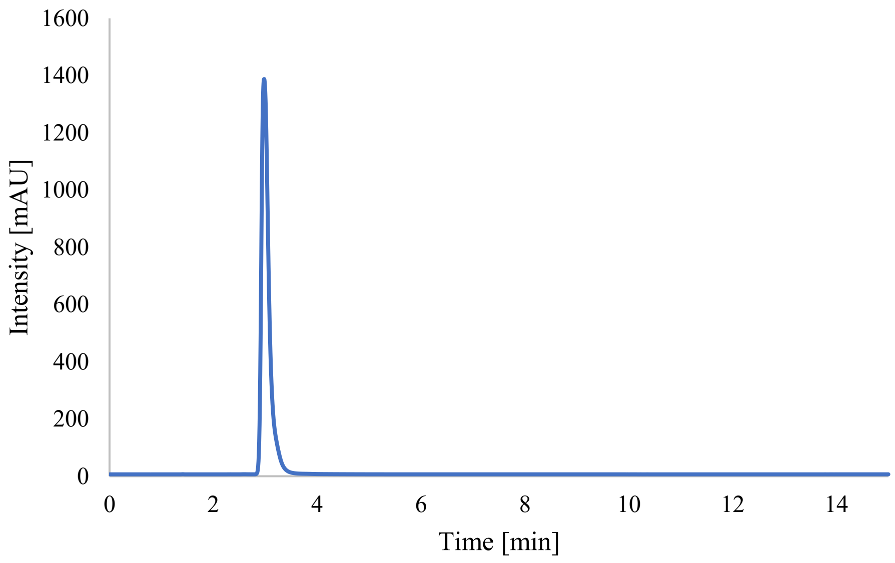

Figure. S71. HPLC chromatogram for stilbene **4**.

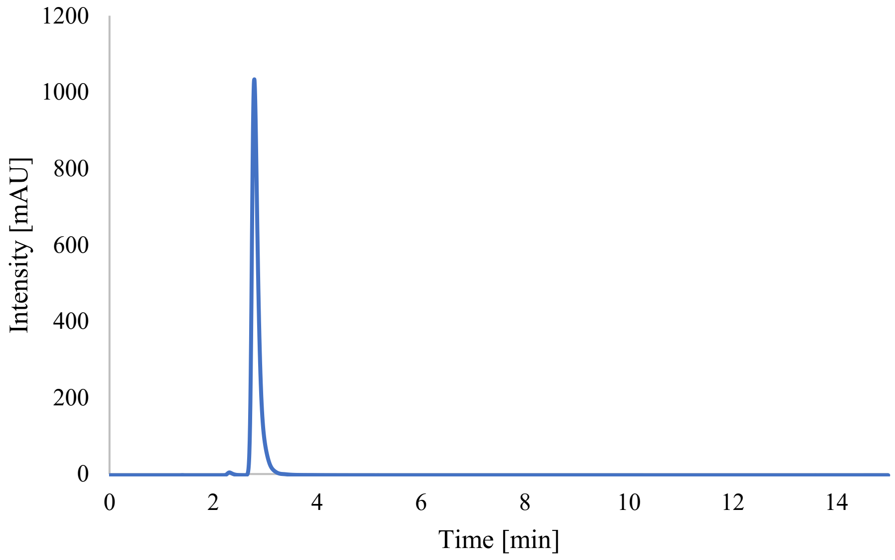

Figure. S72. HPLC chromatogram for stilbene **5**.

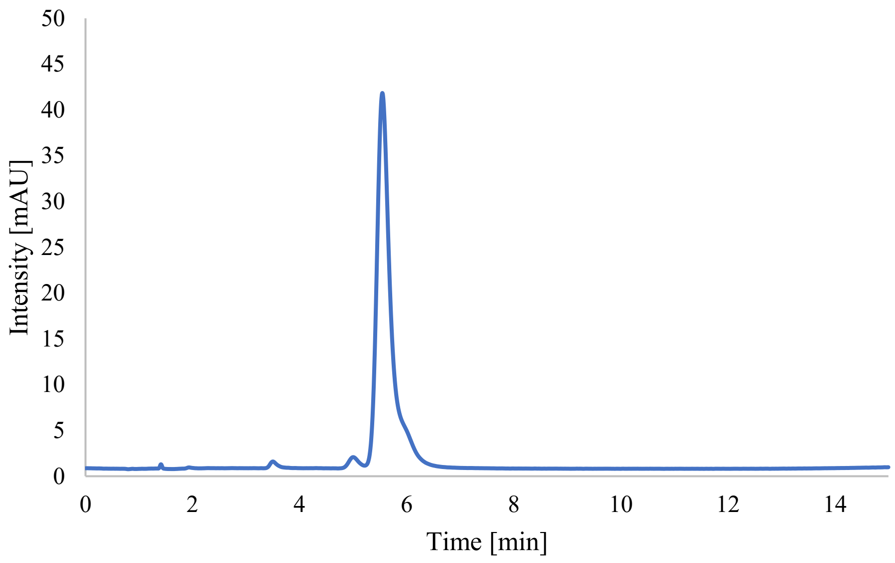

Figure. S73. HPLC chromatogram for stilbene **6**.

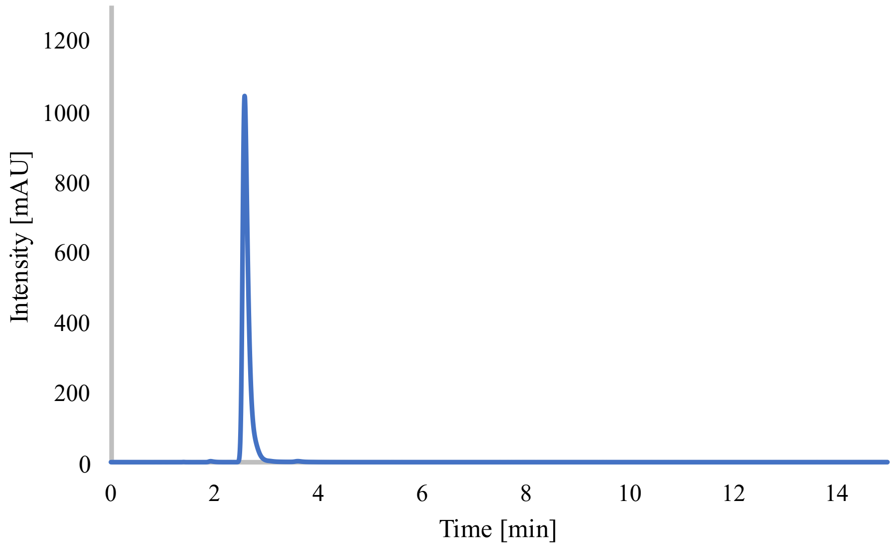

Figure. S74. HPLC chromatogram for stilbene **7**.

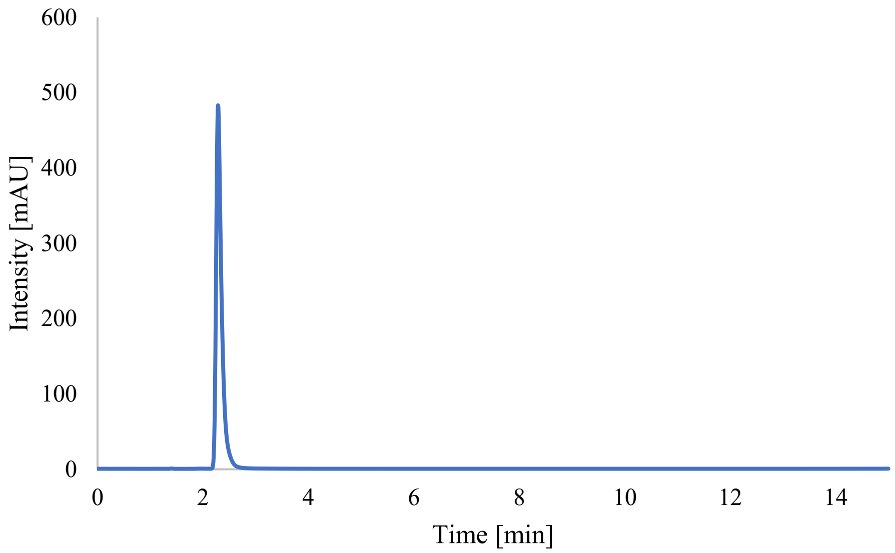

Figure. S75. HPLC chromatogram for stilbene **8**.

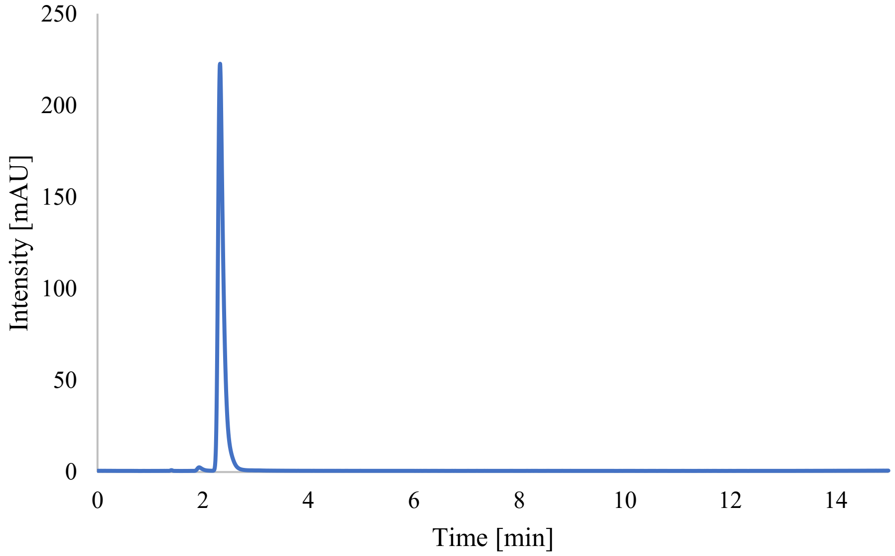

Figure. S76. HPLC chromatogram for stilbene **9**.

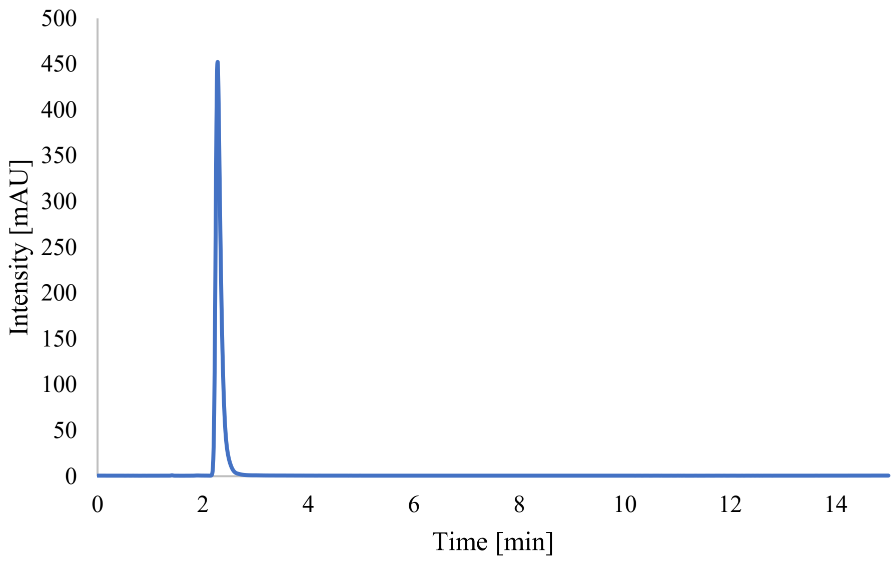

Figure. S77. HPLC chromatogram for stilbene **10**.

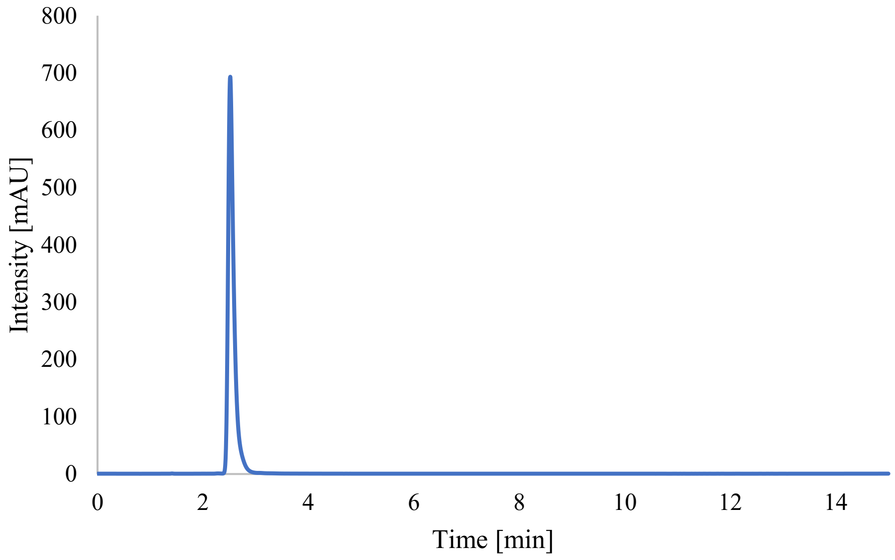

Figure. S78. HPLC chromatogram for stilbene **11**.

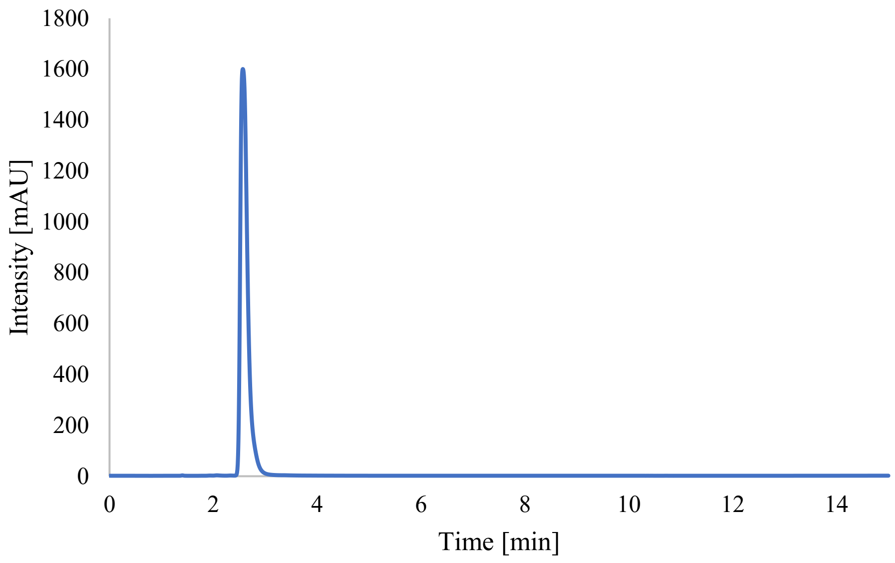

Figure. S79. HPLC chromatogram for stilbene **12**.
